# Algorithmic Self-Assembly of DNA Sierpinski Triangles

**DOI:** 10.1371/journal.pbio.0020424

**Published:** 2004-12-07

**Authors:** Paul W. K Rothemund, Nick Papadakis, Erik Winfree

**Affiliations:** **1**Computation and Neural Systems, California Institute of TechnologyPasadena, CaliforniaUnited States of America; **2**Computer Science, California Institute of TechnologyPasadena, CaliforniaUnited States of America

## Abstract

Algorithms and information, fundamental to technological and biological organization, are also an essential aspect of many elementary physical phenomena, such as molecular self-assembly. Here we report the molecular realization, using two-dimensional self-assembly of DNA tiles, of a cellular automaton whose update rule computes the binary function XOR and thus fabricates a fractal pattern—a Sierpinski triangle—as it grows. To achieve this, abstract tiles were translated into DNA tiles based on double-crossover motifs. Serving as input for the computation, long single-stranded DNA molecules were used to nucleate growth of tiles into algorithmic crystals. For both of two independent molecular realizations, atomic force microscopy revealed recognizable Sierpinski triangles containing 100–200 correct tiles. Error rates during assembly appear to range from 1% to 10%. Although imperfect, the growth of Sierpinski triangles demonstrates all the necessary mechanisms for the molecular implementation of arbitrary cellular automata. This shows that engineered DNA self-assembly can be treated as a Turing-universal biomolecular system, capable of implementing any desired algorithm for computation or construction tasks.

## Abstract 2

Engineered DNA self-assembly to produce a fractal pattern demonstrates all the necessary mechanisms for the molecular implementation of arbitrary cellular automata

## Introduction

How is complex organization produced and maintained by physical processes? One may look to biology, where we find the most sophisticated organization of matter, often spanning more than 24 orders of magnitude from component molecules (0.1 attograms) to entire organism (100 kilograms). This organization is information-based: DNA sequences refined by evolution encode both the components and the processes that guide their development into an organism—the developmental program. For a language to describe this carefully orchestrated organization, it is tempting to turn to computer science, where the concepts of programming languages, data structures, and algorithms are used to specify complex organization of information and behavior. Indeed, the importance of universal computation for autonomous fabrication tasks was recognized in von Neumann's seminal work on self-reproducing automata, where he postulated a *universal constructor* that, by reading an input tape specifying an algorithm for what to build, could carry out the commands necessary to construct an arbitrary object (von Neumann 1966). If algorithmic concepts can be successfully adapted to the molecular context, the algorithm would join energy and entropy as essential concepts for understanding how physical processes create order. Unfortunately, the study of molecular algorithms has been hampered by the lack of suitable physical systems on which to hone such a theory: nature provides us with elementary chemical reactions too simple to program, full-blown life too complex to use as a model system, and few systems in between. This gap may be explored by synthesizing programmable biochemical systems in vitro, where we can implement and study a variety of molecular algorithms ranging gradually from simple to complex.

Biomolecular self-assembly is particularly attractive for the exploration of molecular algorithms that control nanofabrication tasks. Attesting to its power, self-assembly is used pervasively in biology to create such structures as virus capsids, microtubules, and flagella. In each case, the binding interactions between a small number of protein species is sufficient to dictate the form of the final structure, often via a complex sequence of cooperative assembly steps. This can be viewed loosely as a form of programmable nanofabrication, where the program is the set of molecular species involved. For synthetic approaches, Seeman (1982, 2003) has demonstrated that DNA provides an alternative to protein that can be readily programmed by Watson–Crick complementarity. A seminal paper by Adleman (1994) used one-dimensional (1D) DNA self-assembly to operate as a finite-state machine, establishing the first experimental connection between DNA self-assembly and computation. This work inspired a theoretical proposal (Winfree 1996) that builds on Wang's (1961, 1962) embedding of computation in geometrical tilings to show that two-dimensional (2D) self-assembly of DNA can perform Turing-universal computation—which implies that *any* algorithm can in principle be embedded in, and guide, a potentially aperiodic crystallization process. In this “algorithmic self-assembly” paradigm, a set of molecular Wang tiles is viewed as the program for a particular computation or molecular fabrication task (Reif 1999; Rothemund and Winfree 2000; Adleman et al. 2001). (This framework differs from previous approaches relating tiling theory to crystalline ground-states [Radin 1985] in that kinetic phenomena are essential here.) Whereas 1D algorithmic self-assembly offers limited computational power (Winfree et al. 1998b) and has been experimentally demonstrated (Adleman 1994; Mao et al. 2000), 2D algorithmic self-assembly offers not only new capabilities for computation and construction, but also presents a new range of physical phenomena and experimental challenges as well.

**Figure 1:**
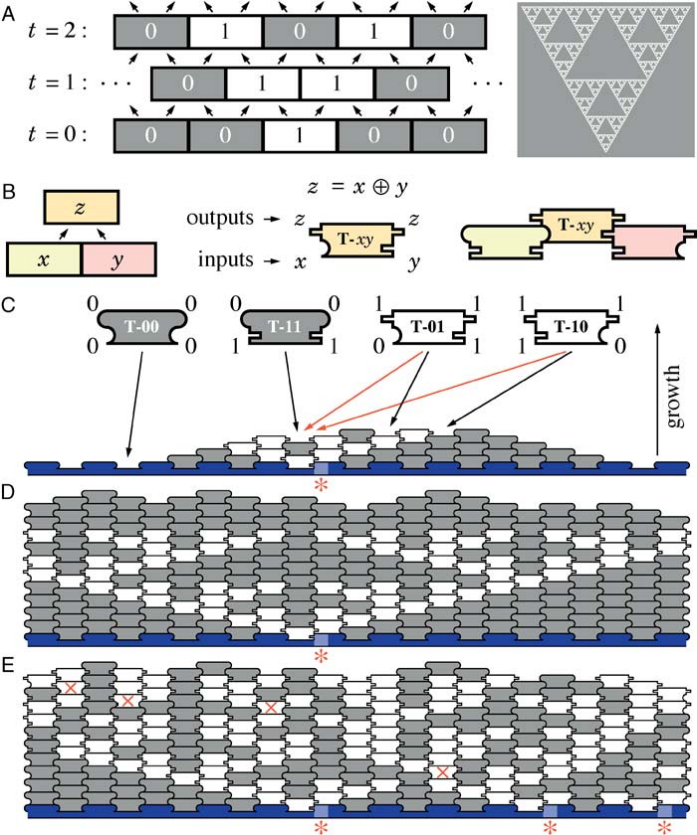
The XOR Cellular Automaton and Its Implementation by Tile-Based Self-Assembly (A) Left: three time steps of its execution drawn as a space–time history. Cells update synchronously according to XOR by the equation shown. Cells at even time steps are interleaved with those at odd time steps; arrows show propagation of information. Right: the Sierpinski triangle. (B) Translating the space–time history into a tiling. For each possible input pair, we generate a tile T-*xy* that bears the inputs represented as shapes on the lower half of each side and the output as shapes duplicated on the top half of each side. (C) The four Sierpinski rule tiles, T-00, T-11, T-01, and T-10, represent the four entries of the truth table for XOR: 0 ⊕ 0 = 0, 1 ⊕ 1 = 0, 0 ⊕ 1 = 1, and 1 ⊕ 0 = 1. Lower binding domains on the sides of tiles match input from the layer below; upper binding domains provide output to both neighbors on the layer above. Semicircular domains represent ‘0' and rectangular domains, ‘1'. Tiles that output ‘0' (T-00 and T-11) are gray, and we refer to them as ‘0' tiles. Tiles that output ‘1' (T-01 and T-10) are white and are referred to as ‘1' tiles. Initial conditions for the computation are provided by a nucleating structure (blue). Red asterisks indicate sites on the nucleating structure that bear a ‘1' binding domain; elsewhere, sites have all ‘0' binding domains. Black arrows indicate associations that would form two bonds; red arrows, associations that would form one bond. (D) Error-free growth results in the Sierpinski pattern. (E) Error-prone growth from a nucleating structure with three ‘1' domains. Red crosses indicate four mismatch errors.

A natural Turing-universal model of computation that can be implemented by 2D algorithmic self-assembly is the class of 1D cellular automata. A simple but interesting choice for the local cellular automaton rule is the exclusive–or (XOR) function: at each time step, each cell is computed as the XOR of its two neighbors. Beginning with a row of all ‘0's punctuated by a single central ‘1,' snapshots of the cellular automaton's state at successive time steps may be stacked one on top of the other to produce a space–time history identical to Pascal's triangle (Bondarenko 1993) modulo 2 (Figure 1A, left), which is a discrete form of Sierpinski's fractal triangle (Figure 1A, right). To represent this cellular automaton as a tiling, each local context present in the space–time history must have a corresponding Wang tile whose shape represents the input and output occurring at that location (Figure 1B). Thus, we need four tiles, one for each entry of the truth table for XOR, and a linear input row representing the initial state of the cellular automaton (Figure 1C). Given these tiles and the input row there is a unique way to tile the upper half-plane without mismatches or missing tiles—the Sierpinski Tiling—which reproduces the cellular automaton's space–time history (Figure 1D).

Whereas execution of a cellular automaton occurs perfectly and synchronously, molecular self-assembly is asynchronous and may have many types of errors. To be successful, an implementation of cellular automata by molecular tiling must address four challenges: (1) The abstract tiles must be translated into molecules (molecular tiles) that readily form 2D crystals. (2) Molecular tiles must be programmed with specific binding domains that match the logic of the chosen abstract tiles. (3) The binding of molecular tiles must be sufficiently cooperative to enforce the correct order of assembly and prevent errors. (4) Assembly of molecular tiles must occur on a specified nucleating structure, and spurious nucleation must be suppressed. These properties are necessary and sufficient for implementing not only the XOR cellular automaton, but also any other 1D cellular automaton. All four have been shown individually: several types of DNA Wang tiles have been designed and shown to grow into micron-scale 2D periodic crystals (Winfree et al. 1998a; Mao et al. 1999; LaBean et al. 2000b); the interactions between these tiles can be programmed by sequence-specific hybridization (Winfree et al. 1998a; Mao et al. 2000); cooperative binding of multiple domains ensures specificity—the right tile attaches in the right place (Winfree et al. 1998b; Mao et al. 2000); and input to the self-assembly process can be provided by a single-stranded template (LaBean et al. 2000a; Yan et al. 2003a). Here we demonstrate, via self-assembly of Sierpinski triangles, that all four challenges can be simultaneously overcome, thus establishing all the mechanisms necessary to implement arbitrary cellular automata. The Sierpinski tiling, then, gives rise to a new type of aperiodic crystal—an algorithmic crystal.

## Results

### Modeling Tile-Based Self-Assembly

Preventing the types of errors mentioned above may seem impossible. For example, if a single binding domain is strong enough to hold a tile in place (red arrows in Figure 1C), then one would expect roughly 33% of tiles to mismatch with tiles in the layer below. Simulations of self-assembly shed light on how to avoid such dire circumstances. We use two levels of abstraction that isolate and address critical issues for the design and analysis of our algorithmic self-assembly experiments. How crystal morphology and patterning can be programmed by tile design in an inherently asynchronous assembly process is addressed by the abstract Tile Assembly Model (aTAM) (Winfree 1996, 1998a). To explore how physical parameters, such as tile concentration and temperature, affect crystal growth and influence error rates, we use the kinetic Tile Assembly Model (kTAM) based on reversible tile association and dissociation rates (Winfree 1998a).

**Figure 2:**
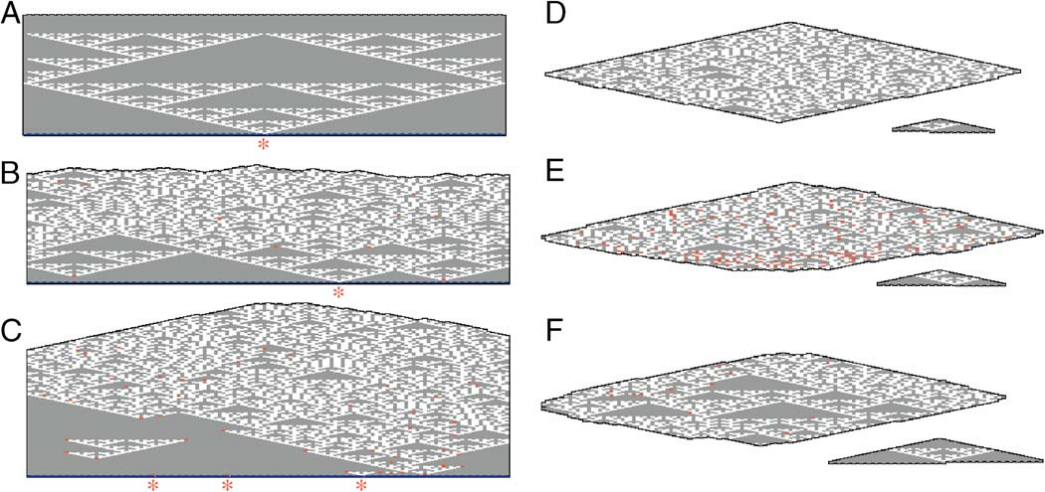
Typical kTAM Simulation Results (A) A roughly 130 × 70 subregion of an error-free templated crystal. (B) A subregion with 10 mismatch errors (0.1%), shown in red (both false ‘0's and false ‘1's). Grown at *G_mc_* = 17, *G_se_* = 8.8. Large all-zero patches near the template row are due to intact Sierpinski pattern; for simulations with these parameters, asymptotically only approximately 1% of T-00 tiles are in all-zero patches containing more than 90 tiles (referred to as large patches). (C) A subregion from a crystal grown with the T-00 and T-11 tiles at doubled concentration, on a slowly growing nucleating row. Mismatch errors (43 of them, i.e., 0.3%, during growth at *G_mc_* = 17, *G_se_* = 8.6) characteristically terminate the Sierpinski pattern at corners. Asymptotically, approximately 18% of T-00 tiles are in large patches. (D) An untemplated crystal with roughly 4000 tiles and no errors. Inset: The largest subregion of a perfect Sierpinski pattern is small. (E) An untemplated crystal with several errors, grown at *G_mc_* = 17, *G_se_* = 10.4. Note that growth in the reverse direction is more error-prone. Only approximately 1% of T-00 tiles are in large patches. (F) An untemplated crystal with few errors, grown at *G_mc_* = 17, *G_se_* = 8.6, with T-00 and T-11 at doubled stoichiometry. Note the large perfect subregion. Simulation was initiated by a preformed seed larger than the critical nucleus size (roughly 100 tiles). For these simulation parameters, approximately 25% of T-00 tiles are in large patches. According to the approximations used in Winfree (1998a), *G_mc_* = 17 corresponds to 0.8 μM, *G_se_* = 8.5 corresponds to 41.8 °C, and *G_se_* = 10.4 corresponds to 32.7 °C. The black outline around the crystals is for clarity; it does not represent tiles.

Control over the order of assembly is obtained by exploiting the cooperativity of binding. The aTAM models cooperativity via a threshold, τ, representing the number of bonds that must be made for an association event to be thermodynamically stable: a tile may be added to a crystal if at least τ binding domains match the existing crystal. Black arrows in Figure 1C indicate four potential association events that could occur at τ = 2; red arrows indicate two additional association events that would be permitted at τ = 1, but not at τ = 2. Simulation of cellular automata is designed to work at τ = 2. Isolated tiles cannot associate at τ = 2 and so growth and computation must begin with a preformed nucleating structure (Figure 1C, blue) that represents the input to the computation. Importantly, at τ = 2 no tile may be added until *both* preceding tiles are already present, guaranteeing a deterministic outcome despite the asynchronous order of events. Thus, assembly from an input row containing a solitary ‘1' domain produces the Sierpinski triangle pattern (Figures 1D and 2A) regardless of the order in which permitted associations occur. If a small number of additional τ = 1 associations are permitted to occur, then mismatches between neighboring tiles (mismatch errors) may result. In this case, or if there are several ‘1's in the input row, the resulting pattern can appear to be qualitatively different: owing to propagation of information and the linearity of XOR, the resulting pattern is the superposition of Sierpinski triangles initiated at input ‘1's and at mismatch error sites (see Figure 1E).

The rate at which such errors occur can be understood using the kTAM. In this model, all tiles (regardless of how well they match) may associate at a given site at a rate, *r_f_* , proportional to their concentration: *r_f_* = *k*[*tile*] = *ke* ^−*G_mc_*^ , where *k* is a forward rate constant and *G_mc_* > 0 is the nondimensionalized entropy lost due to association—thus it represents the monomer concentration. Dissociation rates depend on how many binding domains match correctly: a tile with *b* correctly matching binding domains has a dissociation rate given by *r_r,b_* =*ke* ^−*bG_se_*^ , where *G_se_* > 0 is the nondimensionalized free energy for a single binding domain—thus it represents the sticky end strength. *G_se_* decreases with increased temperature. Thus, if *G_se_<* *G_mc_* < 2*G_se_*, a reaction wherein the tile matches at a single domain would have *r_f_* < *r_r,_*_1_ and thus would be thermodynamically unfavorable, while a reaction wherein the tile matches at two domains would have *r_f_* > *r_r,_*_2_ and thus would be thermodynamically favorable. This model is a reasonable first-order approximation for the tile-based self-assembly of single crystals. For *G_mc_* ≈ 2*G_se_*, as *G_mc_* and *G_se_* become arbitrarily large, the τ = 2 aTAM is approximated arbitrarily well, and error rates go to zero—concomitantly, assembly speed goes to zero (Winfree 1998a). For ranges of *G_mc_* and *G_se_* compatible with current experimental conditions, assuming thermodynamic and kinetic parameters extrapolated from the literature of DNA duplex hybridization (Bloomfield et al. 2000), this model (Figure S1) predicts mismatch error rates between 0.1% and 1.0% (Figure 2B).

**Figure 3:**
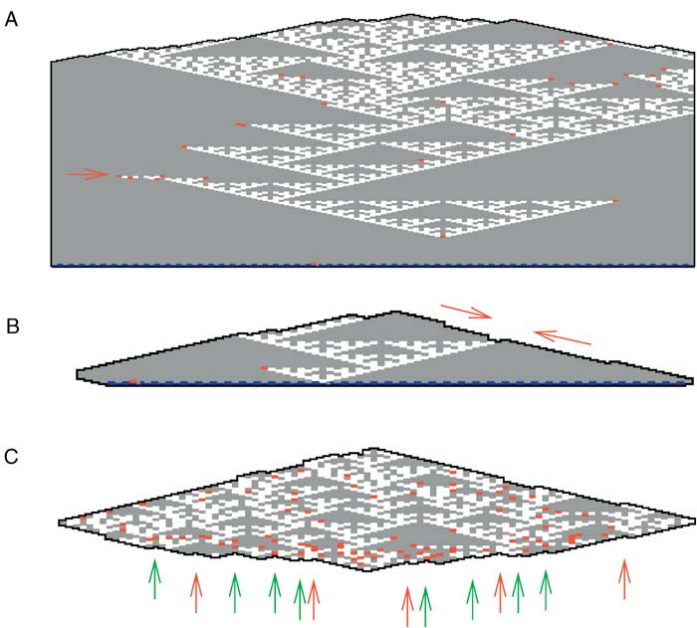
Simulations with Slow Border Growth and T-00 and T-11 at Doubled Concentrations (A) A common error pattern: termination of triangles at corners. (B) An observed mechanism leading to termination or sideways extension of triangles: preferential nucleation of T-00 on facets. (C) Forward and sideways growth is deterministic: at sites presenting two binding domains, there is always a unique tile that can form exactly two bonds. Backward growth is non-deterministic: at sites where both binding domains agree (e.g., green arrows), there are two possible tiles that can make two bonds (either {T-10, T-01}, or {T-00, T-11}). At sites where the available binding domains disagree (e.g., red arrows), there is no tile that can associate to form two bonds. Since only the output type of tiles are shown, it is impossible to tell from these figures which backward growth sites present agreeing or disagreeing binding domains.

The effects of non-idealities can also be explored in this model. For example, Figure 2C shows growth when the concentrations of the T-00 and T-11 tiles are twice that of the T-01 and T-10 tiles, and the nucleating structure grows slowly from special nucleating tiles rather than being preformed. Under this condition there is a preferential association of ‘0's on the facets of the growing crystal, causing characteristic errors that terminate Sierpinski triangles at corners and result in large all-zero patches (Figures 3A and S2). The mechanism responsible for these errors appears to be preferential nucleation of T-00 tiles on all-zero facets, due to their higher concentration (Figure S3). If nucleation occurs on an all-zero facet both above and below a ‘1' tile, correct growth from the ‘1' will be sandwiched between ‘0's and therefore further errors will be forced (Figure 3B). The further errors could be either (1) termination of the Sierpinski triangle by addition of a mismatched ‘0' tile at the corner site, or (2) sideways propagation creating a new small triangle by addition of a mismatched ‘1' tile on the facet below the corner site (arrow in Figure 3A). Thus, slight quantitative variations in the model parameters can lead to striking qualitative differences in the observed error morphologies, which are effectively never seen under growth conditions with equimolar tile concentrations or with preformed borders (see Figure S2).

The kTAM also provides insights into a second kind of error, the spontaneous 2D nucleation of untemplated crystals in the absence of the nucleating structure. For *G_mc_* ≈ 2*G_se_*, which corresponds to the melting temperature of the crystals, such untemplated nucleation is inhibited by a kinetic barrier—the existence of a critical nucleus size (Davey and Garside 2000) that decreases with increasing supersaturation. The growth rate of untemplated crystals also increases with supersaturation since their growth occurs by spontaneous 1D nucleation of a new layer of tiles on any of four facets. Via any single binding domain, there are always two tiles that can bind, so such nucleation must effectively invent a new bit of information. This bit may be propagated quickly forward or sideways (wherein tiles attach by one input and one output domain) to complete the layer without error according to the logic of XOR. Consequently, such crystals have none of the qualitative appearance of Sierpinski triangles even though they may contain no mismatched tiles (see Figure 2D). If *G_se_* is increased, corresponding to lowering the temperature, nucleation occurs more rapidly but errors are more frequent. Backward growth, in which tiles attach to a crystal by both of their output domains, is especially error-prone since every one of these associations involves the invention of information (Figure 3C). Whenever two backward-growing domains meet and disagree on the information that they have invented, growth can only proceed via an error. Under fast growth conditions, significantly below the melting temperature as in Figure 2E, this gives rise to higher error rates in the reverse growth direction. Near the melting temperature, however, this effect is insignificant. The most noticeable effect for untemplated crystals is due to the non-ideality discussed above (doubling the relative concentration of T-00 and T-11 tiles): the statistical preference for all-zero patches actually *increases* the frequency and size of perfect Sierpinski patterns (see Figure 2F).

These simulation studies suggest that all three difficulties (asynchronous association of tiles, mismatch errors, and untemplated nucleation) in principle can be controlled by slowing down the growth processes, making experimental investigations the appropriate next step.

### Design and Preparation of DNA Tiles

**Figure 4:**
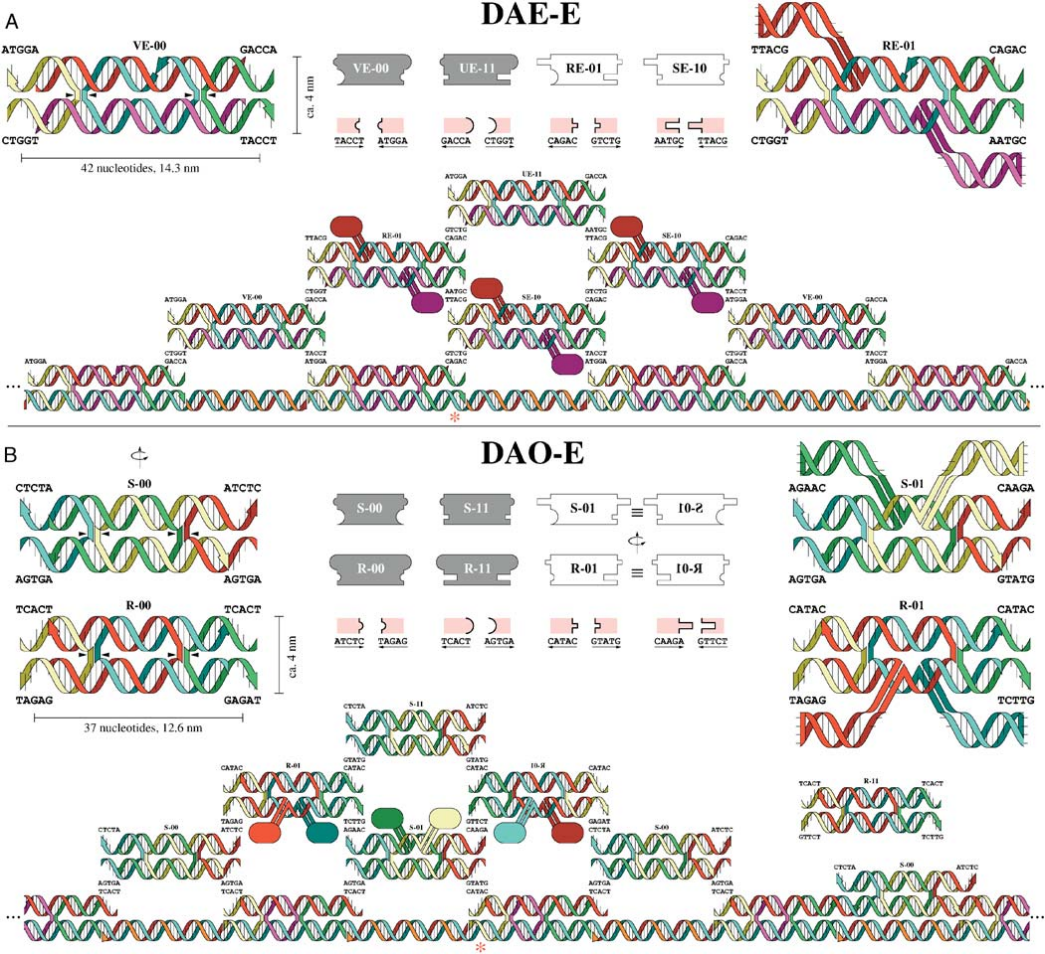
Molecular Schema (A) Top center: abstract versions of the four DAE-E Sierpinski rule tiles, VE-00, UE-11, RE-01, and SE-10, highlight their differences from the tiles in Figure 1. The arrangement of 3′ and 5′ ends on DAE-E tiles dictates that two distinct pairs of complementary binding domains must be used for each symbol ‘0' or ‘1,' denoted here by making complementary shapes large or small. Pink legends show the mapping of shape to sticky-end sequences. Top left: a molecular diagram for VE-00 shows how each DAE-E tile is comprised of five DNA strands; small arrows point to crossovers. Top right: a diagram for RE-01 shows how hairpins are attached to ‘1' tiles to provide AFM contrast; the exact orientation of these hairpins is unknown. Below: tiles are shown assembling on a nucleating structure. The nucleating strand for the input row (blue) is generated by assembly PCR and frequently reaches lengths of more than 3 μm (200 tiles). The nucleating strand contains subsequences onto which capping strands (orange) and input tile strands assemble to form an input tile outputting ‘0' at random intervals, the nucleating strand contains a subsequence (asterisk) for a different input tile that outputs a ‘1' on one side. (B) Top center: the six DAO-E Sierpinski rule tiles: S-00, R-00, S-11, R-11, S-01, and R-01. Top left and right: molecular diagrams highlight two notable features: (1) R-type tiles output only to S-type tiles, and vice-versa, as dictated by the 3′/5′ polarity of the molecules—again requiring two distinct pairs of binding domains per symbol. (2) The indicated rotational symmetry of the DAO-E molecules allows each molecule to serve in either of two orientations; no explicit S-10 or R-10 tiles are needed. An input tile outputting a single ‘1' sticky end is shown (asterisk). Sequences are given in Figures S4–S7.

Abstract Wang tiles are implemented as DNA tiles according to the scheme described earlier (Winfree et al. 1998a): each molecular Wang tile is a DNA double-crossover molecule (Fu and Seeman 1993) with four sticky-ends (5-base single-stranded overhangs) that serve as the programmable binding domains. We rendered the four Sierpinski rule tiles using two types of double-crossover molecule, known as DAE-E and DAO-E molecules (Winfree 1996), resulting in two independent molecular implementations (Figure 4, sequences are as given in Figures S4–S7). The DAE-E Sierpinski tile set (Figure 4A) consists of four molecular tiles, each composed of five strands whose sequences were designed to minimize the potential for forming alternative structures (Seeman 1990), as confirmed by non-denaturing gel electrophoresis (Figure S8).

Since untemplated crystals were not expected to produce recognizable Sierpinski triangles, it was necessary to create a proper nucleating structure to provide the initial input for the algorithmic self-assembly. Previous work using DNA tiles to self-assemble an initial boundary had proven to be difficult (Schulman et al. 2004), so in this work we took an alternative approach of using assembly PCR (Stemmer et al. 1995) to create a long single-stranded molecule which could serve as a scaffold (LaBean et al. 2000a; Yan et al. 2003a) for the assembly of a row of input tiles (Figures 4A and S9–S11). Because this nucleating strand serves as the bottom of these tiles, only four strands are needed to assemble the input tiles, and an additional capping strand is used to form a double-helix between input tiles on the nucleating strand. By doping the assembly PCR mix with a small fraction of the strands coding for an input tile outputting a single ‘1,' we ensure that each nucleating structure contains a few randomly located sites from which a Sierpinski triangle should grow.

The DAO-E Sierpinski tile set (Figure 4B) consists of six molecular tiles, due to peculiarities of the DAO-E motif. First, consideration of the 5′ and 3′ orientation of strands—particularly the fact that the sticky ends at the top and bottom of a DAO-E tile have opposite polarity—demands that each tile binds only to “upside-down” neighbors, resulting in layers of tiles with alternating orientation, which we refer to here as R-type and S-type tiles. Furthermore, the sugar–phosphate backbone of the DAO-E tiles has a dyad symmetry axis, implying that the R-01 and S-01 tiles each can play the roles of both the T-01 and T-10 tiles. Likewise, the R-00, R-11, S-00, and S-11 tiles can each bind in two orientations in a site where both inputs match.

In order for the nucleating structure for the DAO-E lattice to assemble onto a long PCR-generated nucleating strand, the tiles on the input row must be of the DAE-O variant. Further, we simplified the construction so that all nucleating strands contain the same repetitive sequence, but the input tile strands are doped with a fraction of strands containing a ‘1' sticky-end, and again the nucleating structure contains a few randomly located sites from which a Sierpinski triangle should grow.

### Self-Assembly of DNA Sierpinski Triangles

In principle, two approaches can be taken for initiating algorithmic self-assembly of DNA tiles. In the preformed tile approach, each tile is prepared separately by mixing a stoichiometric amount of each component strand in the hybridization buffer and then annealing from 90 °C to room temperature over the course of several hours. The nucleating structure is similarly prepared by annealing the nucleating strand with input tile and capping strands. Then the rule tiles and nucleating structure are mixed together at a temperature appropriate for crystal growth. In the bulk annealing approach, the nucleating strand, the capping and input tile strands, and the strands for all rule tiles are initially mixed together and then annealed. Since, at the concentrations we use, the tiles themselves have melting temperatures between roughly 60 °C and 70 °C while the crystals have a melting temperature within a few degrees of 40 °C (Figure S12), during annealing the tiles themselves will first form, and only later will the fully formed tiles assemble into crystals, presumably growing from the nucleating structure prior to overcoming the barrier to spontaneous nucleation. Both approaches work, but because of the convenience of the bulk annealing approach, all samples reported here were prepared using that method, with a final concentration of 0.2 μM each tile. After self-assembly in solution, samples are deposited onto mica and imaged by tapping mode atomic force microscopy (AFM).

**Figure 5:**
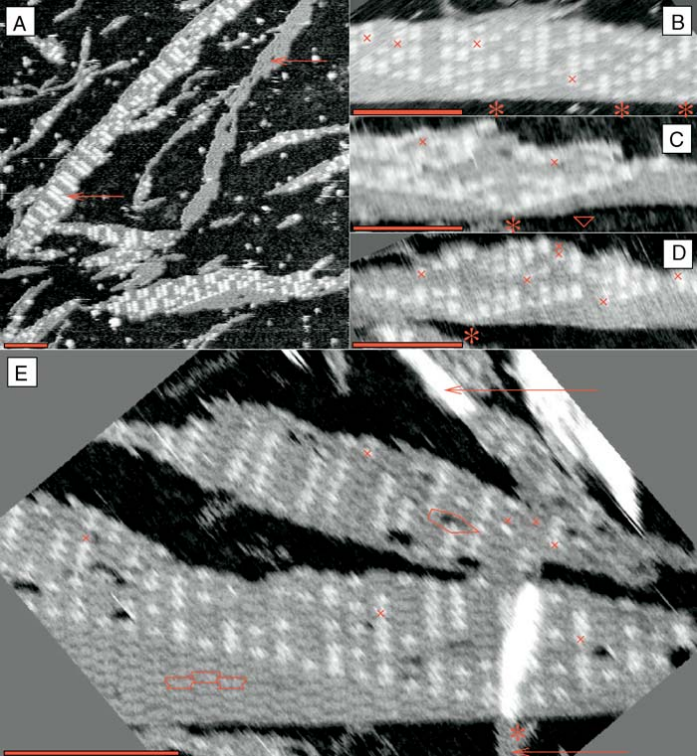
AFM Images of DAE-E Crystals (A) Several frequent morphologies that appear in most samples, including all-'0' (upper arrow) and ‘011011'-striped crystals (lower arrow). The all-'0' crystal may be a tube that opened upon adsorption to the mica. (B) A templated crystal. The identification of tiles in this crystal is given in Figure 1E. Crosses indicate mismatch errors. Asterisks indicate ‘1's on the nucleating strand. (C) A crystal containing 10 rows of error-free Sierpinski triangle. A red triangle marks a lattice defect in the input row. (D) Another Sierpinski triangle, better resolved. (E) A crystal containing a perfect 19 × 6 subregion. Individual tiles can be clearly seen; three tiles are outlined in the lower left. Unfortunately, this crystal landed atop a thin sliver of DNA (lower arrow), obscuring the central columns of the Sierpinski triangle. The upper arrow indicates a 4-tile wide tube, near the point where it opens. A pentagon marks a lattice dislocation. Scale bars are 100 nm.

Results for the DAE-E tile set are shown in Figures 5 and S13–S15. The majority of DNA crystals we observed were similar to those in Figure 5A: in addition to many small and indistinctly formed fragments, larger crystals are typically thin and long (up to several microns) with ‘1' tiles clearly visible. Crystals consisting exclusively of VE-00 tiles (upper arrow in Figure 5A) were particularly common; further investigation revealed that some (perhaps all) of these crystals formed as DNA tubes, and subsequently broke open and lay flat on the mica (see Figure S15; Rothemund et al. 2004). A ‘011011'-striped pattern (lower arrow in Figure 5A) was also quite common; it can be constructed from the RE-01, SE-10, and UE-11 tiles. Growth may have been biased to form ‘011011' patterns by the depletion of VE-00 tiles, a stoichiometric disproportionation of tiles, due to growth of tubes early during annealing. Crystals that clearly grew from the nucleating structure were also apparent; Figure 5B–5E show examples with particularly few errors. In several of these crystals, individual tiles could be identified and a compatible arrangement of abstract tiles (and thus errors) could be determined. Large error-free domains containing more than eight rows of perfect Sierpinski triangle were observed. In these examples, the mismatch error rate was about 2% over a wider selection of fragments, the error rate varied between 1% and 10%. We partly attribute this variation to changes in the physical conditions during annealing that result in a disproportionation of tiles. In addition to errors due to incorporation of the wrong tile, we also observed missing tiles and lattice dislocations. Frequently, as in Figure 5E, the identity of obscured or missing tiles was deduced from the neighboring tiles by assuming correct information propagation (the imperfection often being caused by sample preparation or by interaction with the AFM tip rather than by errors during assembly).

**Figure 6:**
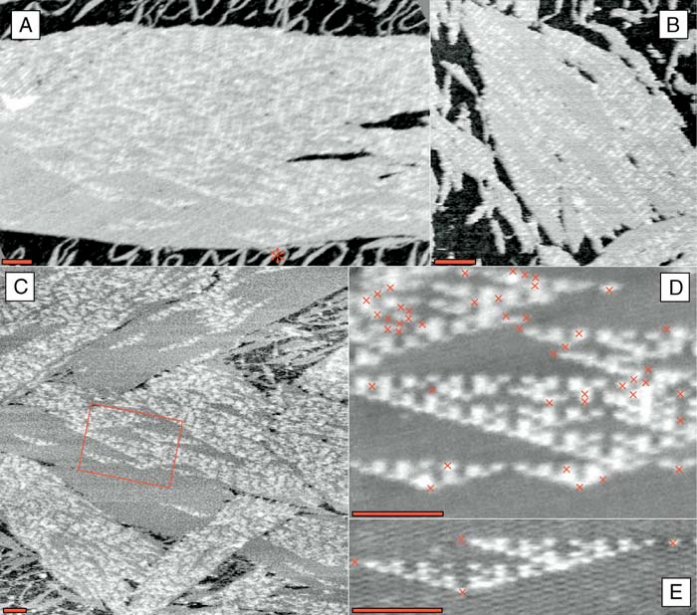
AFM Images of DAO-E Crystals (A) A large templated crystal in a 5-tile reaction (no R-11). A single ‘1' in the input row (asterisk) initiates a Sierpinski triangle, which subsequently devolves due to errors. Mismatch errors within ‘0' domains initiate isolated Sierpinski patterns terminated by additional errors at their corners. (B) A large untemplated fragment in a 5-tile reaction (no S-11). Large triangles of ‘0's can be seen. Crystals similar to this are also seen in samples lacking the nucleating structure. (C) Several large crystals in a 6-tile reaction, some with more zeros than ones, some with more ones than zeros. It is difficult to determine whether these crystals are templated or not. (D) An average of several scans of the boxed region from (C), containing roughly 1,000 tiles and 45 errors. (E) An average of several scans of a Sierpinski triangle that initiated by a single error in a sea of zeros and terminated by three further errors (a 1% error rate for the 400 tiles here). Red crosses in (D) and (E) indicate tiles that have been identified (by eye) to be incorrect with respect to the two tiles from which they receive their input. Scale bars are 100 nm.

Shown in Figures 6 and S16–S18, the DAO-E tiles also succeeded in producing recognizable Sierpinski triangles. However, the DAO-E tiles self-assembled into considerably larger sheets than the DAE-E tiles, presumably because of the DAO-E tiles' symmetries that result in cancellation of strain and thus encourage flat sheets. Templated crystals were observed that had grown more than 70 rows (Figure 6A). Because the R-11 tile does not appear in an error-free templated crystal, in some experiments (Figure 6A) we did not include this tile; however, we observed no qualitative difference between samples prepared with and without R-11. In these crystals we almost always observed subregions with a characteristic pattern of errors that coincidentally resulted in termination of Sierpinski triangles at their corners and tops, creating large patches of zeros. Even untemplated crystals (Figure 6B) contained recognizable subregions of the Sierpinski pattern. These features may be explained as follows: although the DAO-E tiles were mixed with equal quantities, the R-00, S-00, R-11, and S-11 tiles can bind to any permitted site in two orientations, thus making the experimental conditions analogous to the simulations of Figure 2C–2F wherein the concentration of the corresponding tiles is doubled; slow growth of the input row in the simulations may correspond to slow straightening out of the nucleating strand, which is initially a random coil (see Figure S17). Large crystals often have strikingly different tile distributions and error rates, as can be seen in the amalgamation of several large crystals shown in Figure 6C and Video S1. Again, this may be attributed to the disproportionation of tiles during annealing, or to sideways growth as the nucleating structure straightens out. Figure 6D–6E shows particularly clear examples of Sierpinski triangles, averaged from several scans of the same crystal. Attempts to optimize the reaction conditions to produce Sierpinski triangles with lower error rates did not yield dramatic improvements (Figure S18).

## Discussion

The self-assembly of DNA Sierpinski triangles demonstrates all four features necessary for Turing-universal computation by crystallization: formation of extended crystals, programmable interactions between DNA tiles determined by sticky-end sequences, selective associations of tiles enforced by the cooperative binding of more than one sticky end, and controlled nucleation of growth initiated by a template containing input information. This tiling approach could be used to implement other cellular automaton rules. Given a set *S* of possible states for the memory cells and an update function *f : S × S → S*, one can create a set of |*S*|^2^ tiles according to the scheme of Figure 1B, one tile for each possible input pair. The need for binding specificity limits the number of sticky-end sequences (and hence |*S*|) to about 20 for the DAO-E and DAE-E tile designs used here, but this is already sufficient to implement several known universal Turing machines and cellular automata (Lindgren and Nordahl 1990; Rogozhin 1996). A larger set of sticky-end sequences could be achieved by redesigning the DNA tile molecules to use longer sticky-ends, provided that the melting temperatures of tiles and crystals remain well separated. Thus, DNA crystallization is programmable and Turing-universal. Furthermore, for fabrication purposes, computation by self-assembly could be used to control the direction and extent of growth, thus allowing arbitrary shapes to be created efficiently (Soloveichik and Winfree 2005)—demonstrating that algorithmic self-assembly is not limited to the simulation of cellular automata or Turing machines.

The main obstacle currently limiting attempts to compute or fabricate using algorithmic self-assembly is the presence of several types of errors. We observed lattice dislocations, a structural error; untemplated tubes and untemplated crystals, an error in the control of nucleation; and mismatched tiles, an error in the growth process. Accurate quantitative models of algorithmic self-assembly will be valuable for developing methods to control and reduce such errors. The kTAM simulations described here, while qualitatively insightful, predict mismatch error rates an order of magnitude smaller than those observed—motivating experimental measurements of errors and refinement of the model. Although it may be possible to reduce the error rates by carefully controlling the assembly conditions, a more promising route is the creation of fault-tolerant tile sets that perform the same logic (Winfree and Bekbolatov 2004; Chen and Goel 2005; Reif et al. 2005; Schulman and Winfree 2005). For the same assembly conditions, and thus roughly the same growth rate, the kTAM predicts that these tile sets can reduce the mismatch error rates by many orders of magnitude—a conclusion likely to hold in spite of inaccuracies in the model.

Self-assembly has been touted as a possible successor to photolithography, a basis for nanotechnology and a route to complexity in chemistry (Whitesides et al. 1991). Algorithmic self-assembly—whether using DNA tiles as demonstrated here or using appropriately designed small molecules, proteins, or even macroscopic tiles (Bowden et al. 1997; Rothemund 2000)—extends the range of structures accessible by bottom-up fabrication techniques. For example, an abstract tile set that enumerates binary numbers—a binary counter—uses just four tiles, yet it can be used to define the size of self-assembled structures (Rothemund and Winfree 2000), thus addressing the synthetic chemistry challenge of creating monodisperse particles with programmable size. Furthermore, attachment of suitable logic gates to ‘0' and ‘1' tiles would yield a demultiplexer for a RAM circuit. This and other interesting digital circuits (Cook et al. 2004) might be created by using algorithmic crystals as templates for further chemical processing (Braun et al. 1998; Yan et al. 2003b).

The Turing-universality of self-assembly allows theoretical insights from computer science to be applied to self-assembly. For example, many questions phrased using the aTAM—such as “Will a certain tile type, say tile type #5, ever be incorporated into the assembly?” or “Will the final assembled shape have 4-fold symmetry?”—are formally undecidable as a consequence of the undecidability of the halting problem (Turing 1936; Adleman et al. 2002). This suggests that there exists no generally applicable method for predicting the behavior or properties of crystals. A concrete instance of this dilemma is whether quasicrystals' 5-fold symmetry and aperiodicity could arise from self-assembly. Crystallographers have argued that, if so, definitions of order based on X-ray diffraction must be modified to include the new structures (Senechal 1995). The growth of Sierpinski triangles, demonstrated here, shows unequivocally that self-assembly can create aperiodic structures based on local rules. Furthermore, traditional methods of measuring order, such as X-ray diffraction, will not recognize order that exists in certain algorithmic crystals. For example, an algorithmic crystal simulating a pseudo-random number generator (Wolfram 1986; Jen 1990; Knuth 1997) would appear disordered, yet each molecule would be precisely and deterministically positioned. Thus, the growth of algorithmic crystals motivates the use of algorithmic definitions of order (Kolmogorov 1965; Levin 1984; Bennett 1995) that generalize crystallography (Mackay 1975).

Finally, we ask whether the study of algorithmic self-assembly might further our understanding of biological self-assembly. Algorithmic crystals composed of simple sugar-based tiles have appeared in science fiction as a form of life (Egan 1995); indeed, the simplicity and versatility of crystalline self-assembly suggests that templating, as a basis for simple organisms (Penrose and Penrose 1957; Cairns-Smith 1971), may be more natural than previously supposed. However, examination of self-assembly in modern organisms reveals many mechanisms beyond those considered here, including conformational changes, dissipative mechanisms such as ATP hydrolysis, and interactions with genetic regulatory networks—themselves biochemical information processors. The development of a theory of molecular algorithms that encompasses these additional mechanisms, if successful, will deepen our understanding of the complex processes found in nature, their fundamental limits, and their remarkable potential.

## Materials and Methods

### 

#### kTAM simulations

Simulations described in this paper were performed with the xgrow program, written by Erik Winfree and available, along with tile sets used here, from http://www.dna.caltech.edu/SupplementaryMaterial.

The xgrow program simulates the kTAM for a set of square Wang tiles (see Figure S1), beginning with a single seed tile. The tile set used here consists of the four Sierpinski rule tiles T-00, T-11, T-01, and T-10, augmented by three border tiles B-0, B-1, and B-B, the latter being used as the seed tile. To simulate the presence of a nucleating structure, the binding domain that joins border tiles is considered to be twice as strong as the other bonds—that is, it counts as two bonds in the sum *b* that determines off-rates *r_r,b_* =*ke* ^−*bG_se_*^ . The border row grows—simulating the long nucleating structure becoming straight—by association of border tiles at the rate *r_f_* = *k*[*tile*] = *kS_i_e* ^−*G_mc_*^ , where *S_i_* is the stoichiometry of border tile *i* relative to the concentration of the four Sierpinski rule tiles. Since we have no knowledge of how quickly DNA nucleating structures straighten in our experiments, we considered two cases: (1) A rigid or quickly straightening nucleating structure was simulated by setting *S_i_* = 4, so that near the crystal melting temperature where *G_mc_* ≈ 2*G_se_*, the border growth is strongly favorable. This was used for Figure 2B, where the seed tile stoichiometry was also set to zero, so that exactly one seed tile would be incorporated into the nucleating structure. (2) A floppy and slowly straightening nucleating structure was simulated by setting *S_i_* = 0.25 for the border tiles; in this case, near the melting temperature border growth requires stabilization by growth of rule tiles, resulting in faceted crystals. In combination with doubled concentrations of T-00 and T-11 (*S_i_* = 2), this case was used for Figure 2C, where additionally the seed tile stoichiometry was set to 0.01 so that roughly 1% of border tiles output a ‘1,' in rough agreement with the observed fraction of ‘1's within the DNA nucleating structures in our DAO-E experiments.

The strong effect of these variations may be seen in Figure S2. Slow border growth significantly increases the mismatch error rate, resulting in the information contained in the border being lost in a few layers. The primary effect of doubled T-00 and T-11 concentrations is to increase the predominance of all-zero patches in the resulting crystal; not only are all-zero patches typically larger, but all-zero information in the border is propagated more reliably. Additionally, under these conditions spontaneous nucleation almost exclusively involves an initial all-zero nucleus.

Simulations confirm the preferential nucleation of T-00 tiles on all-zero facets when T-00 and T-11 concentrations are doubled (Figure S3). In contrast, preferential nucleation on facets is *not* seen for the T-11 tile, despite its increased concentration. This is because, regardless of what information is presented on the facet, there is no way to create a layer containing more than 50% T-11 tiles and no mismatches; T-01 or T-10 tiles must intervene. Thus the nucleation rate is substantially reduced, relative to T-00 nucleation on all-zero facets. This can be assessed in simulations by measuring the probability, *p(L),* that a T-00 tile will be found after *L* layers of growth from a facet. Simulations with parameters similar to Figure 2C (doubled T-00 and T-11 concentrations) show that *p(L)* ≈ 0.66*e^−L/27^* + 0.34 for all-zero facets, indicating strongly preferential nucleation, but for all other facets *p(L)* relaxes quickly to the asymptotic distribution. Simulations with parameters similar to Figure 2B (normal T-00 and T-11 concentrations) show no preferential nucleation, as *p(L)* relaxes to the asymptotic distribution immediately for every facet type investigated.

#### DNA sequence design

Design of DNA Wang tiles occurs in three steps. First, the tile and lattice geometry must be determined. Here, the sizes (number of base-pairs) of each double-helical domain and sticky end, and other structural adornments such as contrast hairpins, are decided. These decisions impact the stability of each tile molecule, as the natural geometry of the DNA double-helix (10.5 bp for a full turn of B-form DNA) (Wang 1979; Rhodes and Klug 1980) constrains, for example, the separation between crossover points to be an integral number of half-turns. For the double-crossover motif used here (Fu and Seeman 1993), the acronym DAE-E refers to some of these choices at the structural level: double-crossover; antiparallel orientation of non-crossover strands at each junction; even number of half-turns (21 bp) between crossover points within each molecules; and even number of half-turns (21 bp) between nearest crossover points in two molecules joined by sticky ends. DAO-E refers to a similar set of choices, except that an odd number of half-turns (16 bp) separates the crossover points within each molecule. Where hairpin sequences were inserted for AFM contrast, we included two unpaired Ts at the bulged three-arm junctions, which has been shown to encourage stacking in the original helix domain (Ouporov and Leontis 1995) without significantly affecting the rigidity of the molecule (Li et al. 1996).

At the second level, specific sequences must be chosen. The issue here is that we wish to prevent undesired associations between strands that might inhibit formation of the correct molecular structure. We used the heuristic principle of sequence symmetry minimization (Seeman 1982, 1990) to minimize the length and number of unintentional Watson–Crick complementary subsequences among all strands in each system (DAO-E and DAE-E). Violations that occurred within a single strand were weighed more heavily than violations between two strands; similarly, violations between strands in the same tile were weighed more heavily than violations between strands in different molecules. A simple adaptive walk algorithm was found to be effective in minimizing the violations and arriving at acceptable sequences. Sticky-end sequences were chosen with particular care to minimize the possibility of erroneous hybridization.

The third level of design concerns variations. We conceptualize DNA Wang tiles as consisting of three modules: the sticky ends, the core helical regions, and adornments such as the hairpin structures that provide contrast for AFM imaging. A given double-crossover core can be given different sticky ends (reprogrammed) by replacing just one or two strands, thus allowing reuse of core designs to implement different tile sets. In our experience, the structural and thermodynamic stability of a given core is not significantly affected by changes in the sticky-end sequences. Similarly, using additional strands, a given core can be used with or without the hairpin adornments, which can be inserted at various locations. Although the hairpin adornments can affect the integrity of a DNA tile (e.g., strand dimers or other high molecular weight species), we have seldom found the undesired products to exceed 20% of the material.

The core sequences for R-00 and S-00 are identical to the A and B tiles from a previous study (Winfree et al. 1998a). We usually give tiles names that indicate their core, sticky ends, and adornments. However, in the main text of this paper we have dispensed with the indication of these variations for clarity. For example, R-01 would more properly be called R-01n-23JC; S-01 called S-01-23JC; RE-01 called RE-01-15J; and SE-10 called SE-10-15J to specify which component strands have hairpins, and where those hairpins are. (The shorter names properly refer to unadorned tiles.)

#### DNA tile preparation and gel electrophoresis

All oligonucleotides were synthesized by standard methods (Integrated DNA Technologies), PAGE purified, and quantitated by UV absorption at 260 nm in H_2_O (purified by a Milli-Q system, Millipore, Bedford, Massachusetts, United States) based on extinction coefficients estimated using a nearest-neighbor model (Bloomfield et al. 2000). DNA tiles were prepared by mixing stoichiometric quantities of each component strand in a TAE/Mg^2+^ buffer, as described in Winfree et al. (1998a). Proper formation of each of the ten DAE-E and DAO-E tile cores was confirmed in non-denaturing PAGE (10%–15% 19:1 bis:acrylamide, 3–5 h at 15 V/cm and 4C, 2 pmol complex/lane, Sybr Gold [Molecular Probes, Eugene, Oregon, United States] stained for 20–30 min, excited at 488 nm, imaged with 530 bandpass filter on a Bio-Rad [Hercules, California, United States] Molecular Imager FX Pro Plus) by observing a single major band (see Figure S8; typically between 5% and 20% of the total material appears in bands identified as partial products, such as incomplete tiles with missing strands). We redesigned the core sequences for one tile (R01) that initially did not form clean gel bands; the new tile (R01n) was used exclusively in this study. Most notably in DAE-E tiles, some lanes containing subsets of a tile's component strands showed ill-formed or heavy species such as dimers, but these difficulties were not pronounced in lanes containing the complete tile. Formation gels also allow us to estimate the relative accuracy of our concentration measurements: mismatches in stoichiometry would result in excess single-stranded or partial complexes. Concentrations appear to be accurate to **±**10%. This suggests that purification of tile complexes could result in cleaner self-assembly reactions and lower error rates.

#### Synthesis of the nucleating strand

The single-stranded nucleating strands were synthesized using a procedure based on Stemmer's assembly PCR (Stemmer et al. 1995). In assembly PCR, a long, repetitive, double-stranded product is generated by performing PCR on a set of splints, primer-like short (typically 40 nt) oligos that are subsequences of the desired repeat sequence as shown in Figure S9. To generate the single-stranded product needed for subsequent assembly of tiles on the nucleating strand, asymmetric PCR with primers for just one of the two complementary product strands could be used, in principle. In practice, we have found that such reactions result in more double-stranded product and little or no single-stranded product—probably because the repetitive nature of the assembly PCR product means that every 3′ end, including those of the undesired strand, may act as a primer. Thus we designed the long covalent strand of our nucleating structures to contain exclusively As, Cs, and Ts and generated single-stranded nucleating strands from the output of an assembly PCR by performing synthesis using a reaction mixture containing just dATP, dCTP, and dTTP. Although predominantly single-stranded, the output of this reaction has both single- and double-stranded material in it. We do not purify the single strands and thus double-stranded material persists in our experiments (Figure S17). The splint strands for generating the DAE-E nucleating strands and the DAO-E nucleating strands are given in Figure S10. Note that in order to have 20 base overlaps, some splint strands complement the same central three base sections of complementary splints.

Assembly reactions for DAO-E and DAE-E nucleating strands were designed using slightly different principles. The improved design used for DAO-E nucleating strands is simpler: A single periodic sequence is generated. The fraction of ‘1' sites is determined by the stoichiometry of input tile strands used in subsequent self-assembly reactions—strands A4SV and A4-S00 both assemble in the input tile in the same place, but one carries a ‘1' sticky end while the other carries a ‘0' sticky end. The approach used for DAE-E nucleating strands is more complex but more powerful for generating non-trivial input patterns. By having multiple splints that can overlap a given sequence, the assembly can be directed to non-deterministically choose one of several ways to extend a sequence. Thus, assembly PCR can be used to generate any regular language (Winfree 1998b). In this work, we used a combination of splint strands that generates substrings of the language (NRE NUE**^+^**)*. The fraction of NRE subsequences is controlled by the amount of SplintNREUE2 and SplintNUERE2, which mediate the transitions into and out of the NRE sequence. (Here, we used these splints at one-fifth the concentration of other splints.) The NRE input tile outputs ‘0' and ‘1,' while the NUE input tile outputs ‘0' and ‘0'. To generate a different language, or a different distribution of sequences in the same language, a new assembly PCR must be run. (The simpler design approach used for DAO-E could also be used for DAE-E nucleating structures.)

For both methods, the PCR protocol has four stages, the first three for assembly PCR and the last to generate single-stranded material. PCR was performed in a Stratagene (La Jolla, California, United States) MX 4000 real-time PCR instrument using a Perkin-Elmer (Torrance, California, United States) GeneAMP XL kit that uses rTth polymerase. In stage 1, a 20 μl reaction mixture containing 1 pmol total of splints (of which there are *N* types) is prepared without polymerase (Mix A, per 20 μl: 1 μl of 1 μM mixed splints, 1/*N* μM each; 1.6 μl of 10 mM dNTPs, 2.5 mM each; 1 μl of 25 mM magnesium acetate, 6 μl of 3.3X GeneAMP XL PCR buffer; 10 μl water). To avoid mispriming events, the splints are annealed in the reaction mixture at 37 °C for 5 min. The polymerase (0.4 μl) is added and the reaction is subjected to an initial 72 °C extension step, followed by 40 cycles (94 °C for 15 s, 40 °C for 30 s, 72 °C for 10 s + 1 s/cycle; about 2 h). In stage 2, 40 μl of new PCR Mix B (Mix A minus the splints, plus 0.4 μl of polymerase, water adjusted to 20 μl), is added to the first reaction volume and the reaction cycled for an additional 25 cycles (94 °C for 15 s, 40 °C for 30 s, 72 °C for 45 s + 1 s/cycle; about 1.5 h). In stage 3, the 60 μl reaction volume is split into three 20 μl volumes, an additional 40 μl of Mix B is added to each and an additional 20 cycles (94 °C for 15 s, 40 °C for 30 s, 72 °C for 70 s + 1 s/cycle; about 1.3 h) are performed. At this point, long double-stranded product should be formed. (We have observed that such products remain in the well of an agarose gel long after a 20 kb marker has entered the gel.) Also the dNTPs in the mixture are presumably nearly exhausted—specifically there is little dGTP left. (Any remaining dGTP will be used up early in stage 4.) In stage 4, to create single-stranded nucleating strands, 5 μl of the stage 3 product are mixed with 55 μl of fresh PCR mixture (Mix B with 1.6 μl of a mixture containing 2.5 mM each dATP, dCTP, and dTTP, rather than all four dNTPs) for additional 60 cycles of the stage 3 program (94 °C for 15 s, 40 °C for 30 s, 72 °C for 70 s + 1 s/cycle). While addition of asymmetric primers at this stage might yield more single-stranded product, a satisfactory yield of single-stranded product results without doing so. After the final PCR, the reaction mixture is extracted with phenol:chloroform:isoamyl alcohol (Sigma, St. Louis, Missouri, United States), ethanol precipitated, and resuspended in purified water; the yield was estimated by UV absorbance. Typically, three 60 μl tubes of stage 4 product were pooled in a single recovery step and DNA was resuspended in 200 μl of water. Absorbance measurements of freshly resuspended material appeared unstable, perhaps because clumps of nucleating material scatter light. Long single-stranded DNAs may be prone to hydrolysis in water or strand-breakage upon freeze–thaw. However, after storage in water at 4 °C for a year, the nucleating structure still works well (as in Figure S18).

To check whether the output of stage 4 is suitable as a nucleating strand for self-assembly, one can estimate the binding capacity of the nucleating strand material. Figure S11 shows such a gel (non-denaturing PAGE, 5% 19:1 bis:acrylamide, 1 h, 150 V) for the DAO-E nucleating strand, examining how much of the fluorescently labeled Cy3-cpBr1 can be bound. We observe several things. First, stage 3 double-stranded material does not bind Cy3-cpBr1 well, as expected. Second, stage 4 material does bind Cy3-cpBr1 well, quantitatively absorbing the full amount (1 μl) added. Third, stage 4 material cannot absorb 2 μl of Cy3-cpBr1, giving us an estimate of the binding capacity of the nucleating strands. This is important for determining how much of the input tile strands must be added to ensure that a tile assembles onto nearly every site on the nucleating strand. Fourth, the presence of Sybr Green I during PCR does not appear to affect the quality of double or single-stranded material generated.

#### UV melts of tiles and crystals

Melting temperatures for tiles and crystals were estimated based on UV^260^ melts of S-00 and R-00-23J and a mixture of both tiles (Figure S12). These tiles, also used in Winfree et al. (1998a), are identical to the R-00 and S-00 tiles for the DAO-E Sierpinski system, but with hairpins added to the R-00 tile. Individual tiles were at 0.4 μM of each component strand in TAE/Mg^2+^. The mixture of R-00-23J and S-00, which forms crystals when annealed slowly, contained each strand at 0.2μM. Melts were performed on an Aviv model 14NT-UV-VIS spectrophotometer (Aviv Instruments/Protein Solutions, Lakewood, NJ, United States), and began with preannealed samples at 15 °C, increasing to 80 °C over the course of several hours. Single-tile melts were superimposable with the reanneal from 80 °C back to 15 °C, indicating that equilibrium values were measured. Raw absorbance values were normalized. Whereas S-00 has a sharp melting transition (also seen for most other tiles lacking hairpins) near 65 °C, the R-00-23J tile has a somewhat more gradual transition, which we attribute to the presence of the hairpin. Above 40 °C, the absorbance of the mixture equals the average absorbance of the individual tiles, indicating that crystals have completely melted by that point. Prior to the crystal melting transition between 36 °C and 40 °C, there is significant noise in the measurement, presumably due to light scattering.

We have not performed UV^260^ melts of all tiles; however, several other DAO-E and DAE-E tiles have similar transitions between 50 °C and 70 °C. Therefore we assume that the templated and untemplated Sierpinski crystals also melt at approximately 40 °C and that at that temperature, the DNA tiles are reasonably well formed.

#### Self-assembly reactions

Self-assembly was performed by bulk annealing of all relevant rule tile, input tile, capping, and nucleating strands in a (typically) 50 μl volume of 1× TAE/Mg^2+^ buffer (40 mM Tris–acetate [pH 8.0], 2 mM EDTA, 12.5 mM Mg^2+^), annealing from 90 °C to 20 °C at a rate of 1 °C/min (taking about 1 h). Longer annealing schedules (e.g., 1 °C/min from 90 °C to 50 °C followed by 1 °C/30 min in the critical region from 50 °C to 20 °C, a total of about 15 h) did not seem to decrease the error rate or the number of untemplated tubes or crystals.

DAO-E reactions contained nucleating strands sufficient to bind 0.004 μM of input tile (as estimated from binding capacity gels), 0.2 μM of each capping and input tile strand (A1S, A2, A3-nick, A4-S00, cpBr1, and 1/100 as much A4SV), and 0.2 μM of each rule tile strand (for each of the five or six tiles used). An excess of input tile strands was used to ensure complete coverage of the nucleating strand. The excess partial input tiles appeared not to significantly interfere with the self-assembly of algorithmic crystals.

DAE-E reactions contained nucleating strands sufficient to bind 0.002–0.008 μM of input tile (as inferred from the estimated yield of the PCR), 0.2 μM of each capping and input tile strand (NRE1 to NRE4, NUE1 to NUE4, CapNRERE and CapNUERE), and 0.2 μM of each tile strand (for each of the four tiles used). Again, an excess of input tile strands was used to ensure complete coverage of the nucleating strand.

#### AFM imaging

AFM imaging was performed in tapping mode under TAE/Mg^2+^ buffer on a Digital Instruments Nanoscope III (Veeco Metrology, Woodbury, New York, United States) equipped with a nano-Analytics Q-control III (Asylum Research, Santa Barbara, California, United States) and a vertical engage J-scanner, using the roughly 9.4 kHz resonance of the narrow 100 μM, 0.38 N/m force constant cantilever of an NP-S oxide-sharpened silicon nitride tip (Veeco Metrology). After self-assembly is complete, samples were prepared for AFM imaging by deposition of 5 μl onto a freshly cleaved mica surface (Ted Pella) attached by hot melt glue to a 15 mm metal puck; an additional 30 μl of buffer was added to both sample and cantilever (mounted in the standard tapping mode fluid cell) before the sample and fluid cell were positioned in the AFM head. The tapping amplitude setpoint, after engage, was typically 0.2–0.4 V, the drive amplitude was typically 100–150 mV, scan rates ranged from 2 to 5 Hz. Individual tiles are most clearly resolved for low amplitude setpoint and high drive amplitude values. However, under such conditions, the greatest damage is done to the sample and the hairpin labels are less distinct, sometimes disappearing entirely. Thus, to prevent damage to samples, amplitude setpoint was maximized and/or drive amplitude minimized subject to the constraint that tiles and their hairpin labels be visible.

After acquisition, most images were flattened by subtracting a low-order polynomial from each scan line, or by adjusting each scan line to match intensity histograms. For some images (see Figures 6D–6E and S18, bottom), multiple scans were aligned using hand-picked fiducial marks and averaged in Matlab (The Mathworks, Inc., Natick, Massachusetts, United States).

## Supporting Information

Figure S1 Representations and Tile Sets Used in Simulations (57 KB PDF).

Figure S2 Behavior of Simulated Crystal Growth (160 KB PDF).

Figure S3 Simulations of Growth on Large Facets (126 KB PDF).

Figure S4 DAE-E Strand Sequences (16 KB PDF).

Figure S5 DAE-E Tile Diagrams (21 KB PDF).

Figure S6 DAO-E Strand Sequences (16 KB PDF).

Figure S7 DAO-E Tile Diagrams (22 KB PDF).

Figure S8 Formation Gels for Representative DAO-E and DAE-E Tiles (244 KB PDF).

Figure S9 Using Assembly PCR to Generate Long, Repetitive, Single-Stranded DNA (22 KB PDF).

Figure S10 Assembly PCR Scheme for DAE-E and DAO-E Nucleating Strands (25 KB PDF).

Figure S11 Binding Capacity Gel for Determining Nucleating Strand Stoichiometry (53 KB PDF).

Figure S12 Melts of R-00-23J and S-00 and Their Mixture (33 KB PDF).

Figure S13 AFM Images Showing the Context and Distribution of DAE-E Crystals (234 KB PDF).

Figure S14 AFM Images Showing the Context and Distribution of DAE-E Crystals (203 KB PDF).

Figure S15 AFM Images of DAE-E Crystals and Tubes (226 KB PDF).

Figure S16 AFM Images Showing the Context and Distribution of DAO-E Crystals (256 KB PDF).

Figure S17 AFM Images of Boundary Assemblies and Untemplated DAO-E Crystals (234 KB PDF).

Figure S18 AFM Images of DAO-E Crystals Grown under Constant-Temperature, Near-Constant Concentration Conditions (146 KB PDF).

Figure S19. Compiled Figures S1–S18 This file contains Figures S1–S18 and their captions in a single file for convenient printing (1.7 MB PDF).

Video S1 Composite of 64 AFM Images Taken Sequentially at Scales from 24 μm to 24 nm Each frame is an average of three raw images. At the center is an amalgamation of many individual algorithmic crystals, each with its own characteristic pattern of tiles (e.g., mostly zero, bearing small triangles, or apparently random). While no large undamaged Sierpinski triangles were seen in this series of images, in some frames it is possible to see both double-helices within the tiles, as well as the major and minor grooves within the helices.
(17.8 MB MPG).

## Abbreviations

AFM: atomic force microscopy
aTAM: abstract Tile Assembly Model
1D: one-dimensional
2D: two-dimensional
DNA: deoxyribonucleic acid
DAE-E: double-crossover
DAO-E: double-crossover
kTAM: kinetic Tile Assembly Model
PCR: polymerase chain reaction
RAM: random-access memory
XOR: exclusive or

## References

[pbio-0020424-Adleman1] Adleman LM (1994). Molecular computation of solutions to combinatorial problems. Science.

[pbio-0020424-Adleman2] Adleman LM, Cheng Q, Goel A, Huang MDA (2001). Running time and program size for self-assembled squares. Symposium on Theory of Computing (STOC).

[pbio-0020424-Adleman3] Adleman L, Kari J, Kari L, Reishus D (2002). On the decidability of self-assembly of infinite ribbons. Symposium on Foundations of Computer Science (FOCS).

[pbio-0020424-Bennett1] Bennett CH, Herken R (1995). Logical depth and physical complexity. The universal Turing machine: A half-century survey.

[pbio-0020424-Bloomfield1] Bloomfield VA, Crothers DM, Tinoco I (2000). Nucleic acids: Structures, properties, and functions.

[pbio-0020424-Bondarenko1] Bondarenko BA (1993). Generalized Pascal triangles and pyramids, their fractals, graphs and applications. Bollinger RC, translator and editor.

[pbio-0020424-Bowden1] Bowden N, Terfort A, Carbeck J, Whitesides G (1997). Self-assembly of mesoscale objects into ordered two-dimensional arrays. Science.

[pbio-0020424-Braun1] Braun E, Eichen Y, Sivan U, Ben-Yoseph G (1998). DNA-templated assembly and electrode attachment of a conducting silver wire. Nature.

[pbio-0020424-CairnsSmith1] Cairns-Smith AG (1971). The life puzzle: On crystals and organisms and on the possibility of a crystal as an ancestor.

[pbio-0020424-Chen1] Chen HL, Goel A (2005). Error free self-assembly using error prone tiles. DNA computing.

[pbio-0020424-Cook1] Cook M, Rothemund PWK, Winfree E, Chen J, Reif J (2004). Self-assembled circuit patterns. DNA computing.

[pbio-0020424-Davey1] Davey R, Garside J (2000). From molecules to crystallizers.

[pbio-0020424-Egan1] Egan G, Bear G, Greenberg MH (1995). Wang's carpets. New legends.

[pbio-0020424-Fu1] Fu TJ, Seeman NC (1993). DNA double-crossover molecules. Biochemistry.

[pbio-0020424-Jen1] Jen E (1990). Aperiodicity in one-dimensional cellular automata. Physica D.

[pbio-0020424-Knuth1] Knuth DE (1997). The art of computer programming, volume 2: Seminumerical algorithms, 3rd ed.

[pbio-0020424-Kolmogorov1] Kolmogorov AN (1965). Three approaches to the quantitative definition of information. Problems Inform Transmission.

[pbio-0020424-LaBean1] LaBean TH, Winfree E, Reif JH, Winfree E, Gifford DK (2000a). Experimental progress in computational by self-assembly of DNA tilings. DNA-based computers V.

[pbio-0020424-LaBean2] LaBean TH, Yan H, Kopatsch J, Liu F, Winfree E (2000b). Construction, analysis, ligation, and self-assembly of DNA triple crossover complexes. J Am Chem Soc.

[pbio-0020424-Levin1] Levin L (1984). Randomness conservation inequalities: Information and independence in mathematical theories. Inform Control.

[pbio-0020424-Li1] Li X, Yang X, Qi J, Seeman NC (1996). Antiparallel DNA double crossover molecules as components for nanoconstruction. J Am Chem Soc.

[pbio-0020424-Lindgren1] Lindgren K, Nordahl M (1990). Universal computation in simple one-dimensional cellular automata. Complex Sys.

[pbio-0020424-Mackay1] Mackay A (1975). Generalised crystallography. Izvj Jugosl Centr Krist Zagreb.

[pbio-0020424-Mao1] Mao C, Sun W, Seeman NC (1999). Designed two-dimensional DNA Holliday junction arrays visualized by atomic force microscopy. J Am Chem Soc.

[pbio-0020424-Mao2] Mao C, LaBean TH, Reif JH, Seeman NC (2000). Logical computation using algorithmic self-assembly of DNA triple-crossover molecules. Nature.

[pbio-0020424-Ouporov1] Ouporov IV, Leontis NB (1995). Refinement of the solution structure of a branched DNA three-way junction. Biophys J.

[pbio-0020424-Penrose1] Penrose LS, Penrose R (1957). A self-reproducing analogue. Nature.

[pbio-0020424-Radin1] Radin C (1985). Tiling, periodicity, and crystals. J Math Phys.

[pbio-0020424-Reif1] Reif J, Rubin H, Wood DH (1999). Local parallel biomolecular computing. DNA-based computers III.

[pbio-0020424-Reif2] Reif JH, Sahu S, Yin P (2005). Compact error-resilient computational DNA tiling assemblies. DNA computing.

[pbio-0020424-Rhodes1] Rhodes D, Klug A (1980). Helical periodicity of DNA determined by enzyme digestion. Nature.

[pbio-0020424-Rogozhin1] Rogozhin Y (1996). Small universal Turing machines. Theor Comput Sci.

[pbio-0020424-Rothemund1] Rothemund PWK (2000). Using lateral capillary forces to compute by self-assembly. Proc Natl Acad Sci U S A.

[pbio-0020424-Rothemund2] Rothemund PWK, Winfree E (2000). The program-size complexity of self-assembled squares. Symposium on Theory of Computing (STOC).

[pbio-0020424-Rothemund3] Rothemund PWK, Ekani-Nkodo A, Papadakis N, Kumar A, Fygenson DK (2004). Design and characterization of programmable DNA nanotubes. J Am Chem Soc.

[pbio-0020424-Schulman1] Schulman R, Winfree E (2005). Controlling nucleation rates in algorithmic self-assembly. DNA computing.

[pbio-0020424-Schulman2] Schulman R, Lee S, Papadakis N, Winfree E, Chen J, Reif J (2004). One dimensional boundaries for DNA tile assembly. DNA computing 9.

[pbio-0020424-Seeman1] Seeman NC (1982). Nucleic-acid junctions and lattices. J Theor Biol.

[pbio-0020424-Seeman2] Seeman NC (1990). De novo design of sequences for nucleic acid structural engineering. J Biomol Struct Dyn.

[pbio-0020424-Seeman3] Seeman NC (2003). Biochemistry and structural DNA nanotechnology: An evolving symbiotic relationship. Biochemistry.

[pbio-0020424-Senechal1] Senechal M (1995). Quasicrystals and geometry.

[pbio-0020424-Soloveichik1] Soloveichik D, Winfree E (2005). Complexity of self-assembled scale-invariant shapes. DNA computing.

[pbio-0020424-Stemmer1] Stemmer WPC, Crameri A, Ha KD, Brennan TM, Heyneker HL (1995). Single-step assembly of a gene and entire plasmid from large numbers of oligodeoxyribonucleotides. Gene.

[pbio-0020424-Turing1] Turing A (1936). On computable numbers, with an application to the Entscheidungsproblem. Proc Lond Math Soc.

[pbio-0020424-vonNeumann1] von Neumann J (1966). Theory of self-reproducing automata. Edited and completed by AW Burks.

[pbio-0020424-Wang1] Wang H (1961). Proving theorems by pattern recognition II. Bell System Tech J.

[pbio-0020424-Wang2] Wang H (1962). An unsolvable problem on dominoes. Technical report BL-30 (II-15).

[pbio-0020424-Wang3] Wang JC (1979). Helical repeat of DNA in solution. Proc Natl Acad Sci U S A.

[pbio-0020424-Whitesides1] Whitesides GM, Mathias JP, Seto CT (1991). Molecular self-assembly and nanochemistry: A chemical strategy for the synthesis of nanostructures. Science.

[pbio-0020424-Winfree1] Winfree E, Lipton RJ, Baum EB (1996). On the computational power of DNA annealing and ligation. DNA-based computers.

[pbio-0020424-Winfree2] Winfree E (1998a). Simulations of computing by self-assembly. Technical report CS-TR:1998.22.

[pbio-0020424-Winfree3] Winfree E (1998b). Whiplash PCR for *O*(1) computing. Technical report CS-TR:1998.23.

[pbio-0020424-Winfree4] Winfree E, Bekbolatov R, Chen J, Reif J (2004). Proofreading tile sets: Error-correction for algorithmic self-assembly. DNA computing 9.

[pbio-0020424-Winfree5] Winfree E, Liu F, Wenzler LA, Seeman NC (1998a). Design and self-assembly of two-dimensional DNA crystals. Nature.

[pbio-0020424-Winfree6] Winfree E, Yang X, Seeman NC, Landweber LF, Baum EB (1998b). Universal computation via self-assembly of DNA: Some theory and experiments. DNA-based computers II.

[pbio-0020424-Wolfram1] Wolfram S (1986). Random sequence generation by cellular automata. Adv Appl Math.

[pbio-0020424-Yan1] Yan H, LaBean TH, Feng L, Reif JH (2003a). Directed nucleation assembly of DNA tile complexes for barcode-patterned lattices. Proc Natl Acad Sci U S A.

[pbio-0020424-Yan2] Yan H, Park SH, Finkelstein G, Reif JH, LaBean TH (2003b). DNA-templated self-assembly of protein arrays and highly conductive nanowires. Science.

