# Supplementary material for: Algorithmic Self-Assembly of DNA Sierpinski Triangles
Source: PLoS Biol. 2004 Dec 7;2(12):e424. doi: 10.1371/journal.pbio.0020424 (PMC534809; doi:10.1371/journal.pbio.0020424)
Supplement: Figure S1 — (57 KB PDF). [file pbio.0020424.sg001.pdf]

Algorithmic Self-Assembly of DNA Sierpinski Triangles

Supporting Figures

Paul W. K. Rothemund[1], **Nick Papadakis**[2], **Erik Winfree**[1,2*]

1 Computation and Neural Systems, and **2** Computer Science, California Institute of Technology, Pasadena, California, United States of America

Citation: Rothemund PWK, Papadakis N, Winfree E (2004) Algorithmic Self-Assembly of DNA Sierpinski Triangles. PLoS Biol 2(?):e???.

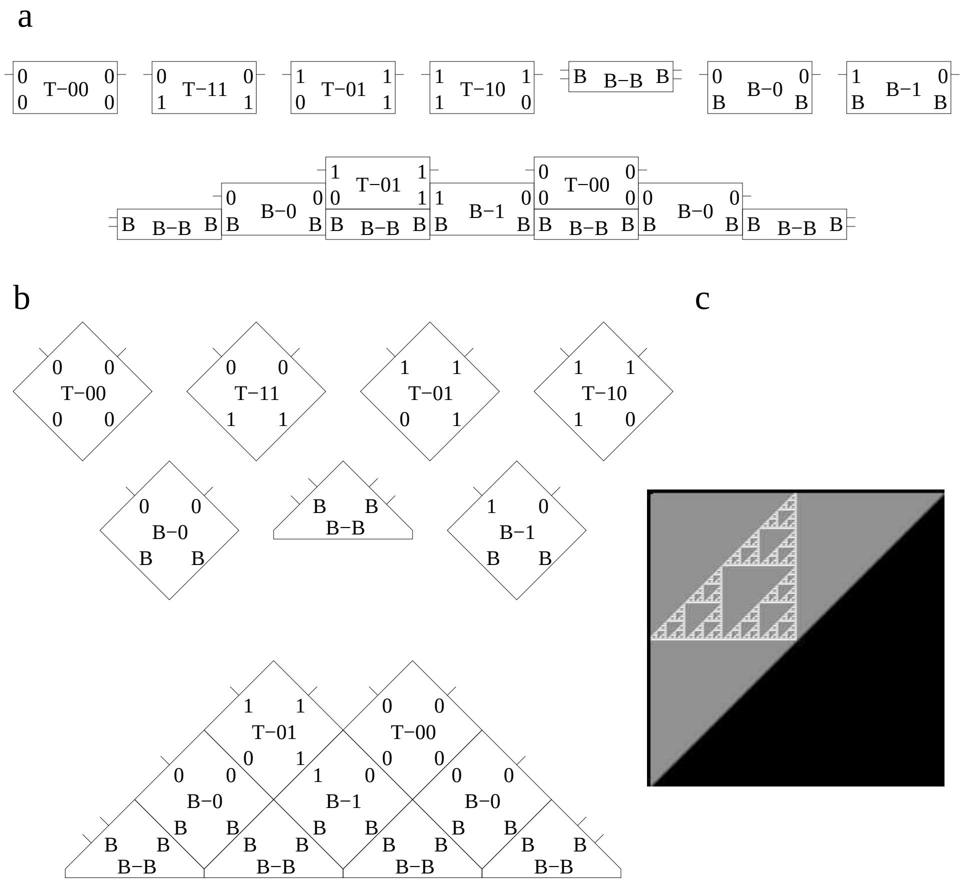

Figure S1: Representations and tile sets used in simulations. **(a)** Rectangular rendition of the tiles used in the kTAM simulations. Bond strengths (either 1 or 2) are indicated on output binding domains by the number of pins. **(b)** Square rendition of the tiles used by the kTAM simulator, `xgrow`. **(c)** Error-free Sierpinski triangle growth from a border, shown in the orientation used by `xgrow`, i.e., rotated 45° counterclockwise from b.
