# Supplementary material for: Algorithmic Self-Assembly of DNA Sierpinski Triangles
Source: PLoS Biol. 2004 Dec 7;2(12):e424. doi: 10.1371/journal.pbio.0020424 (PMC534809; doi:10.1371/journal.pbio.0020424)
Supplement: Figure S2 — (160 KB PDF). [file pbio.0020424.sg002.pdf]

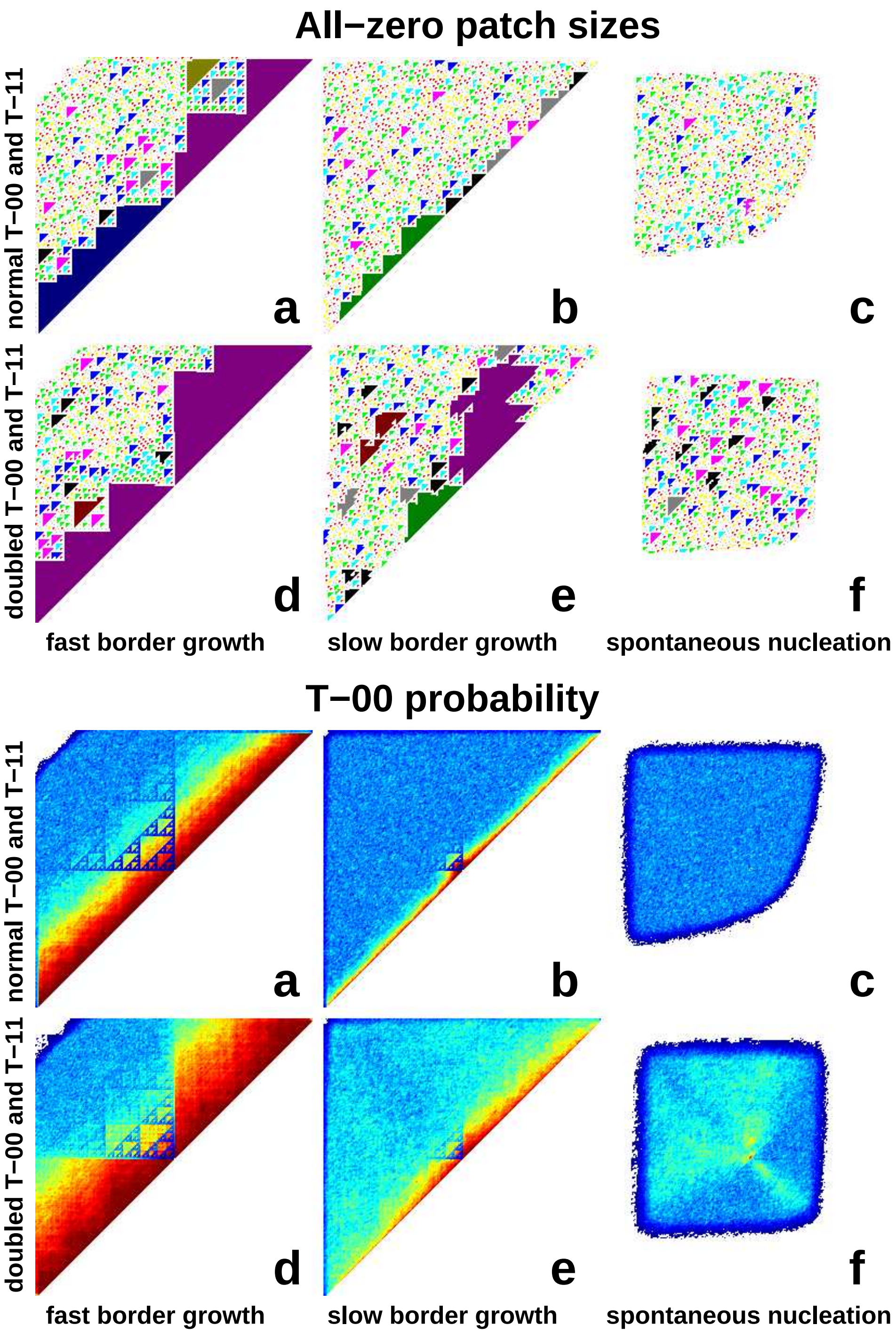

Figure S2: Behavior of simulated crystal growth. The top panel shows a sample run for each condition, with the all-'0' patches identified and colored according to their size. Orientation of the tiles is as in Figure S1c. The bottom panel shows the probability of observing a T-00 tile, estimated from 100 runs. Scale: 1.0 (red) to 0.0 (dark blue). The Sierpinski triangle appears as a pattern of decreased probability of observing a T-00 tile—under error-free growth, the probability would be zero. **(a)** Growth as in Figure 2b **(b)** Growth as in Figure 2b, but with slow border growth. **(c)** Growth as in Figure 2e. **(d)** Growth as in Figure 2c, but with fast border growth. **(e)** Growth as in Figure 2c. **(f)** Growth as in Figure 2f. Characteristic errors terminating Sierpinski triangles at corners are almost exclusively found under conditions e.
