# Supplementary material for: Algorithmic Self-Assembly of DNA Sierpinski Triangles
Source: PLoS Biol. 2004 Dec 7;2(12):e424. doi: 10.1371/journal.pbio.0020424 (PMC534809; doi:10.1371/journal.pbio.0020424)
Supplement: Figure S3 — (126 KB PDF). [file pbio.0020424.sg003.pdf]

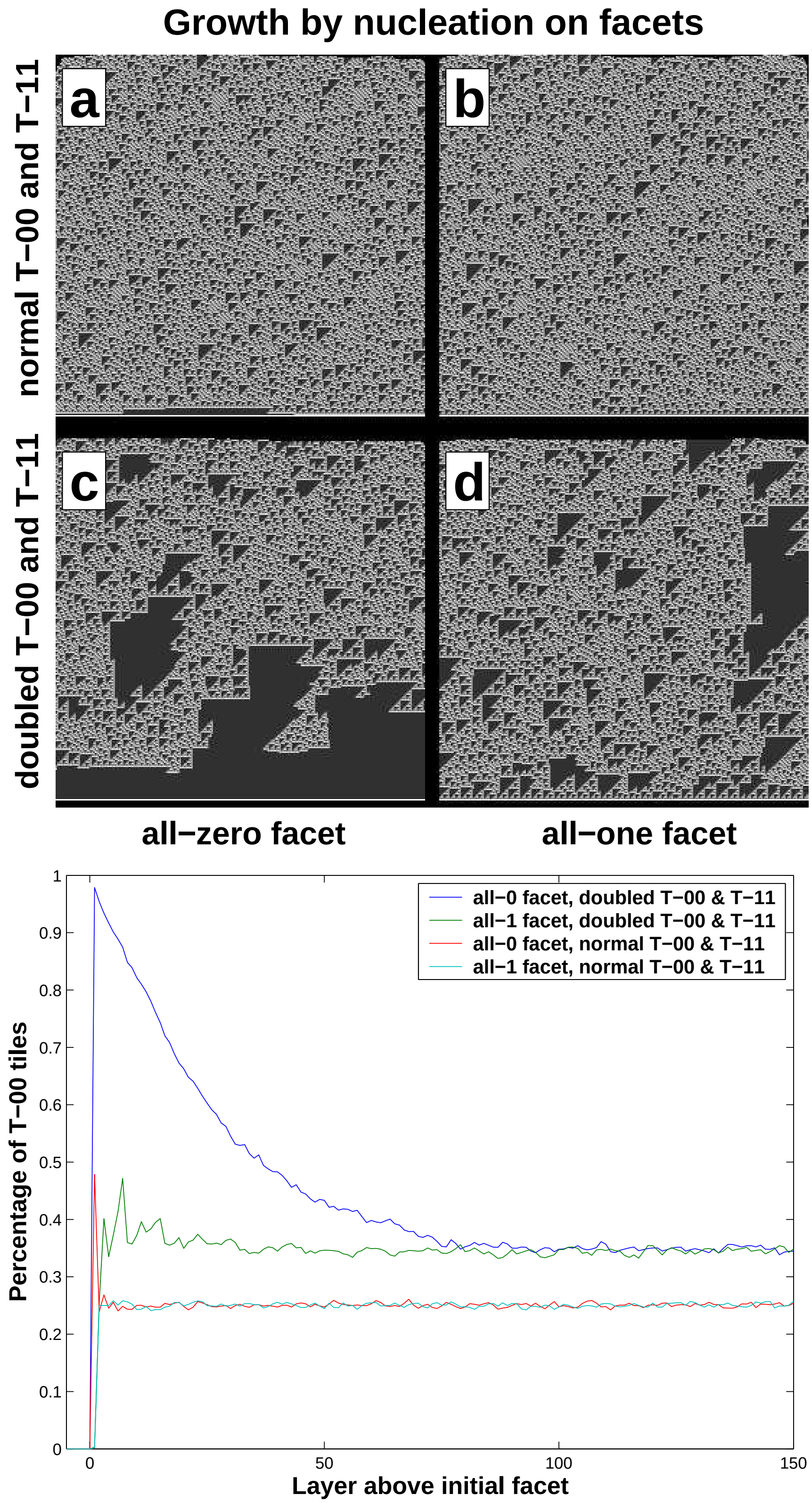


Figure S3: Simulations of growth on large facets. **(a-d)** Example runs. The bottom row is the pre-existing facet (256 tiles) presenting either all '0' bond types or all '1' bond types. The T-00 and T-11 tiles were either present at the normal concentration (as in Figure 2be) or at double the normal concentration (as in Figure 2cf). Simulations were performed at $G_{mc} = 17.0$ and $G_{se} = 8.6$, as in Figure 2c. Orientation of the tiles is as in Figure S1c. **(bottom)** Probability of observing a T-00 tile $L$ layers above the facet, for each of the four cases, estimated from 100 runs.
