# Supplementary material for: Algorithmic Self-Assembly of DNA Sierpinski Triangles
Source: PLoS Biol. 2004 Dec 7;2(12):e424. doi: 10.1371/journal.pbio.0020424 (PMC534809; doi:10.1371/journal.pbio.0020424)
Supplement: Figure S4 — (16 KB PDF). [file pbio.0020424.sg004.pdf]

```
DAE-E system strands:

Rule tile strands.

 VE1          (37-mer,  377840 /M/cm @ 260nm) : CCATTCGGACGTTTGCGGTAAAGATTAGGACATTGAA
 VE2_EE00     (26-mer,  260540 /M/cm @ 260nm) : CTGGTTCCGAGCACCGAATGGAGGTA
 VE3          (42-mer,  412740 /M/cm @ 260nm) : TTACCGCAAACGTGGCGAGTGTGATACGACTACACCTAATCT
 VE4_EE00     (26-mer,  249800 /M/cm @ 260nm) : ACCAGTTCAATGTGGCGTTCATACCT
 VE5          (37-mer,  348140 /M/cm @ 260nm) : TGAACGCCTGTAGTCGTATCACACTCGCCTGCTCGGA

 UE1          (37-mer,  374540 /M/cm @ 260nm) : CGTTAAGGACGACGCAATTCTCACATCGGACGAGTAG
 UE2_EE11     (26-mer,  254240 /M/cm @ 260nm) : GTCTGTGGTTTCACCTTAACGAGGTA
 UE3          (42-mer,  404820 /M/cm @ 260nm) : AGAATTGCGTCGTGGTTGCTAGGTCTCGCTATCACCGATGTG
 UE4_EE11     (26-mer,  253840 /M/cm @ 260nm) : ACCAGCTACTCGTGGATCTATAATGC
 UE5          (37-mer,  378680 /M/cm @ 260nm) : ATAGATCCTGATAGCGAGACCTAGCAACCTGAAACCA

 RE1J         (59-mer,  553620 /M/cm @ 260nm) : CGTATTGGACATTTGCTCAGCGTTTTCGCTGAGCTTCCGTAGACCGACTGGACATCTTC
 RE1          (37-mer,  356360 /M/cm @ 260nm) : CGTATTGGACATTTCCGTAGACCGACTGGACATCTTC
 RE2_EE01     (26-mer,  242720 /M/cm @ 260nm) : CTGGTCCTTCACACCAATACGGCATT
 RE3          (42-mer,  430880 /M/cm @ 260nm) : TCTACGGAAATGTGGCAGAATCAATCATAAGACACCAGTCGG
 RE4          (26-mer,  273000 /M/cm @ 260nm) : CAGACGAAGATGTGGTAGTGGAATGC
 RE5          (37-mer,  348160 /M/cm @ 260nm) : CCACTACCTGTCTTATGATTGATTCTGCCTGTGAAGG
 RE5J         (59-mer,  549780 /M/cm @ 260nm) : CCACTACCTGTCTTCTTGCGACTTTTGTCGCAAGTTATGATTGATTCTGCCTGTGAAGG

 SE1J         (59-mer,  572120 /M/cm @ 260nm) : CTCAGTGGACAGCCTACTTACCTTTTGGTAAGTATTGTTCTGGAGCGTTGGACGAAACT
 SE1          (37-mer,  360300 /M/cm @ 260nm) : CTCAGTGGACAGCCGTTCTGGAGCGTTGGACGAAACT
 SE2          (26-mer,  256620 /M/cm @ 260nm) : GTCTGGTAGAGCACCACTGAGGCATT
 SE3          (42-mer,  415380 /M/cm @ 260nm) : CCAGAACGGCTGTGGCTAAACAGTAACCGAAGCACCAACGCT
 SE4_EE10     (26-mer,  249220 /M/cm @ 260nm) : CAGACAGTTTCGTGGTCATCGTACCT
 SE5          (37-mer,  336840 /M/cm @ 260nm) : CGATGACCTGCTTCGGTTACTGTTTAGCCTGCTCTAC
 SE5J         (59-mer,  539060 /M/cm @ 260nm) : CGATGACCTGCTTCATGTCGGCTTTTGCCGACATTTGGTTACTGTTTAGCCTGCTCTAC

Cap and input tile strands for use with R-type nucleating strands.

 CapNRERE     (37-mer,  398960 /M/cm @ 260nm) : GATAGATGAGAGATTGAGTATAGTGTTGTTTGATAAG
 CapNUERE     (37-mer,  400000 /M/cm @ 260nm) : AGTGAATAGAAATGAATTGTAAAGTTGTGAGGTGTTA

 NRE1         (37-mer,  376320 /M/cm @ 260nm) : ATGCCAGGACGTTCGCAGCAGTCAACAGGACGATCAA
 NRE2         (26-mer,  261360 /M/cm @ 260nm) : TGGTTAGTTTGGACCTGGCATAGGTA
 NRE3         (42-mer,  424300 /M/cm @ 260nm) : CTGCTGCGAACGTGGAAGTGATGTAAGATATGGACCTGTTGA
 NRE4         (26-mer,  266160 /M/cm @ 260nm) : CAGACTTGATCGTGGTAGGTGGATTA

 NUE1         (37-mer,  382040 /M/cm @ 260nm) : CGAACTGGACGAAGGCAAGCGTGACAAGGACCGTTAG
 NUE2         (26-mer,  268540 /M/cm @ 260nm) : TGGTTGATGGAGACCAGTTCGAGGTA
 NUE3         (42-mer,  404120 /M/cm @ 260nm) : CGCTTGCCTTCGTGGATTTGAATGGTAATGTAGACCTTGTCA
 NUE4         (26-mer,  272940 /M/cm @ 260nm) : ACCAGCTAACGGTGGTTAAGAGTAGG

Splint strands for making R-type nucleating strands with assembly PCR.

 SplintNREUE2 (40-mer,  414660 /M/cm @ 260nm) : GTGTTGTTTGATAAGTGGTTGATGGAGAGGATTTGAATGG
 SplintNUERE2 (40-mer,  419340 /M/cm @ 260nm) : AGTTGTGAGGTGTTATGGTTAGTTTGGAGGAAGTGATGTA

 SplintNUEUE2 (40-mer,  418300 /M/cm @ 260nm) : AGTTGTGAGGTGTTATGGTTGATGGAGAGGATTTGAATGG
 SplintNRERE1 (40-mer,  441320 /M/cm @ 260nm) : GTAAGATATGGAGGTAGGTGGATTAGATAGATGAGAGATT
 SplintNUERE1 (40-mer,  443880 /M/cm @ 260nm) : TGGTAATGTAGAGGTTAAGAGTAGGAGTGAATAGAAATGA
 BridgeNRERE  (47-mer,  455640 /M/cm @ 260nm) : AACCACTTATCAAACAACACTATACTCAATCTCTCATCTATCTAATC
 BridgeNUERE  (47-mer,  446840 /M/cm @ 260nm) : AACCATAACACCTCACAACTTTACAATTCATTTCTATTCACTCCTAC

 NRE5         (37-mer,  335860 /M/cm @ 260nm) : CACCTACCTCCATATCTTACATCACTTCCTCCAAACT
 NUE5         (37-mer,  339240 /M/cm @ 260nm) : TCTTAACCTCTACATTACCATTCAAATCCTCTCCATC
```

Figure S4: DAE-E sequences.
