# Supplementary material for: Algorithmic Self-Assembly of DNA Sierpinski Triangles
Source: PLoS Biol. 2004 Dec 7;2(12):e424. doi: 10.1371/journal.pbio.0020424 (PMC534809; doi:10.1371/journal.pbio.0020424)
Supplement: Figure S5 — (21 KB PDF). [file pbio.0020424.sg005.pdf]

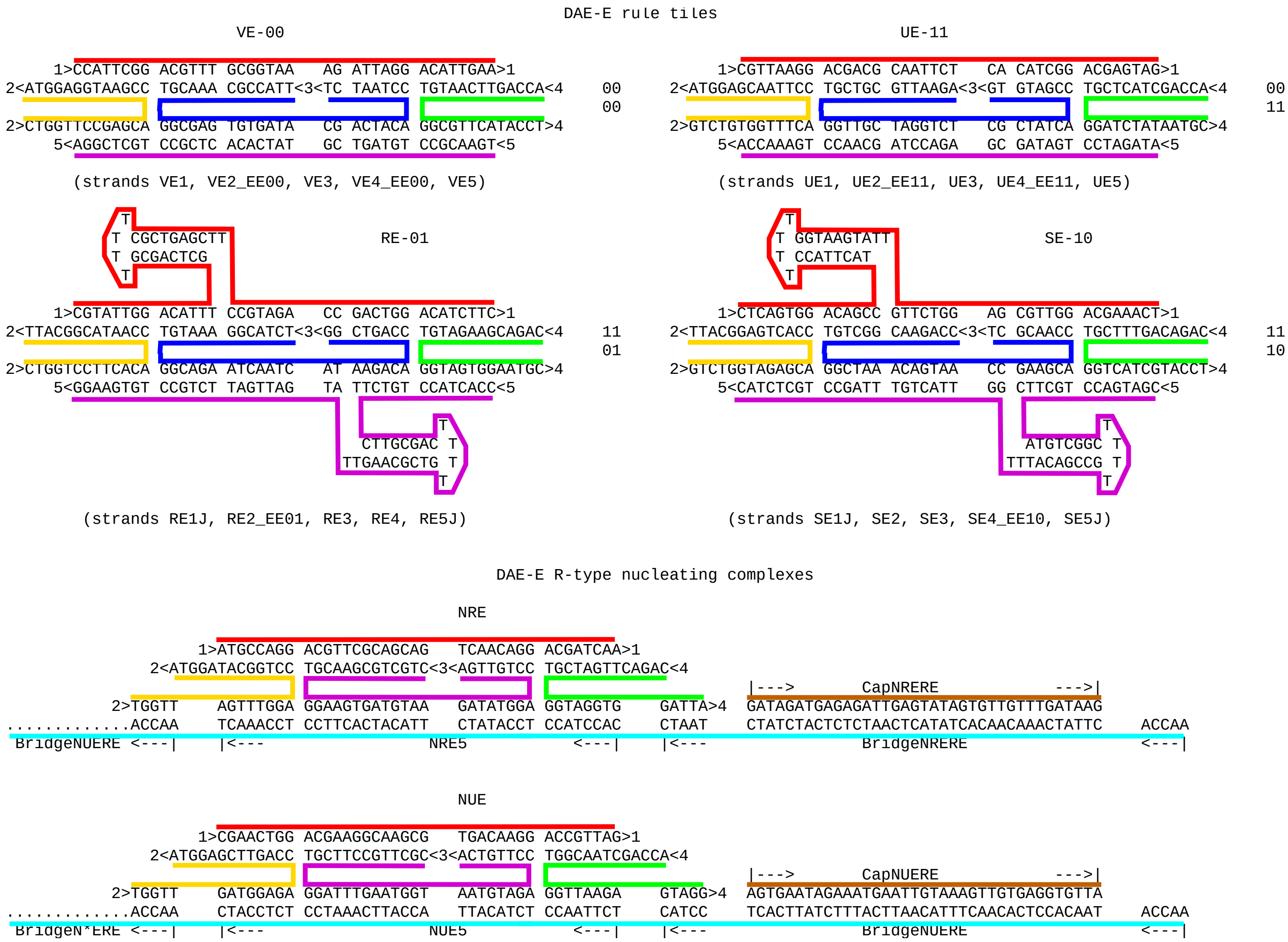

Figure S5: DAE-E diagrams. Arrows point 5′ to 3′. Component subsequences of the nucleating strand are indicated.
