# Supplementary material for: Algorithmic Self-Assembly of DNA Sierpinski Triangles
Source: PLoS Biol. 2004 Dec 7;2(12):e424. doi: 10.1371/journal.pbio.0020424 (PMC534809; doi:10.1371/journal.pbio.0020424)
Supplement: Figure S6 — (16 KB PDF). [file pbio.0020424.sg006.pdf]

```
DAO-E system strands:

Rule tile strands.

 R00_1    (26-mer,  255580 /M/cm @ 260nm) : TCACTCTACCGCACCAGAATGGAGAT
 R00_2    (48-mer,  460620 /M/cm @ 260nm) : CATTCTGGACGCCATAAGATAGCACCTCGACTCATTTGCCTGCGGTAG
 R00_3    (48-mer,  477220 /M/cm @ 260nm) : CAGTAGCCTGCTATCTTATGGCGTGGCAAATGAGTCGAGGACGGATCG
 R00_4    (26-mer,  248640 /M/cm @ 260nm) : TCACTCGATCCGTGGCTACTGGAGAT

 S00_1    (26-mer,  254080 /M/cm @ 260nm) : AGTGAGGCAATCCACAACCGCATCTC
 S00_2    (48-mer,  465300 /M/cm @ 260nm) : GCGGTTGTCCAACTTACCAGATCCACAAGCCGACGTTACAGGATTGCC
 S00_3    (48-mer,  456880 /M/cm @ 260nm) : GCTCTACAGGATCTGGTAAGTTGGTGTAACGTCGGCTTGTCCGTTCGC
 S00_4    (26-mer,  266060 /M/cm @ 260nm) : AGTGAGCGAACGGTGTAGAGCATCTC

 R11_1    (26-mer,  235900 /M/cm @ 260nm) : TCACTCAAACGCACCACTCTGTCTTG
 R11_2    (48-mer,  472980 /M/cm @ 260nm) : CAGAGTGGACGAAAGCTCACGGCACCAAGTATCAGGTTCCTGCGTTTG
 R11_3    (48-mer,  458120 /M/cm @ 260nm) : CTGTAGCCTGCCGTGAGCTTTCGTGGAACCTGATACTTGGACGAGTTG
 R11_4    (26-mer,  240840 /M/cm @ 260nm) : TCACTCAACTCGTGGCTACAGTCTTG

 S11_1    (26-mer,  244160 /M/cm @ 260nm) : GTATGGCTCGGCACCTCAAACATCTC
 S11_2    (48-mer,  474920 /M/cm @ 260nm) : GTTTGAGGACGCTATGAACATCCACCTAAGCAGAGACACCTGCCGAGC
 S11_3    (48-mer,  465880 /M/cm @ 260nm) : CGAGTACCTGGATGTTCATAGCGTGGTGTCTCTGCTTAGGACGAATGC
 S11_4    (26-mer,  248380 /M/cm @ 260nm) : GTATGGCATTCGTGGTACTCGATCTC

 R01n_1   (26-mer,  261440 /M/cm @ 260nm) : CATACCGTTGGCACCGAAAGCGAGAT
 R01n_2   (48-mer,  442820 /M/cm @ 260nm) : GCTTTCGGACTCGATCTCCAGACACCTACTGCGGTTCACCTGCCAACG
 R01n_2JC (70-mer,  640400 /M/cm @ 260nm) : GCTTTCGGACTCGATCTCCGCTGCTTTTGCAGCGGATTTCCAGACACCTACTGCGGTTCACCTGCCAACG
 R01n_3JC (70-mer,  671480 /M/cm @ 260nm) : CGATGACCTGTCTGGAGCTACCGCTTTTGCGGTAGCTTGATCGAGTGGTGAACCGCAGTAGGACGCCTCG
 R01n_3   (48-mer,  473220 /M/cm @ 260nm) : CGATGACCTGTCTGGAGATCGAGTGGTGAACCGCAGTAGGACGCCTCG
 R01n_4   (26-mer,  248740 /M/cm @ 260nm) : CATACCGAGGCGTGGTCATCGTCTTG

 S01_1    (26-mer,  272900 /M/cm @ 260nm) : AGTGAGAACGACCACATCATCCAAGA
 S01_2    (48-mer,  456960 /M/cm @ 260nm) : GATGATGTCCTTGTAAACTTCGCCACTCTAATCGCAATCAGGTCGTTC
 S01_2JC  (70-mer,  655520 /M/cm @ 260nm) : GATGATGTCCTTGTAACGCTCTGCTTTTGCAGAGCGTTACTTCGCCACTCTAATCGCAATCAGGTCGTTC
 S01_3JC  (70-mer,  702640 /M/cm @ 260nm) : GAGCAACAGGCGAAGTCTCCATCGTTTTCGATGGAGTTTTACAAGGTGATTGCGATTAGAGTCCGTAAGC
 S01_3    (48-mer,  496340 /M/cm @ 260nm) : GAGCAACAGGCGAAGTTTACAAGGTGATTGCGATTAGAGTCCGTAAGC
 S01_4    (26-mer,  254480 /M/cm @ 260nm) : GTATGGCTTACGGTGTTGCTCCAAGA

Cap and input tile strands for use with R-type nucleating strands.

 cpBr1    (37-mer,  387260 /M/cm @ 260nm) : GTTGATGGAGTATAGTGTATTGGATGAAATGTTATGT
 A1S      (37-mer,  356120 /M/cm @ 260nm) : TCACTGCTGAAGGCAGAGGACTGTGCTGGACTTGGTC
 A2       (28-mer,  268000 /M/cm @ 260nm) : TGGTAATGTAAGGACCTCTGCCTTCAGC
 A4SV     (26-mer,  267800 /M/cm @ 260nm) : CATACGACCAAGTGGATTTGTAGGAT
 A4_S00   (26-mer,  261380 /M/cm @ 260nm) : TCACTGACCAAGTGGATTTGTAGGAT
 A3_nick  (20-mer,  203520 /M/cm @ 260nm) : GGTTGAATGACCAGCACAGT

Splint strands for making nucleating strands with assembly PCR.

 Sp1A     (40-mer,  422100 /M/cm @ 260nm) : TGAATGAGGATTTGTAGGATGTTGATGGAGTATAGTGTAT
 SpA1     (40-mer,  421860 /M/cm @ 260nm) : TATTGGATGAAATGTTATGTTGGTAATGTAAGGAGGTTGA
 Br1      (37-mer,  365600 /M/cm @ 260nm) : ACATAACATTTCATCCAATACACTATACTCCATCAAC
 A5       (37-mer,  350140 /M/cm @ 260nm) : ATCCTACAAATCCTCATTCAACCTCCTTACATTACCA
```

Figure S6: DAO-E sequences.
