# Supplementary material for: Algorithmic Self-Assembly of DNA Sierpinski Triangles
Source: PLoS Biol. 2004 Dec 7;2(12):e424. doi: 10.1371/journal.pbio.0020424 (PMC534809; doi:10.1371/journal.pbio.0020424)
Supplement: Figure S8 — (244 KB PDF). [file pbio.0020424.sg008.pdf]

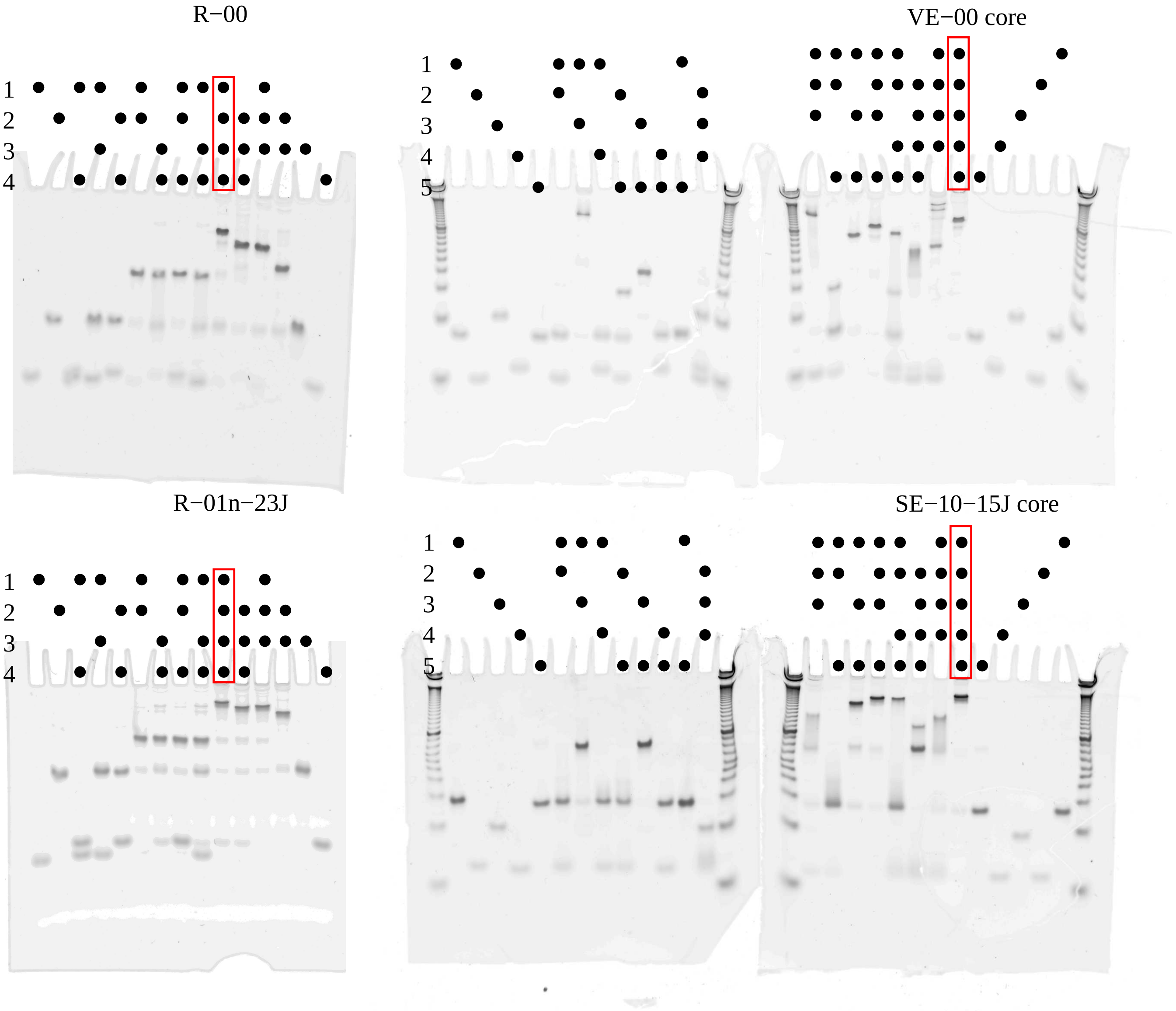


Figure S8: Formation gels for representative DAO-E and DAE-E tiles. Dots above each lane indicate which combination of strands was included in the annealing reaction. In most lanes, strands associate according to designed interactions only; e.g., in the DAO-E tiles, strands 2 and 4 run separately, while strands 1 and 2 run as a single heavy species. The red box indicates the lane containing all species, which should therefore form double-crossover molecules running as a single band. DAE-E formation gels are shown for tiles with different sticky ends but the same cores as VE-00 and SE-10-15J. Specifically, `VE2 (26-mer, 252260 /M/cm)` `CTGGTTCCGAGCACCGAATGGATACC`, `VE4 (26-mer, 251060 /M/cm)` `TGAGGTTCAATGTGGCGTTCATACCT`, and `SE4 (26-mer, 251920 /M/cm)` `TGAGGAGTTTCGTGGTCATCGTACCT` were used in place of the correspondingly-numbered strands.
