# Supplementary material for: Algorithmic Self-Assembly of DNA Sierpinski Triangles
Source: PLoS Biol. 2004 Dec 7;2(12):e424. doi: 10.1371/journal.pbio.0020424 (PMC534809; doi:10.1371/journal.pbio.0020424)
Supplement: Figure S9 — (22 KB PDF). [file pbio.0020424.sg009.pdf]

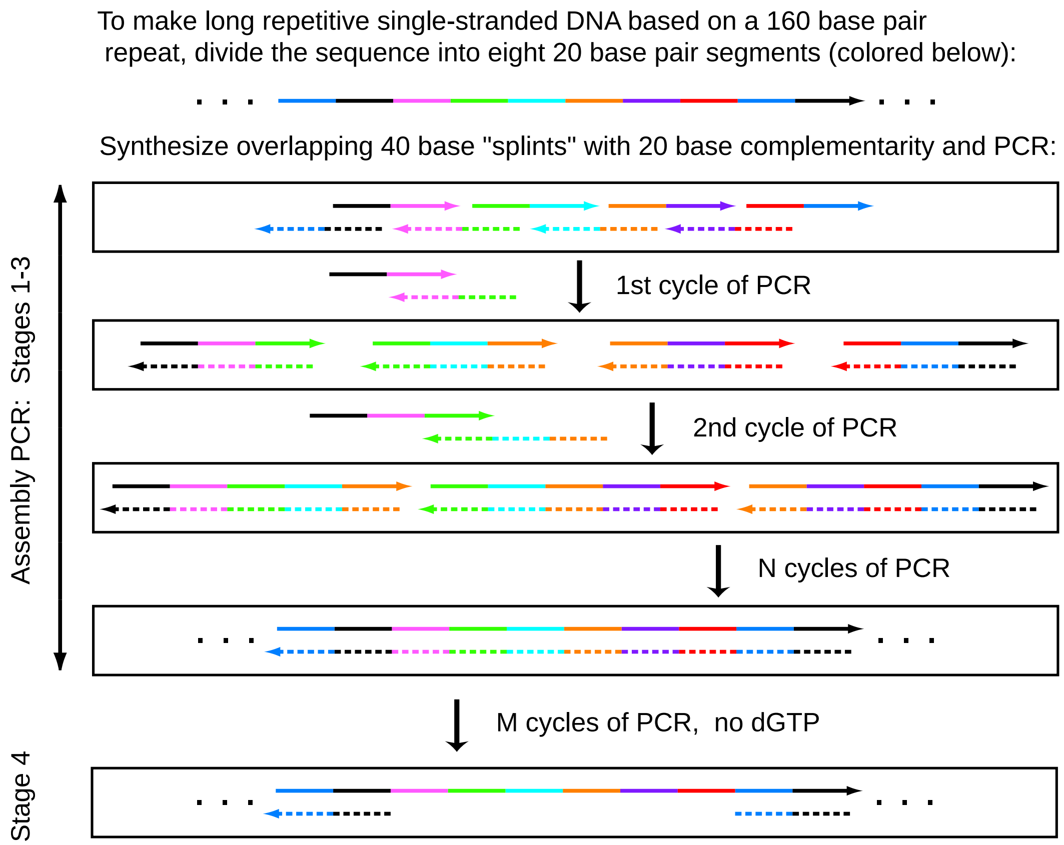

Figure S9: Using assembly PCR to generating long, repetitive, single-stranded DNA.
