# Supplementary material for: Algorithmic Self-Assembly of DNA Sierpinski Triangles
Source: PLoS Biol. 2004 Dec 7;2(12):e424. doi: 10.1371/journal.pbio.0020424 (PMC534809; doi:10.1371/journal.pbio.0020424)
Supplement: Figure S10 — (25 KB PDF). [file pbio.0020424.sg010.pdf]

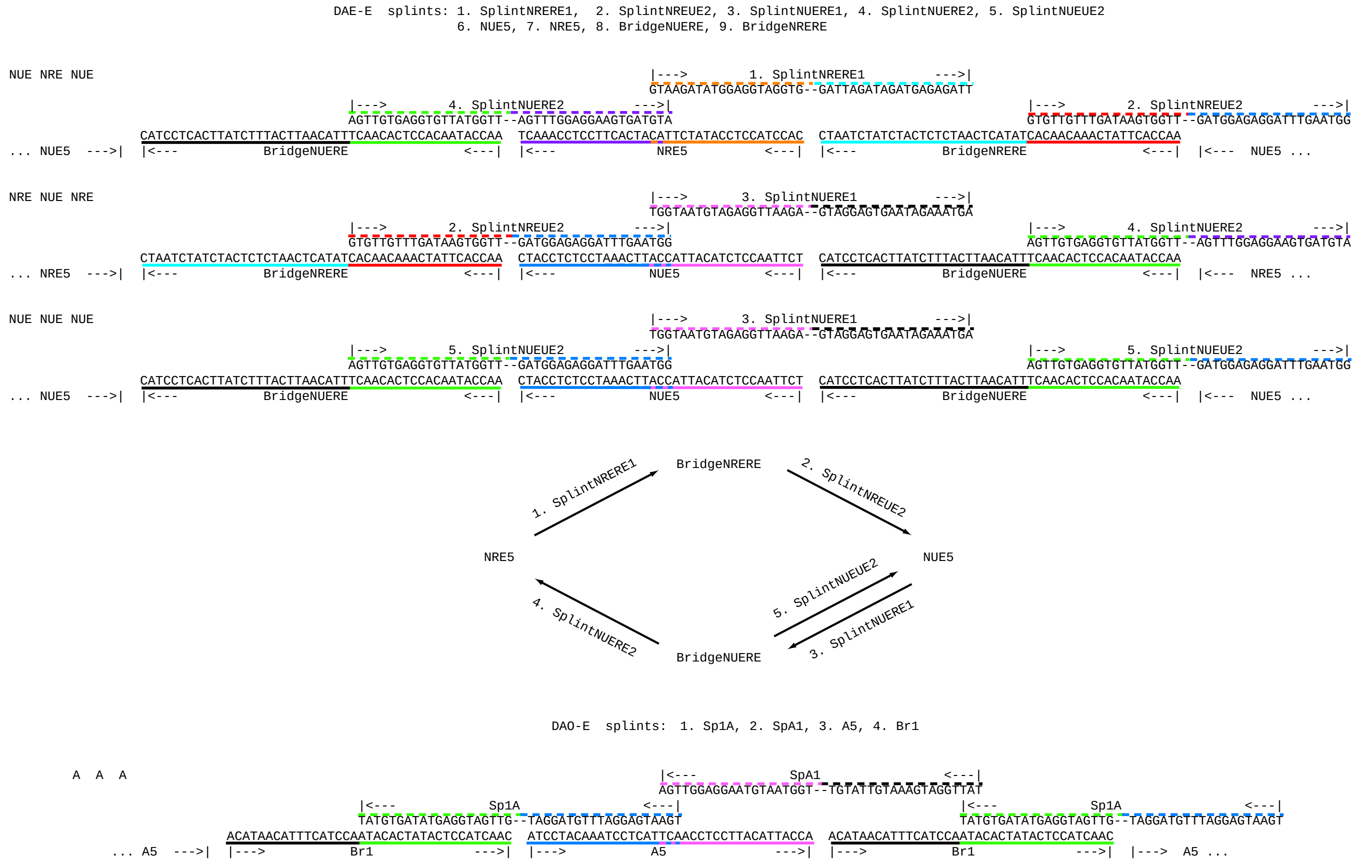

Figure S10: Assembly PCR scheme for DAE-E and DAO-E nucleating strands. In each case the bottom strand (solid) is the all-ACT nucleating strand drawn in an orientation to match the previous schema. In the DAE-E nucleating strand construction, due to the splint strands used in assembly PCR, BridgeNUERE always appears $3'$ of NRE5, while either BridgeNUERE or BridgeNRERE may appear $3'$ of NUE5. Thus, the sequence of input tiles determined by each nucleating strand is in the regular language (NRE NUE$^+$)*, as illustrated by the state transition diagram. The density of NRE5 subsequences (which output a '1' to their right) is determined by the proportion of SplintNREUE2 and SplintNUERE2 relative to other strands in the assembly PCR reaction. In the DAO-E nucleating strand construction, there is a single repeating sequence. The density of input tiles outputting a '1' is determined by the proportion of A4SV relative to A4-S00 in the input strand mix used during annealing.
