# Supplementary material for: Algorithmic Self-Assembly of DNA Sierpinski Triangles
Source: PLoS Biol. 2004 Dec 7;2(12):e424. doi: 10.1371/journal.pbio.0020424 (PMC534809; doi:10.1371/journal.pbio.0020424)
Supplement: Figure S11 — (53 KB PDF). [file pbio.0020424.sg011.pdf]

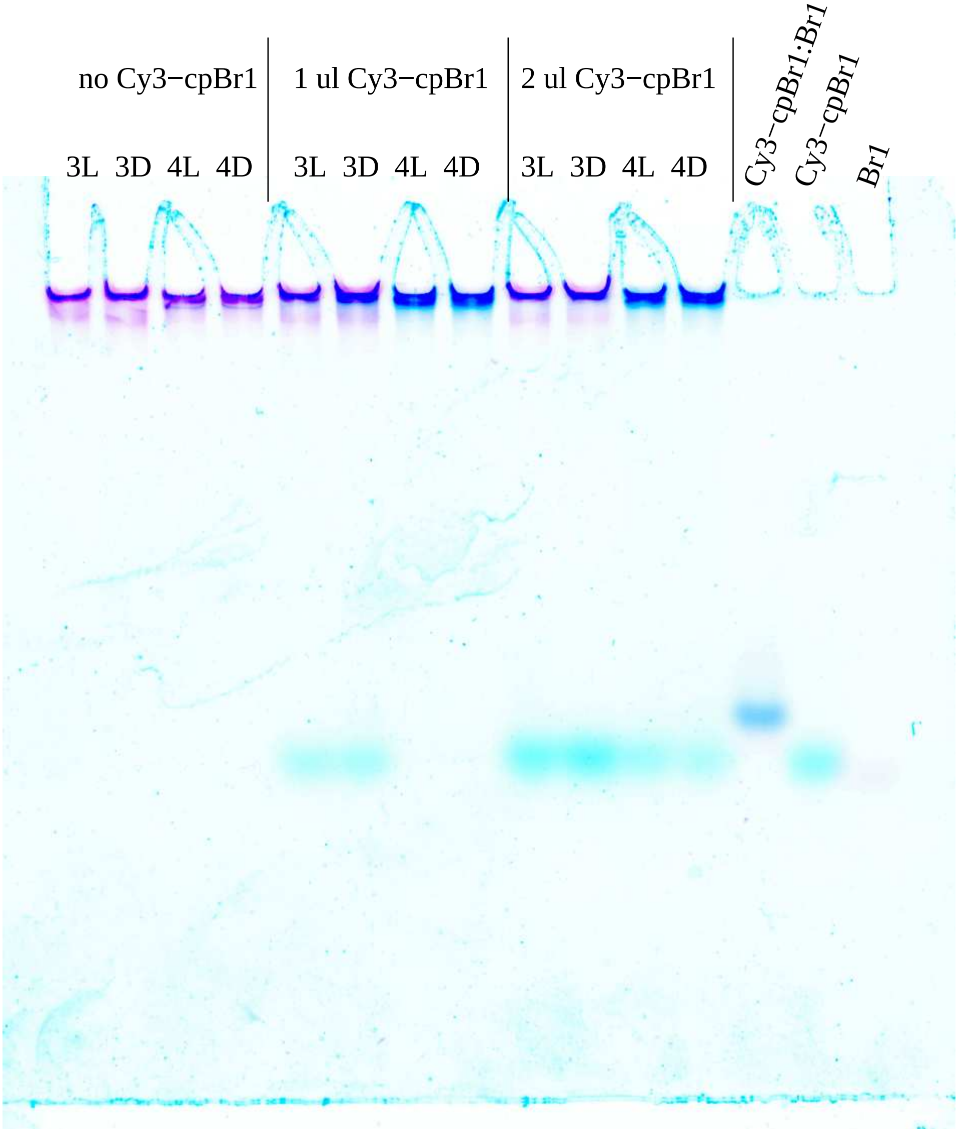

Figure S 11: Binding capacity gel for determining DAO-E nucleating strand stoichiometry. Lanes designated '3' contain double-stranded material purified after stage 3, lanes designated '4' contain material purified after stage 4. Lanes designated 'L' had Sybr green I added to the reaction mixture prior to PCR, and lanes designated 'D' had no Sybr Green I at this stage. The first set of four lanes acts as controls, demonstrating how the products of both stage 3 and stage 4 remain stuck in the wells. The second set of four lanes had 1 μL of Cy3-labelled cpBr1 added. The third set of four lanes had 2 μL of Cy3-labelled cpBr1 added. The final three lanes are controls: Cy3-cpBr1 complexed with its complement Br1, Cy3-cpBr1, and Br1 alone. The gel was post-stained with Sybr Green I and imaged under two conditions: (1) excitation with a 488 nm laser with emission recorded by a 530 nm bandpass filter resulting in the purple lanes—this captures the Sybr Green I emission and (2) excitation with a 532 nm laser with emission recorded by a 555 nm longpass filter resulting in the blue bands—this captures the Cy3 emission. Cyan false-color indicates fluorescence of Cy3-cpBr1. Purple false-color indicates fluorescence of Sybr Green I stain, which preferentially stains double-stranded material. For example, Br1 has the same mobility as Cy3-cpBr1, but stains only faintly.
