# Supplementary material for: Algorithmic Self-Assembly of DNA Sierpinski Triangles
Source: PLoS Biol. 2004 Dec 7;2(12):e424. doi: 10.1371/journal.pbio.0020424 (PMC534809; doi:10.1371/journal.pbio.0020424)
Supplement: Figure S12 — (33 KB PDF). [file pbio.0020424.sg012.pdf]

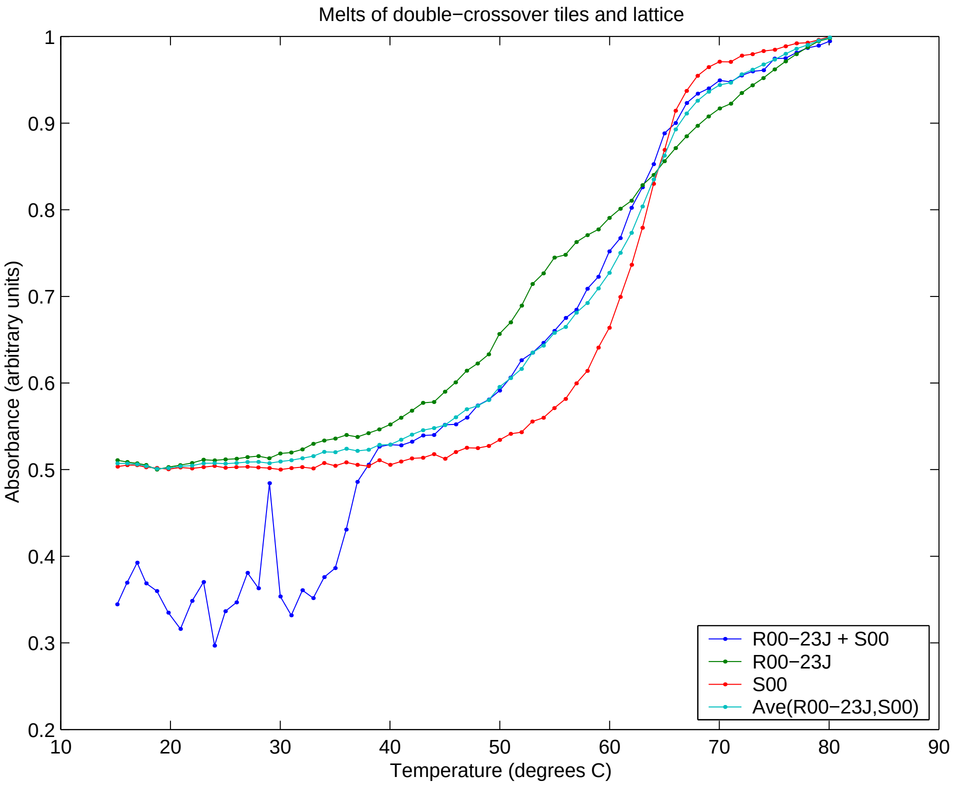

Figure S 12: Melts of R-00-23J and S-00 and their mixture. Tile R-00-23J has the same core as R-00, but replaces the correspondingly-numbered strands by `R00-2J (70-mer, 664820 /M/cm) CATTCTGGACGCCACGGTCAAGTTTTCTTGACCGTTTAAGATAGCACCTCGACTCATTTGCCTGCGGTAG`, and `R00-3J (70-mer, 681480 /M/cm) CAGTAGCCTGCTATCGGTTGTGTTTTCACAACCGTTCTTATGGCGTGGCAAATGAGTCGAGGACGGATCG`. Absorbance values were normalized to the maximum and minimum of the single-tile curves. The average of the R-00-23J curve and the S-00 curve is drawn in cyan; above 40°C it agrees with the melting curve of the R-00-23J + S-00 mixture, indicating that the melting temperature of this crystal is below 40°C at 0.2 μM.
