# Supplementary material for: Algorithmic Self-Assembly of DNA Sierpinski Triangles
Source: PLoS Biol. 2004 Dec 7;2(12):e424. doi: 10.1371/journal.pbio.0020424 (PMC534809; doi:10.1371/journal.pbio.0020424)
Supplement: Figure S13 — (234 KB PDF). [file pbio.0020424.sg013.pdf]

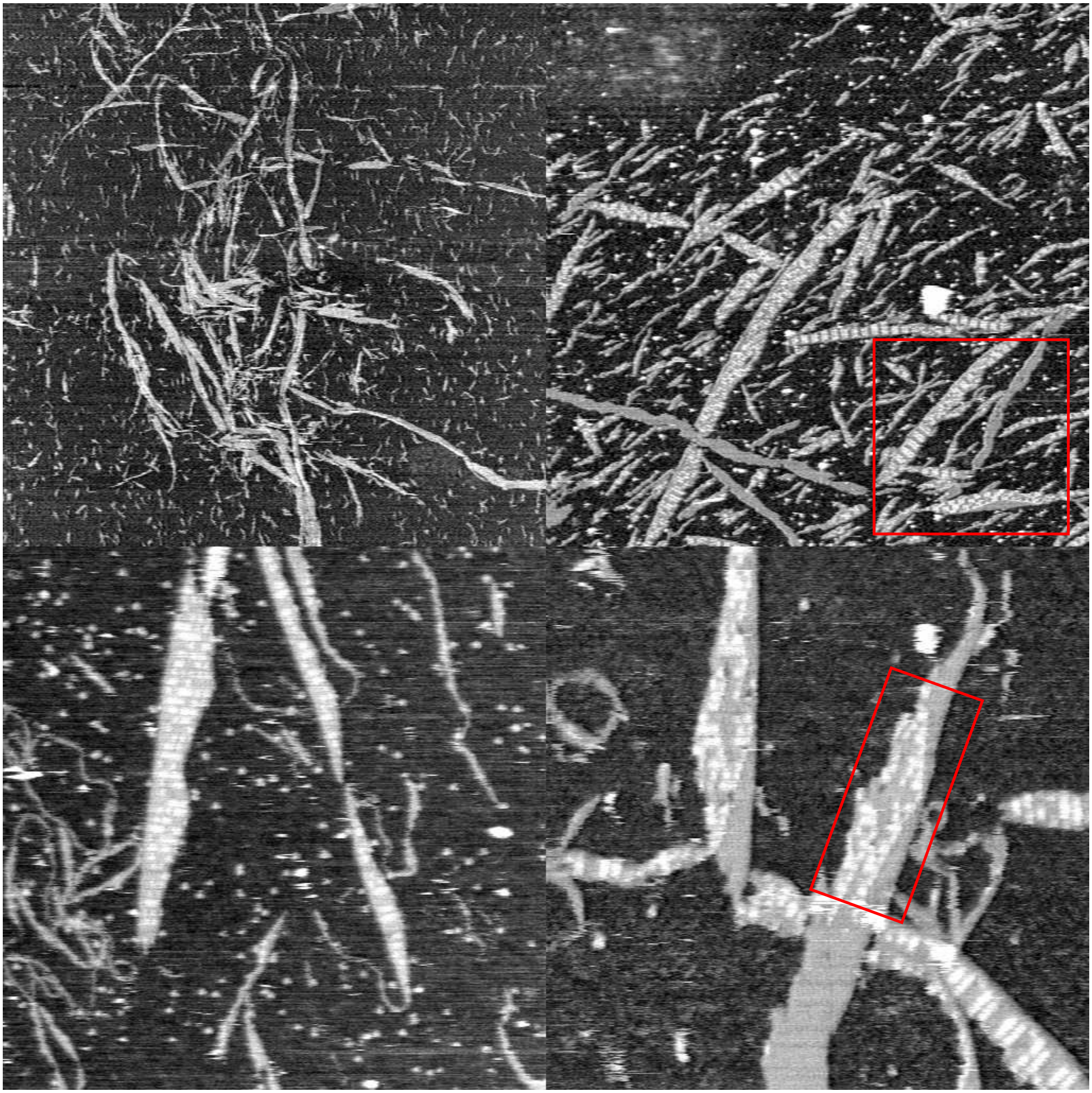

Figure S13: AFM images showing the context and distribution of DAE-E crystals. Upper left: 5.0 μm scan showing many long, thin crystals. Upper right: 2.3 μm scan showing the region surrounding Figure 5a (red box). Lower left: 830 nm scan showing faceting of templated crystals. Note the thin tails extending from several of the crystals. These may be regions of the nucleating strand / input tile complexes that have not yet grown as part of the crystals, or they may be regions of the nucleating strand that remain double-stranded after the asymmetric PCR step of the assembly PCR protocol. Lower right: 650 nm scan showing the region surrounding Figure 5c (red box).
