# Supplementary material for: Algorithmic Self-Assembly of DNA Sierpinski Triangles
Source: PLoS Biol. 2004 Dec 7;2(12):e424. doi: 10.1371/journal.pbio.0020424 (PMC534809; doi:10.1371/journal.pbio.0020424)
Supplement: Figure S14 — (203 KB PDF). [file pbio.0020424.sg014.pdf]

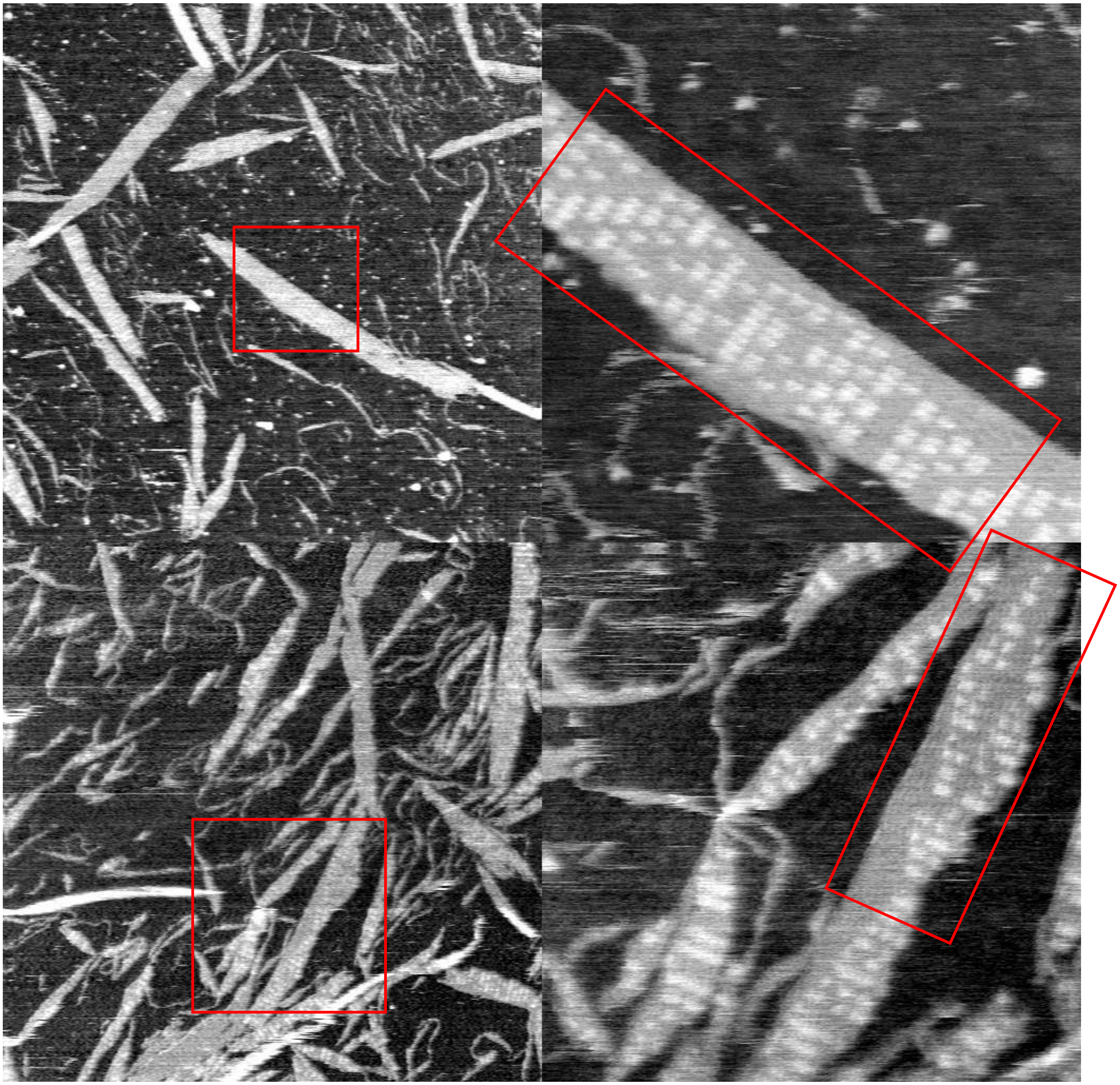

Figure S14: AFM images showing the context and distribution of DAE-E crystals. Upper left: 1.5 μm scan showing the region surrounding Figure 5b. (Red box shows area of upper right scan.) Upper right: 320 nm scan showing the region surrounding Figure 5b (red box). Lower left: 1.3 μm scan showing the region surrounding Figure 5d. (Red box shows area of lower right scan.) Lower right: 430 nm scan showing the region surrounding Figure 5d (red box).
