# Supplementary material for: Algorithmic Self-Assembly of DNA Sierpinski Triangles
Source: PLoS Biol. 2004 Dec 7;2(12):e424. doi: 10.1371/journal.pbio.0020424 (PMC534809; doi:10.1371/journal.pbio.0020424)
Supplement: Figure S15 — (226 KB PDF). [file pbio.0020424.sg015.pdf]

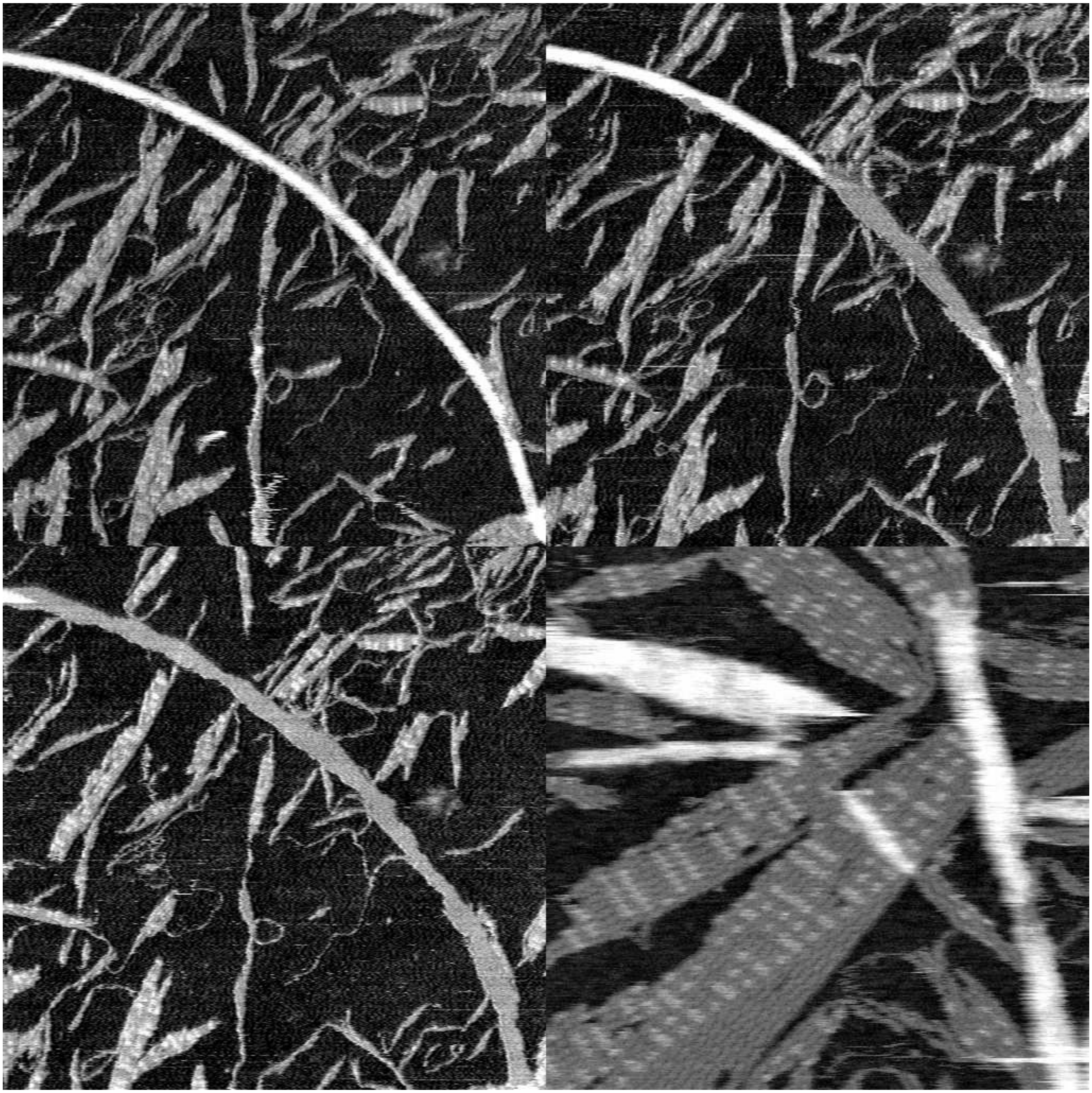

Figure S15: AFM images of DAE-E crystals and tubes. Upper left: 1.0 μm scan showing an unopened tube. The tube is roughly twice the height of other crystals. Upper right: Subsequent scan shows the tube partially opened. Opened domains are the same height as other crystals; closer examination reveals tiles whose long axis parallels the tube axis. Lower left: An even later scan of the same region reveals the tube completely opened. Lower right: 390 nm scan showing the region surrounding Figure 5e. Three unopened tubes (with circumferences of roughly 4, 8, and 17 tiles) can also be seen.
