# Supplementary material for: Algorithmic Self-Assembly of DNA Sierpinski Triangles
Source: PLoS Biol. 2004 Dec 7;2(12):e424. doi: 10.1371/journal.pbio.0020424 (PMC534809; doi:10.1371/journal.pbio.0020424)
Supplement: Figure S16 — (256 KB PDF). [file pbio.0020424.sg016.pdf]

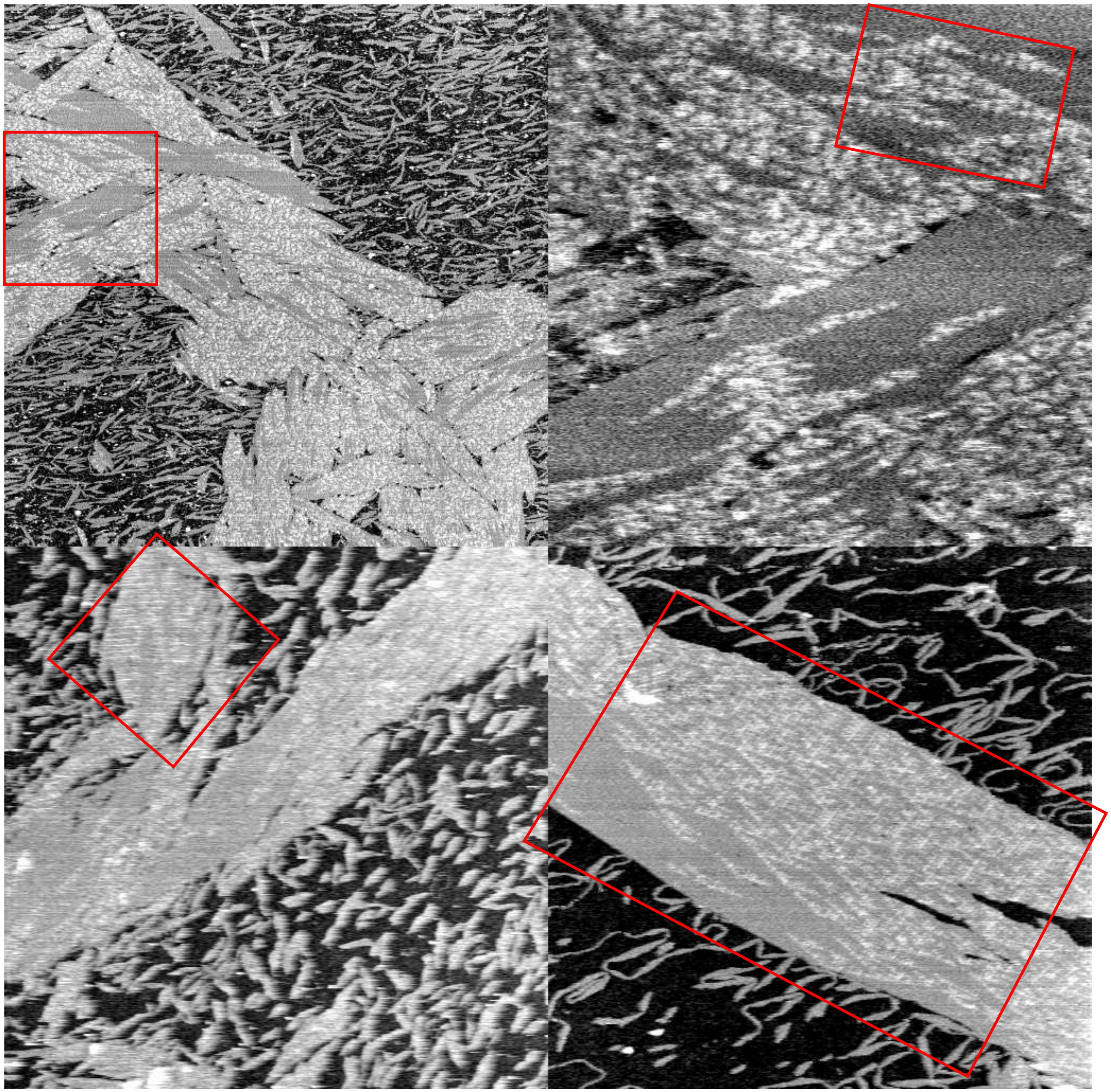

Figure S16: AFM images showing the context and distribution of DAO-E crystals. Upper left: 4.0 μm scan showing region surrounding Figure 6c. (Red box shows area of upper right scan.) Upper right: 500 nm scan showing region surrounding Figure 6d (red box). Lower left: 2.3 μm scan showing region surrounding Figure 6b (red box). Lower right: 1.8 μm scan showing the region surrounding Figure 6a (red box).
