# Supplementary material for: Algorithmic Self-Assembly of DNA Sierpinski Triangles
Source: PLoS Biol. 2004 Dec 7;2(12):e424. doi: 10.1371/journal.pbio.0020424 (PMC534809; doi:10.1371/journal.pbio.0020424)
Supplement: Figure S17 — (234 KB PDF). [file pbio.0020424.sg017.pdf]

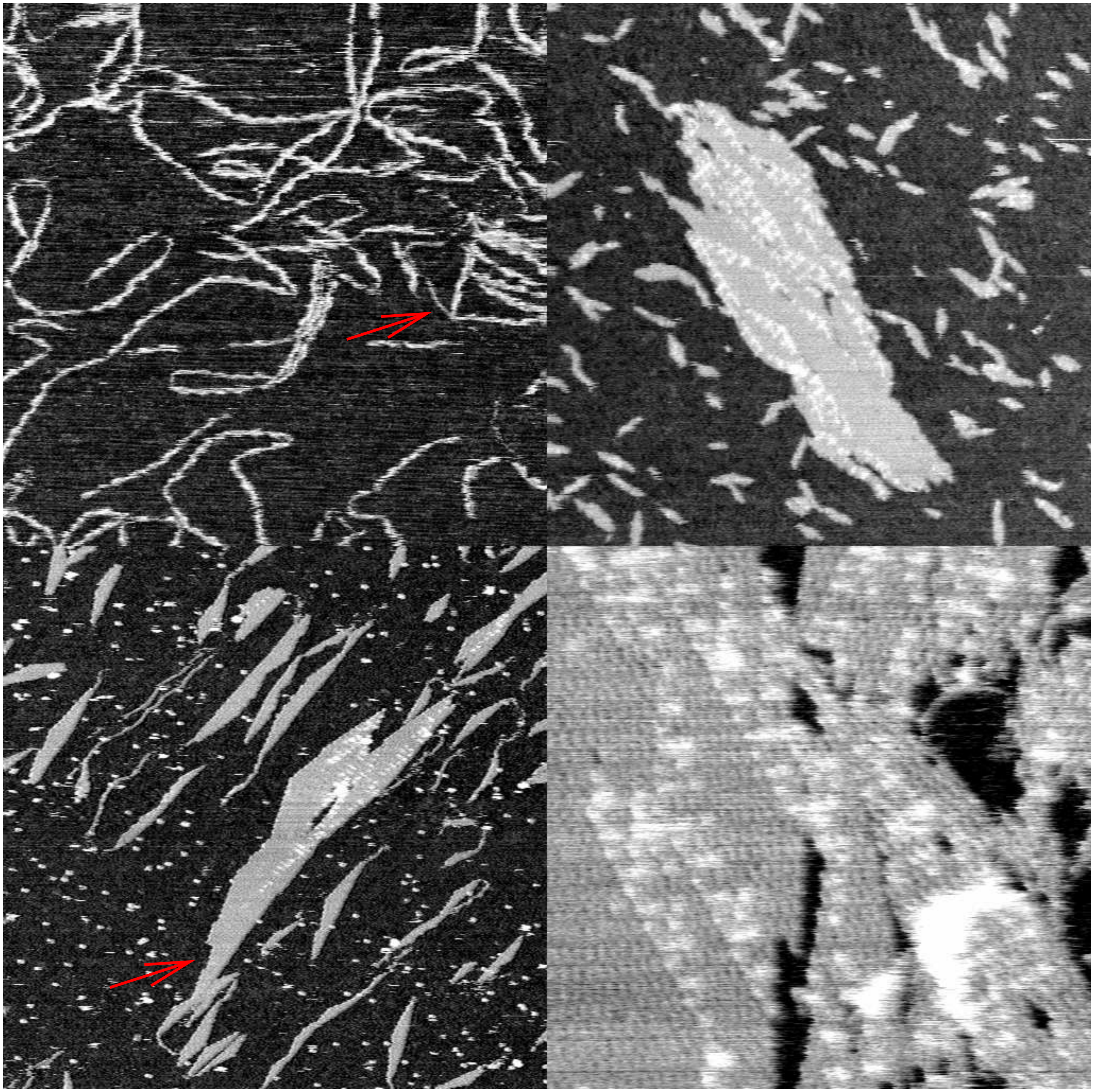

Figure S 17: AFM images of boundary assemblies and untemplated DAO-E crystals. Upper left: 750 nm scan showing nucleating strand + input tiles + S-00. Bumpy domains indicate the presence of input tiles and one layer of S-00. Thinner smooth domains (arrow) are assumed to be double-stranded, and hence without tiles. Upper right: 1.1 μm scan of a sample prepared with just 5 tiles (no S-11) and no nucleating structures. Therefore, this must be an untemplated crystal. (It could not be a ripped fragment of a templated crystal.) Lower left: 1.2 μm scan of a sample prepared with all 6 tiles. Nucleating structure tails can be seen. Crystals are particularly well faceted. Facet roughening can be observed (arrow). Lower right: 320 nm scan of a sample prepared with just 5 tiles (no S-11).
