# Supplementary material for: Algorithmic Self-Assembly of DNA Sierpinski Triangles
Source: PLoS Biol. 2004 Dec 7;2(12):e424. doi: 10.1371/journal.pbio.0020424 (PMC534809; doi:10.1371/journal.pbio.0020424)
Supplement: Figure S18 — (146 KB PDF). [file pbio.0020424.sg018.pdf]

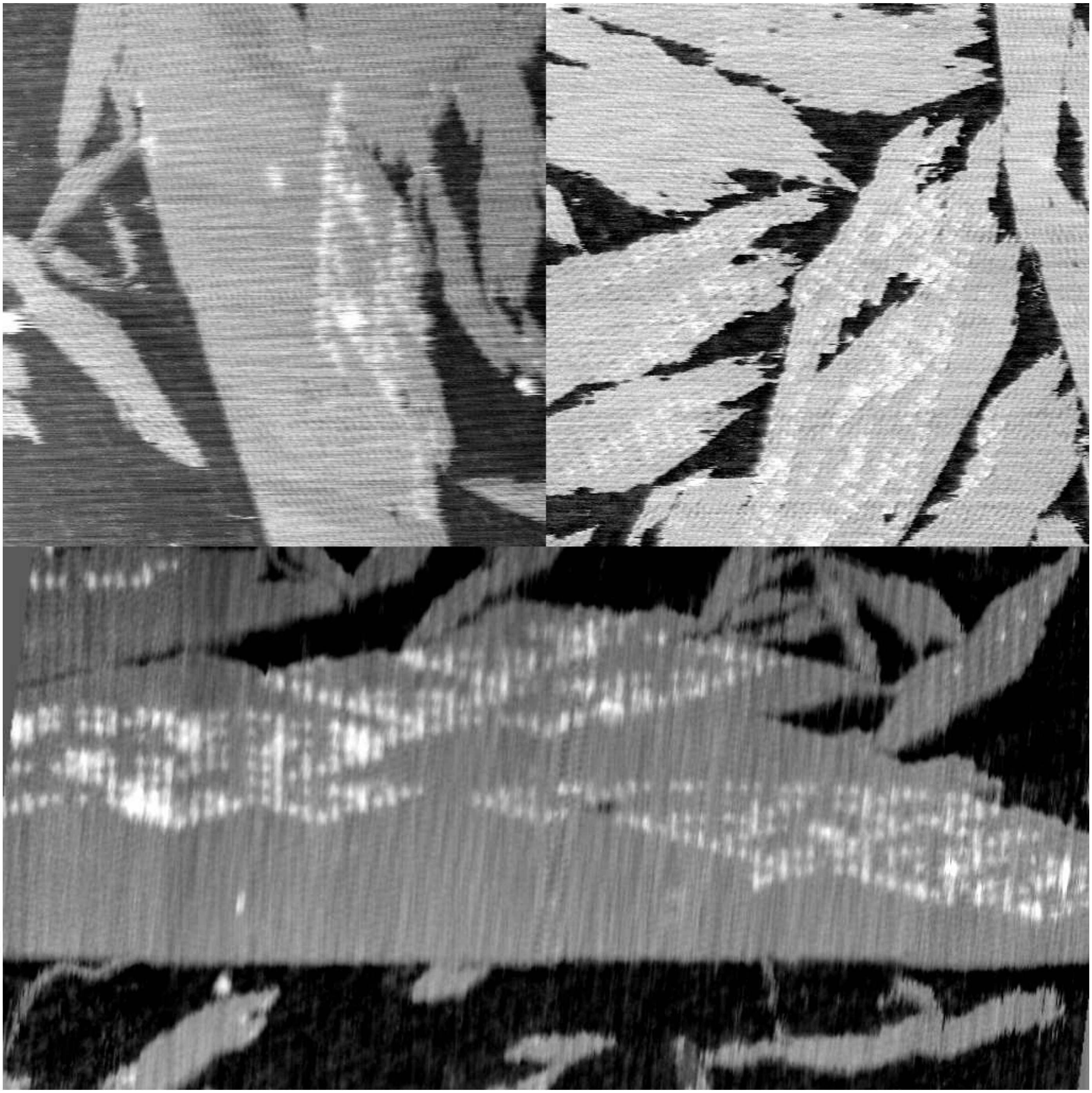

Figure S18: AFM images of DAO-E crystals grown under constant-temperature, near-constant concentration conditions. To construct thick rigid strips of '0' tiles as initial templates for growth, all-'0' nucleating structures were bulk annealed with R-00 and S-00 tiles. These strips had variable width and often were faceted. Once room temperature had been reached, at roughly hourly intervals a mix of 5 pre-formed rule tiles were added to boost tile concentrations by 4 to 10 nM. Presumably, during the interval between additions, tiles incorporate into crystals and therefore their concentrations decrease to the critical concentration, which we estimate to be between 4 to 10 nM. Despite our hopes, this procedure did not lead to measurably lower error rates, perhaps due to "sideways" growth on facets. Upper left: 510 nm scan. Upper right: 550 nm scan. Lower: 980 nm image composite from three scans. Experiments performed by Jason Rolfe.
