# Supplementary material for: Algorithmic Self-Assembly of DNA Sierpinski Triangles
Source: PLoS Biol. 2004 Dec 7;2(12):e424. doi: 10.1371/journal.pbio.0020424 (PMC534809; doi:10.1371/journal.pbio.0020424)
Supplement: Figure S19. Compiled Figures S1–S18 — (1.7 MB PDF). [file pbio.0020424.sg019.pdf]

Algorithmic Self-Assembly of DNA Sierpinski Triangles

Supporting Figures

Paul W. K. Rothemund[1,2], **Nick Papadakis**[2], **Erik Winfree**[1,2*]

1 Computation and Neural Systems, and **2** Computer Science, California Institute of Technology, Pasadena, California, United States of America

Citation: Rothemund PWK, Papadakis N, Winfree E (2004) Algorithmic Self-Assembly of DNA Sierpinski Triangles. PLoS Biol 2(12):e424.

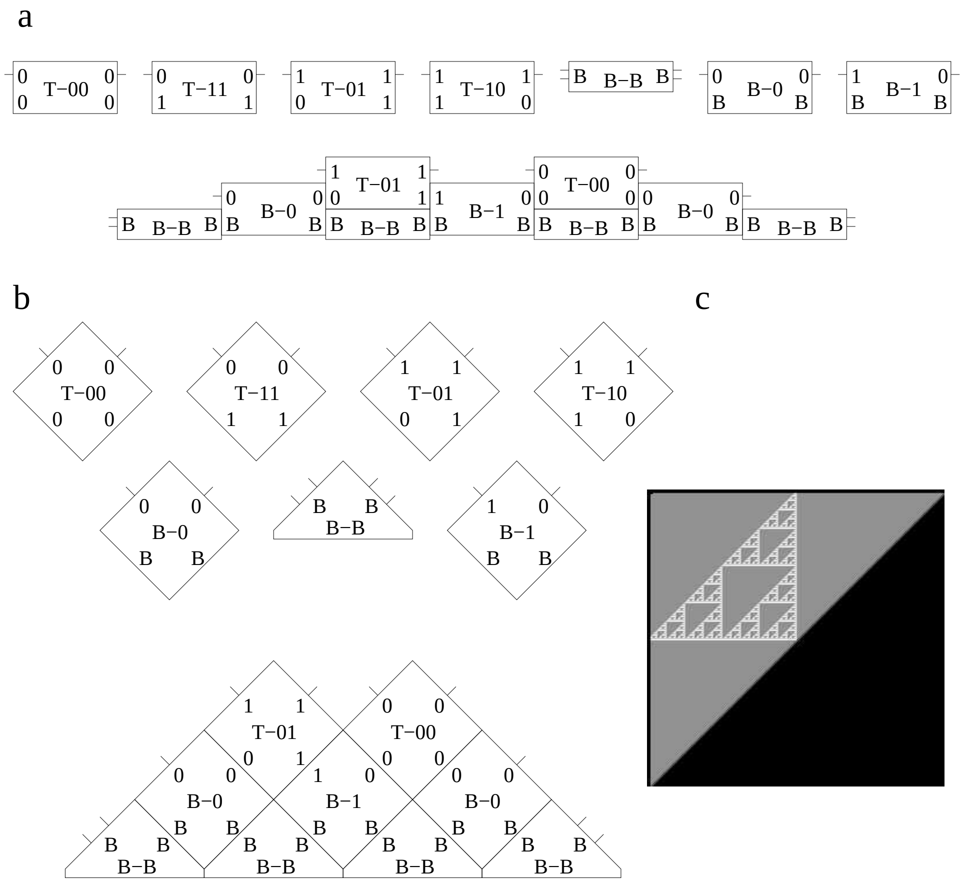

Figure S1: Representations and tile sets used in simulations. **(A)** Rectangular rendition of the tiles used in the kTAM simulations. Bond strengths (either 1 or 2) are indicated on output binding domains by the number of pins. **(B)** Square rendition of the tiles used by the kTAM simulator, `xgrow`. **(C)** Error-free Sierpinski triangle growth from a border, shown in the orientation used by `xgrow`, i.e., rotated 45° counterclockwise from B.

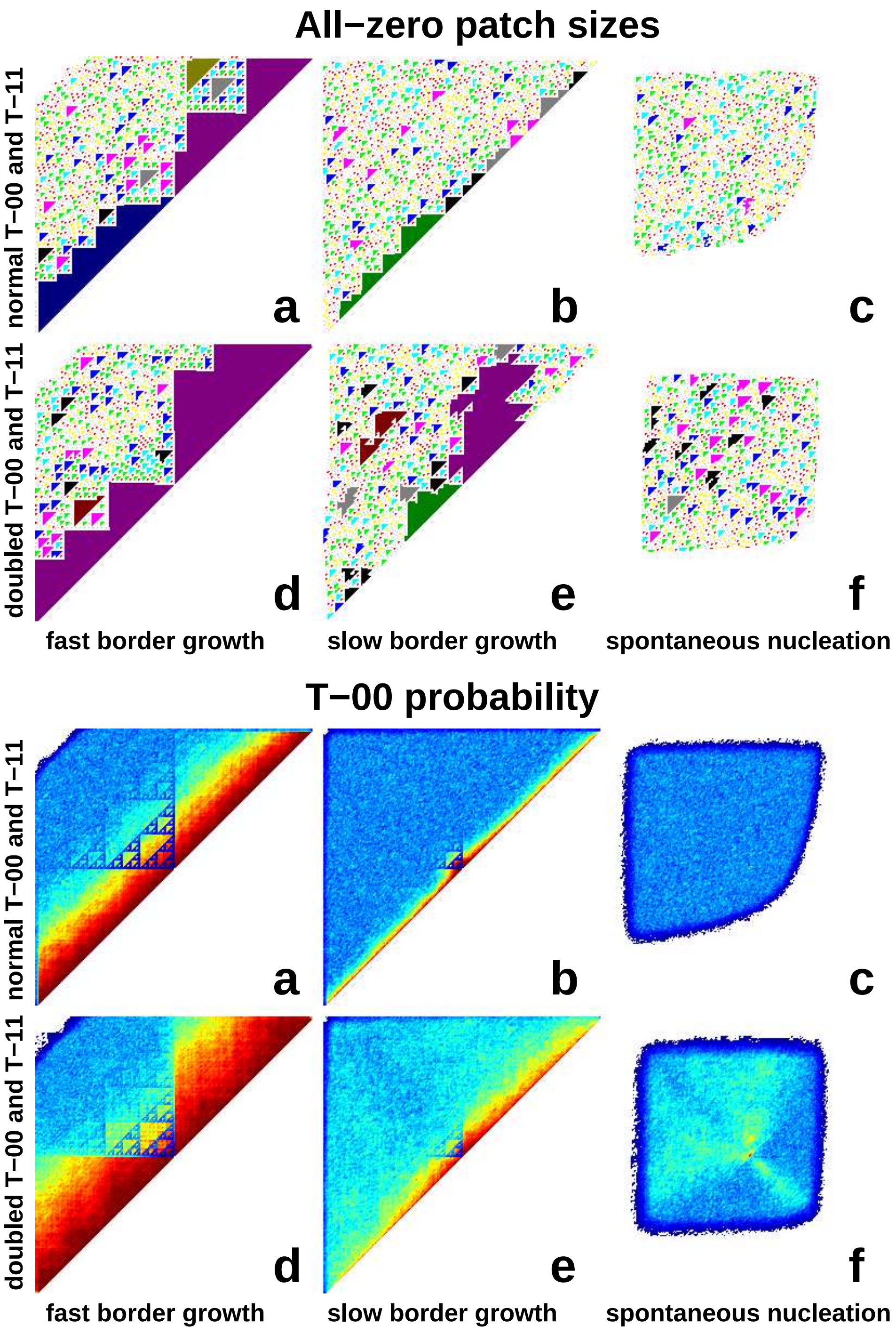

Figure S2: Behavior of simulated crystal growth. The top panel shows a sample run for each condition, with the all-'0' patches identified and colored according to their size. Orientation of the tiles is as in Figure S1C. The bottom panel shows the probability of observing a T-00 tile, estimated from 100 runs. Scale: 1.0 (red) to 0.0 (dark blue). The Sierpinski triangle appears as a pattern of decreased probability of observing a T-00 tile—under error-free growth, the probability would be zero. **(A)** Growth as in Figure 2B **(B)** Growth as in Figure 2B, but with slow border growth. **(C)** Growth as in Figure 2E. **(D)** Growth as in Figure 2C, but with fast border growth. **(E)** Growth as in Figure 2C. **(F)** Growth as in Figure 2F. Characteristic errors terminating Sierpinski triangles at corners are almost exclusively found under conditions E.

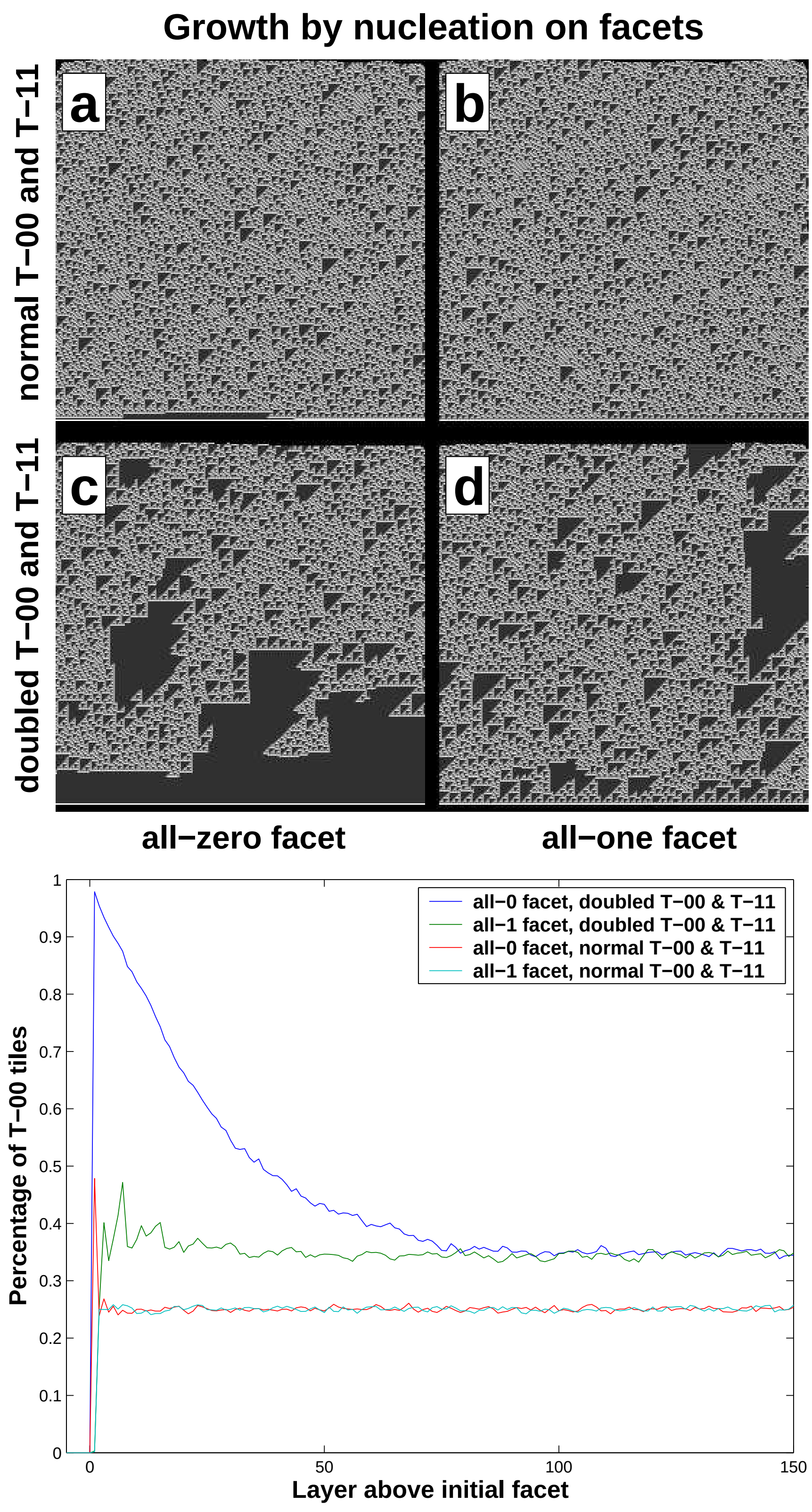

Figure S3: Simulations of growth on large facets. **(A-D)** Example runs. The bottom row is the pre-existing facet (256 tiles) presenting either all '0' bond types or all '1' bond types. The T-00 and T-11 tiles were either present at the normal concentration (as in Figure 2B and 2E) or at double the normal concentration (as in Figure 2C and 2F). Simulations were performed at $G_{mc} = 17.0$ and $G_{se} = 8.6$, as in Figure 2C. Orientation of the tiles is as in Figure S1C. **(bottom)** Probability of observing a T-00 tile L layers above the facet, for each of the four cases, estimated from 100 runs.

```
DAE-E system strands:

Rule tile strands.

 VE1          (37-mer,  377840 /M/cm @ 260nm) : CCATTCGGACGTTTGCGGTAAAGATTAGGACATTGAA
 VE2_EE00     (26-mer,  260540 /M/cm @ 260nm) : CTGGTTCCGAGCACCGAATGGAGGTA
 VE3          (42-mer,  412740 /M/cm @ 260nm) : TTACCGCAAACGTGGCGAGTGTGATACGACTACACCTAATCT
 VE4_EE00     (26-mer,  249800 /M/cm @ 260nm) : ACCAGTTCAATGTGGCGTTCATACCT
 VE5          (37-mer,  348140 /M/cm @ 260nm) : TGAACGCCTGTAGTCGTATCACACTCGCCTGCTCGGA

 UE1          (37-mer,  374540 /M/cm @ 260nm) : CGTTAAGGACGACGCAATTCTCACATCGGACGAGTAG
 UE2_EE11     (26-mer,  254240 /M/cm @ 260nm) : GTCTGTGGTTTCACCTTAACGAGGTA
 UE3          (42-mer,  404820 /M/cm @ 260nm) : AGAATTGCGTCGTGGTTGCTAGGTCTCGCTATCACCGATGTG
 UE4_EE11     (26-mer,  253840 /M/cm @ 260nm) : ACCAGCTACTCGTGGATCTATAATGC
 UE5          (37-mer,  378680 /M/cm @ 260nm) : ATAGATCCTGATAGCGAGACCTAGCAACCTGAAACCA

 RE1J         (59-mer,  553620 /M/cm @ 260nm) : CGTATTGGACATTTGCTCAGCGTTTTCGCTGAGCTTCCGTAGACCGACTGGACATCTTC
 RE1          (37-mer,  356360 /M/cm @ 260nm) : CGTATTGGACATTTCCGTAGACCGACTGGACATCTTC
 RE2_EE01     (26-mer,  242720 /M/cm @ 260nm) : CTGGTCCTTCACACCAATACGGCATT
 RE3          (42-mer,  430880 /M/cm @ 260nm) : TCTACGGAAATGTGGCAGAATCAATCATAAGACACCAGTCGG
 RE4          (26-mer,  273000 /M/cm @ 260nm) : CAGACGAAGATGTGGTAGTGGAATGC
 RE5          (37-mer,  348160 /M/cm @ 260nm) : CCACTACCTGTCTTATGATTGATTCTGCCTGTGAAGG
 RE5J         (59-mer,  549780 /M/cm @ 260nm) : CCACTACCTGTCTTCTTGCGACTTTTGTCGCAAGTTATGATTGATTCTGCCTGTGAAGG

 SE1J         (59-mer,  572120 /M/cm @ 260nm) : CTCAGTGGACAGCCTACTTACCTTTTGGTAAGTATTGTTCTGGAGCGTTGGACGAAACT
 SE1          (37-mer,  360300 /M/cm @ 260nm) : CTCAGTGGACAGCCGTTCTGGAGCGTTGGACGAAACT
 SE2          (26-mer,  256620 /M/cm @ 260nm) : GTCTGGTAGAGCACCACTGAGGCATT
 SE3          (42-mer,  415380 /M/cm @ 260nm) : CCAGAACGGCTGTGGCTAAACAGTAACCGAAGCACCAACGCT
 SE4_EE10     (26-mer,  249220 /M/cm @ 260nm) : CAGACAGTTTCGTGGTCATCGTACCT
 SE5          (37-mer,  336840 /M/cm @ 260nm) : CGATGACCTGCTTCGGTTACTGTTTAGCCTGCTCTAC
 SE5J         (59-mer,  539060 /M/cm @ 260nm) : CGATGACCTGCTTCATGTCGGCTTTTGCCGACATTTGGTTACTGTTTAGCCTGCTCTAC

Cap and input tile strands for use with R-type nucleating strands.

 CapNRERE     (37-mer,  398960 /M/cm @ 260nm) : GATAGATGAGAGATTGAGTATAGTGTTGTTTGATAAG
 CapNUERE     (37-mer,  400000 /M/cm @ 260nm) : AGTGAATAGAAATGAATTGTAAAGTTGTGAGGTGTTA

 NRE1         (37-mer,  376320 /M/cm @ 260nm) : ATGCCAGGACGTTCGCAGCAGTCAACAGGACGATCAA
 NRE2         (26-mer,  261360 /M/cm @ 260nm) : TGGTTAGTTTGGACCTGGCATAGGTA
 NRE3         (42-mer,  424300 /M/cm @ 260nm) : CTGCTGCGAACGTGGAAGTGATGTAAGATATGGACCTGTTGA
 NRE4         (26-mer,  266160 /M/cm @ 260nm) : CAGACTTGATCGTGGTAGGTGGATTA

 NUE1         (37-mer,  382040 /M/cm @ 260nm) : CGAACTGGACGAAGGCAAGCGTGACAAGGACCGTTAG
 NUE2         (26-mer,  268540 /M/cm @ 260nm) : TGGTTGATGGAGACCAGTTCGAGGTA
 NUE3         (42-mer,  404120 /M/cm @ 260nm) : CGCTTGCCTTCGTGGATTTGAATGGTAATGTAGACCTTGTCA
 NUE4         (26-mer,  272940 /M/cm @ 260nm) : ACCAGCTAACGGTGGTTAAGAGTAGG

Splint strands for making R-type nucleating strands with assembly PCR.

 SplintNREUE2 (40-mer,  414660 /M/cm @ 260nm) : GTGTTGTTTGATAAGTGGTTGATGGAGAGGATTTGAATGG
 SplintNUERE2 (40-mer,  419340 /M/cm @ 260nm) : AGTTGTGAGGTGTTATGGTTAGTTTGGAGGAAGTGATGTA

 SplintNUEUE2 (40-mer,  418300 /M/cm @ 260nm) : AGTTGTGAGGTGTTATGGTTGATGGAGAGGATTTGAATGG
 SplintNRERE1 (40-mer,  441320 /M/cm @ 260nm) : GTAAGATATGGAGGTAGGTGGATTAGATAGATGAGAGATT
 SplintNUERE1 (40-mer,  443880 /M/cm @ 260nm) : TGGTAATGTAGAGGTTAAGAGTAGGAGTGAATAGAAATGA
 BridgeNRERE  (47-mer,  455640 /M/cm @ 260nm) : AACCACTTATCAAACAACACTATACTCAATCTCTCATCTATCTAATC
 BridgeNUERE  (47-mer,  446840 /M/cm @ 260nm) : AACCATAACACCTCACAACTTTACAATTCATTTCTATTCACTCCTAC

 NRE5         (37-mer,  335860 /M/cm @ 260nm) : CACCTACCTCCATATCTTACATCACTTCCTCCAAACT
 NUE5         (37-mer,  339240 /M/cm @ 260nm) : TCTTAACCTCTACATTACCATTCAAATCCTCTCCATC
```

Figure S4: DAE-E sequences.

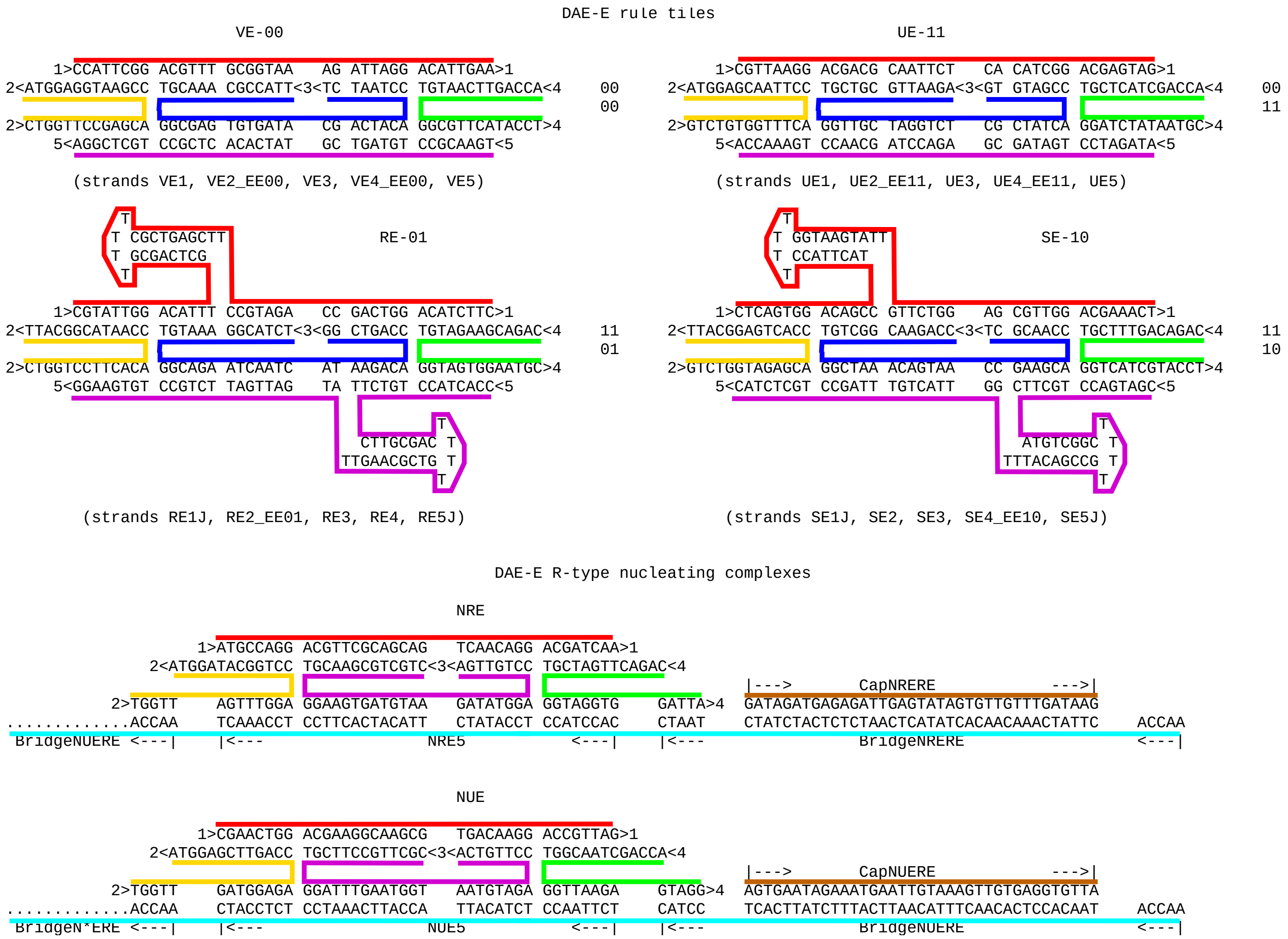

Figure S5: DAE-E diagrams. Arrows point 5′ to 3′. Component subsequences of the nucleating strand are indicated.

```
DAO-E system strands:

Rule tile strands.

 R00_1    (26-mer,  255580 /M/cm @ 260nm) : TCACTCTACCGCACCAGAATGGAGAT
 R00_2    (48-mer,  460620 /M/cm @ 260nm) : CATTCTGGACGCCATAAGATAGCACCTCGACTCATTTGCCTGCGGTAG
 R00_3    (48-mer,  477220 /M/cm @ 260nm) : CAGTAGCCTGCTATCTTATGGCGTGGCAAATGAGTCGAGGACGGATCG
 R00_4    (26-mer,  248640 /M/cm @ 260nm) : TCACTCGATCCGTGGCTACTGGAGAT

 S00_1    (26-mer,  254080 /M/cm @ 260nm) : AGTGAGGCAATCCACAACCGCATCTC
 S00_2    (48-mer,  465300 /M/cm @ 260nm) : GCGGTTGTCCAACTTACCAGATCCACAAGCCGACGTTACAGGATTGCC
 S00_3    (48-mer,  456880 /M/cm @ 260nm) : GCTCTACAGGATCTGGTAAGTTGGTGTAACGTCGGCTTGTCCGTTCGC
 S00_4    (26-mer,  266060 /M/cm @ 260nm) : AGTGAGCGAACGGTGTAGAGCATCTC

 R11_1    (26-mer,  235900 /M/cm @ 260nm) : TCACTCAAACGCACCACTCTGTCTTG
 R11_2    (48-mer,  472980 /M/cm @ 260nm) : CAGAGTGGACGAAAGCTCACGGCACCAAGTATCAGGTTCCTGCGTTTG
 R11_3    (48-mer,  458120 /M/cm @ 260nm) : CTGTAGCCTGCCGTGAGCTTTCGTGGAACCTGATACTTGGACGAGTTG
 R11_4    (26-mer,  240840 /M/cm @ 260nm) : TCACTCAACTCGTGGCTACAGTCTTG

 S11_1    (26-mer,  244160 /M/cm @ 260nm) : GTATGGCTCGGCACCTCAAACATCTC
 S11_2    (48-mer,  474920 /M/cm @ 260nm) : GTTTGAGGACGCTATGAACATCCACCTAAGCAGAGACACCTGCCGAGC
 S11_3    (48-mer,  465880 /M/cm @ 260nm) : CGAGTACCTGGATGTTCATAGCGTGGTGTCTCTGCTTAGGACGAATGC
 S11_4    (26-mer,  248380 /M/cm @ 260nm) : GTATGGCATTCGTGGTACTCGATCTC

 R01n_1   (26-mer,  261440 /M/cm @ 260nm) : CATACCGTTGGCACCGAAAGCGAGAT
 R01n_2   (48-mer,  442820 /M/cm @ 260nm) : GCTTTCGGACTCGATCTCCAGACACCTACTGCGGTTCACCTGCCAACG
 R01n_2JC (70-mer,  640400 /M/cm @ 260nm) : GCTTTCGGACTCGATCTCCGCTGCTTTTGCAGCGGATTTCCAGACACCTACTGCGGTTCACCTGCCAACG
 R01n_3JC (70-mer,  671480 /M/cm @ 260nm) : CGATGACCTGTCTGGAGCTACCGCTTTTGCGGTAGCTTGATCGAGTGGTGAACCGCAGTAGGACGCCTCG
 R01n_3   (48-mer,  473220 /M/cm @ 260nm) : CGATGACCTGTCTGGAGATCGAGTGGTGAACCGCAGTAGGACGCCTCG
 R01n_4   (26-mer,  248740 /M/cm @ 260nm) : CATACCGAGGCGTGGTCATCGTCTTG

 S01_1    (26-mer,  272900 /M/cm @ 260nm) : AGTGAGAACGACCACATCATCCAAGA
 S01_2    (48-mer,  456960 /M/cm @ 260nm) : GATGATGTCCTTGTAAACTTCGCCACTCTAATCGCAATCAGGTCGTTC
 S01_2JC  (70-mer,  655520 /M/cm @ 260nm) : GATGATGTCCTTGTAACGCTCTGCTTTTGCAGAGCGTTACTTCGCCACTCTAATCGCAATCAGGTCGTTC
 S01_3JC  (70-mer,  702640 /M/cm @ 260nm) : GAGCAACAGGCGAAGTCTCCATCGTTTTCGATGGAGTTTTACAAGGTGATTGCGATTAGAGTCCGTAAGC
 S01_3    (48-mer,  496340 /M/cm @ 260nm) : GAGCAACAGGCGAAGTTTACAAGGTGATTGCGATTAGAGTCCGTAAGC
 S01_4    (26-mer,  254480 /M/cm @ 260nm) : GTATGGCTTACGGTGTTGCTCCAAGA

Cap and input tile strands for use with R-type nucleating strands.

 cpBr1    (37-mer,  387260 /M/cm @ 260nm) : GTTGATGGAGTATAGTGTATTGGATGAAATGTTATGT
 A1S      (37-mer,  356120 /M/cm @ 260nm) : TCACTGCTGAAGGCAGAGGACTGTGCTGGACTTGGTC
 A2       (28-mer,  268000 /M/cm @ 260nm) : TGGTAATGTAAGGACCTCTGCCTTCAGC
 A4SV     (26-mer,  267800 /M/cm @ 260nm) : CATACGACCAAGTGGATTTGTAGGAT
 A4_S00   (26-mer,  261380 /M/cm @ 260nm) : TCACTGACCAAGTGGATTTGTAGGAT
 A3_nick  (20-mer,  203520 /M/cm @ 260nm) : GGTTGAATGACCAGCACAGT

Splint strands for making nucleating strands with assembly PCR.

 Sp1A     (40-mer,  422100 /M/cm @ 260nm) : TGAATGAGGATTTGTAGGATGTTGATGGAGTATAGTGTAT
 SpA1     (40-mer,  421860 /M/cm @ 260nm) : TATTGGATGAAATGTTATGTTGGTAATGTAAGGAGGTTGA
 Br1      (37-mer,  365600 /M/cm @ 260nm) : ACATAACATTTCATCCAATACACTATACTCCATCAAC
 A5       (37-mer,  350140 /M/cm @ 260nm) : ATCCTACAAATCCTCATTCAACCTCCTTACATTACCA
```

Figure S6: DAO-E sequences.

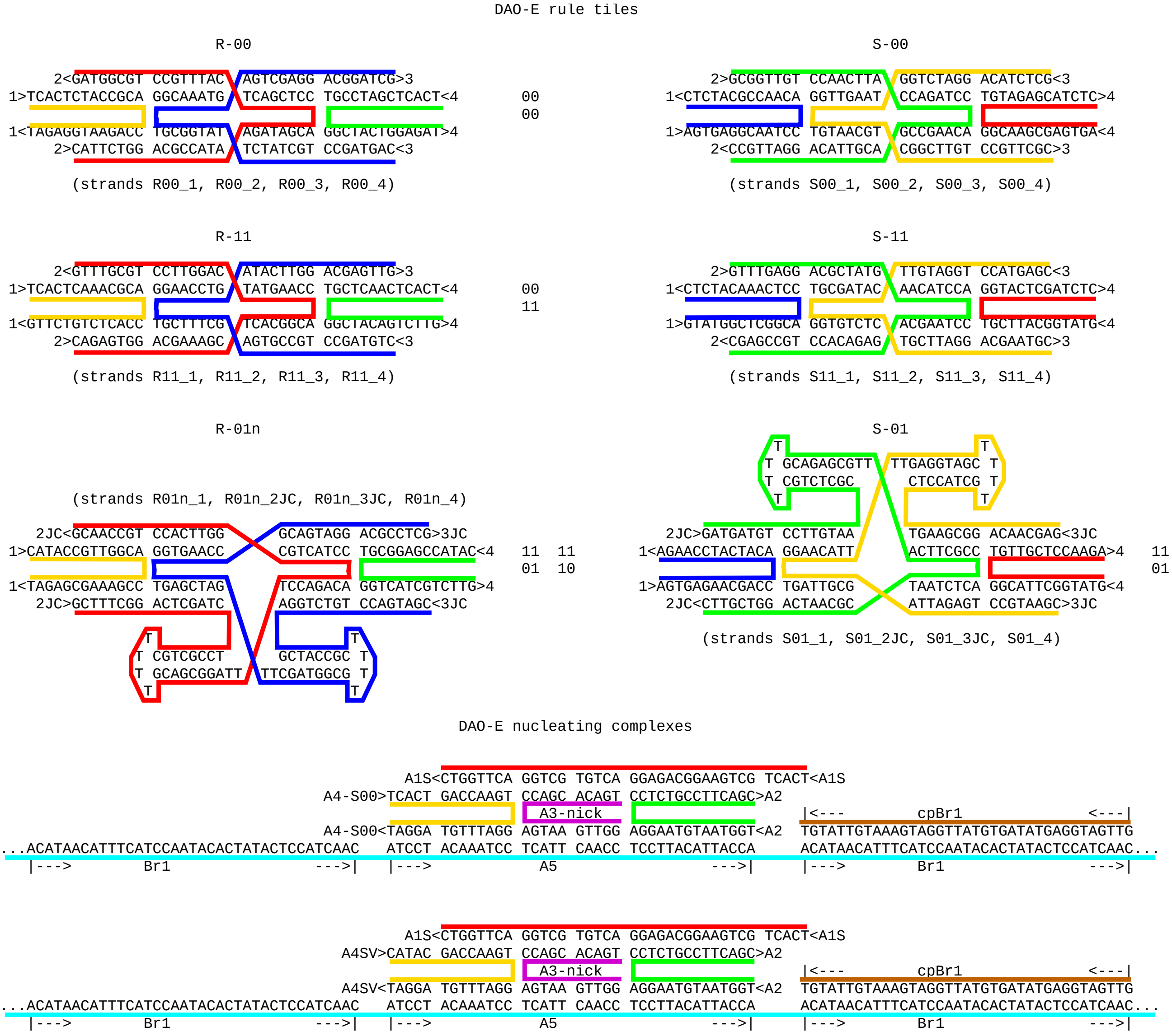

Figure S7: DAO-E diagrams. Arrows point 5′ to 3′. Component subsequences of the nucleating strand are indicated.

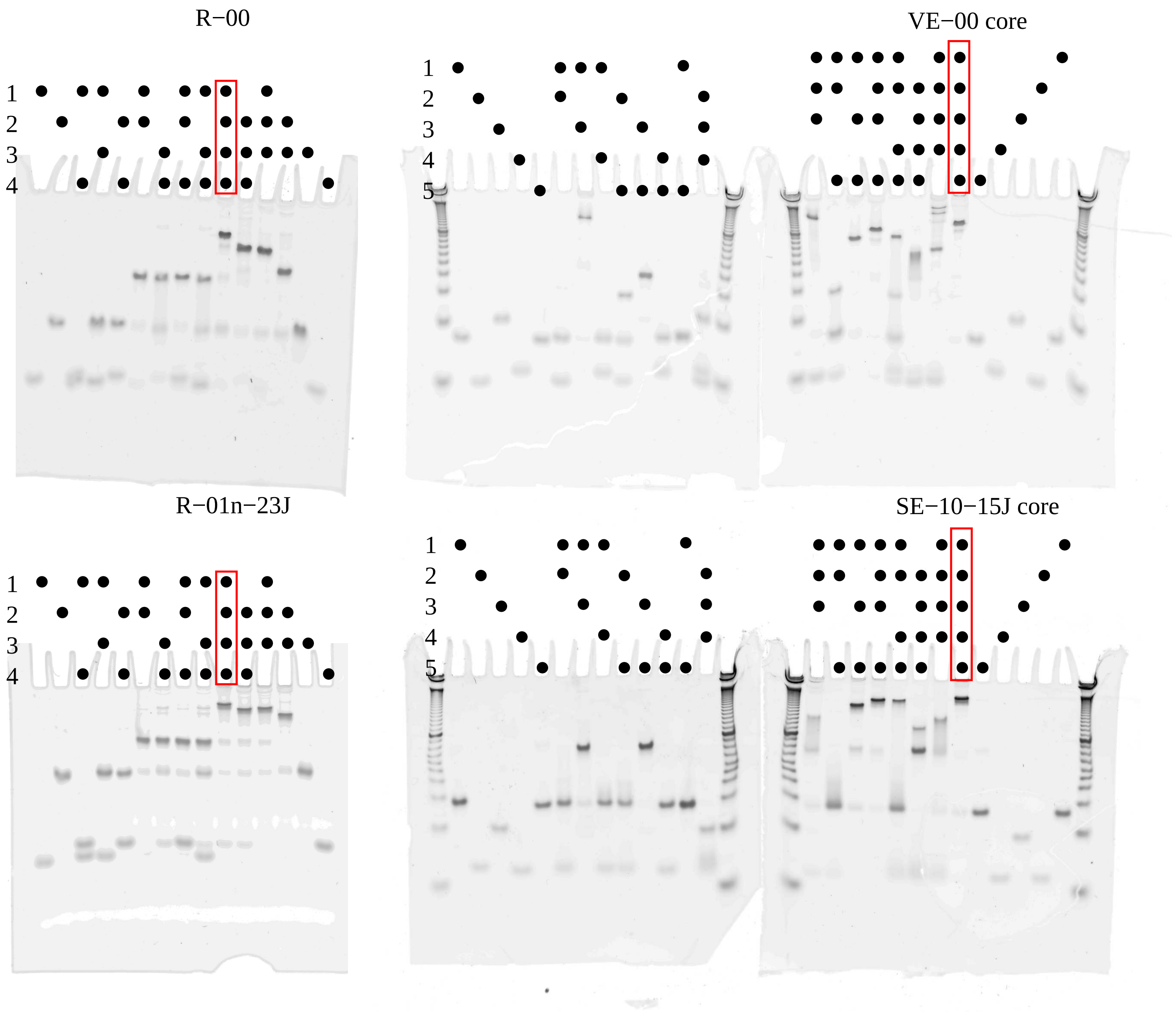

Figure S8: Formation gels for representative DAO-E and DAE-E tiles. Dots above each lane indicate which combination of strands was included in the annealing reaction. In most lanes, strands associate according to designed interactions only; e.g., in the DAO-E tiles, strands 2 and 4 run separately, while strands 1 and 2 run as a single heavy species. The red box indicates the lane containing all species, which should therefore form double-crossover molecules running as a single band. DAE-E formation gels are shown for tiles with different sticky ends but the same cores as VE-00 and SE-10-15J. Specifically, `VE2 (26-mer, 252260 /M/cm) CTGGTTCCGAGCACCGAATGGATACC`, `VE4 (26-mer, 251060 /M/cm) TGAGGTTCAATGTGGCGTTCATACCT`, and `SE4 (26-mer, 251920 /M/cm) TGAGGAGTTTCGTGGTCATCGTACCT` were used in place of the correspondingly-numbered strands.

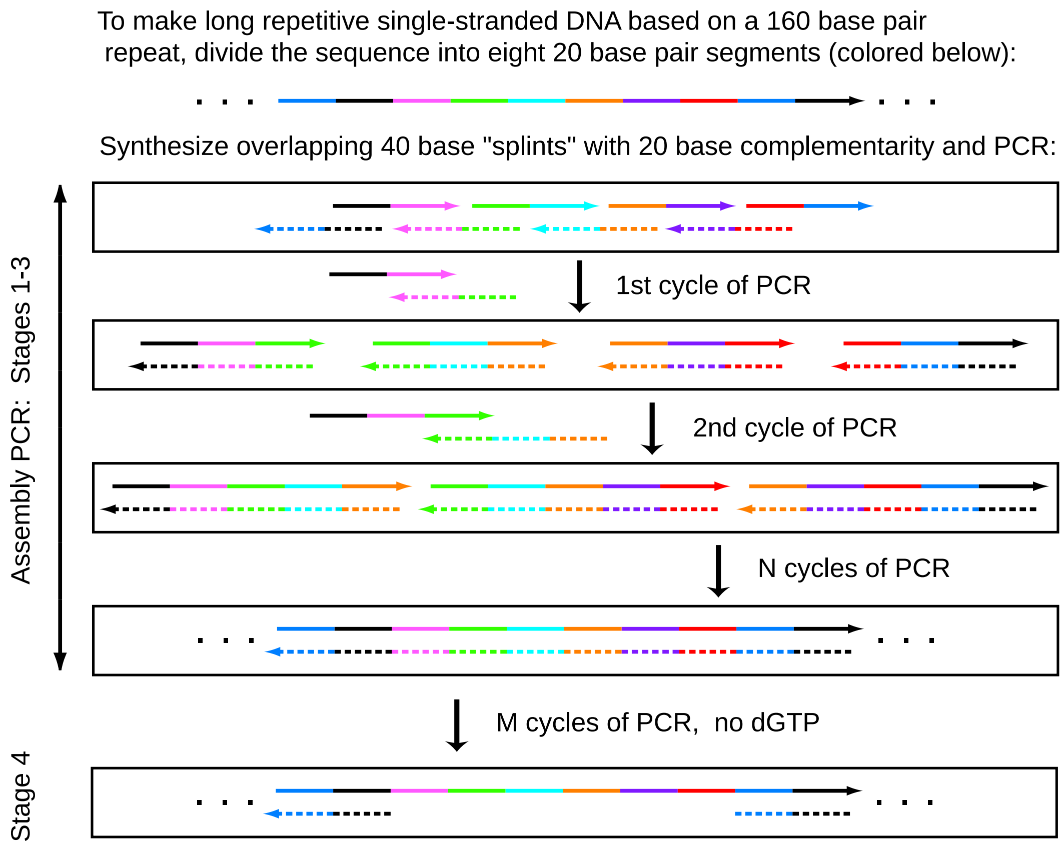

Figure S9: Using assembly PCR to generating long, repetitive, single-stranded DNA.

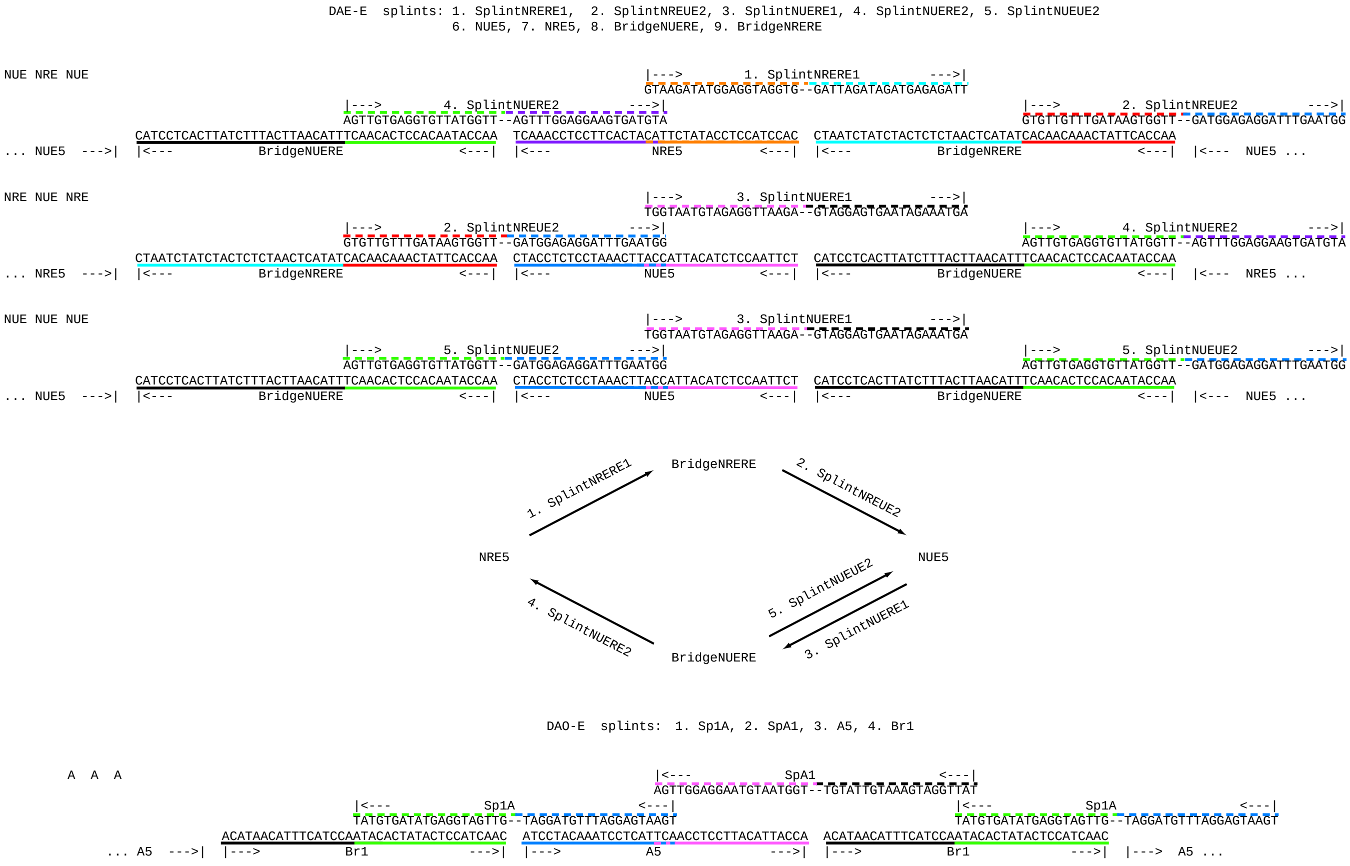

Figure S10: Assembly PCR scheme for DAE-E and DAO-E nucleating strands. In each case the bottom strand (solid) is the all-ACT nucleating strand drawn in an orientation to match the previous schema. In the DAE-E nucleating strand construction, due to the splint strands used in assembly PCR, BridgeNUERE always appears 3′ of NRE5, while either BridgeNUERE or BridgeNRERE may appear 3′ of NUE5. Thus, the sequence of input tiles determined by each nucleating strand is in the regular language (NRE NUE^{+})*, as illustrated by the state transition diagram. The density of NRE5 subsequences (which output a ‘1’ to their right) is determined by the proportion of SplintNREUE2 and SplintNUERE2 relative to other strands in the assembly PCR. In the DAO-E nucleating strand construction, there is a single repeating sequence. The density of input tiles outputting a ‘1’ is determined by the proportion of A4SV relative to A4-S00 in the input strand mix used during annealing.

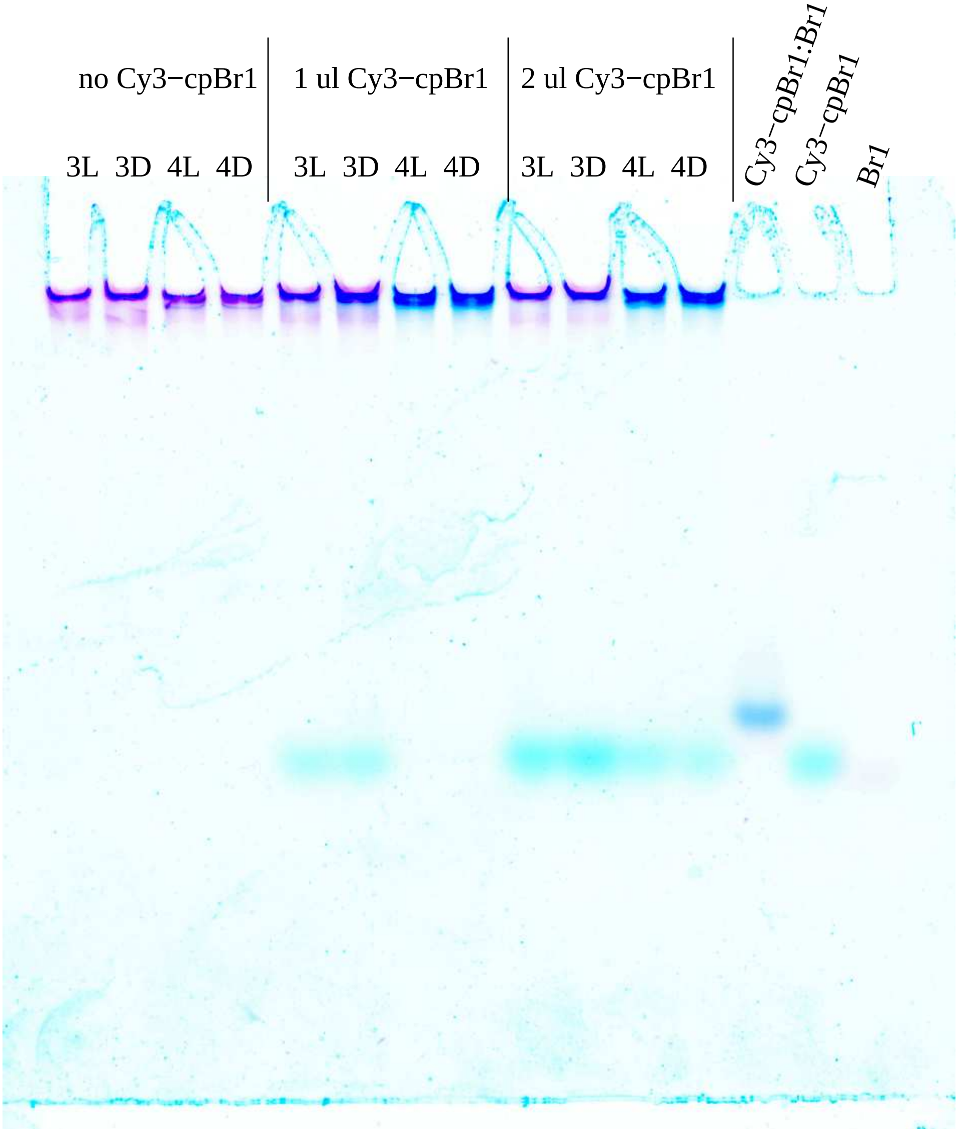

Figure S 11: Binding capacity gel for determining DAO-E nucleating strand stoichiometry. Lanes designated ‘3’ contain double-stranded material purified after stage 3, lanes designated ‘4’ contain material purified after stage 4. Lanes designated ‘L’ had Sybr Green I added to the reaction mixture prior to PCR, and lanes designated ‘D’ had no Sybr Green I at this stage. The first set of four lanes acts as controls, demonstrating how the products of both stage 3 and stage 4 remain stuck in the wells. The second set of four lanes had 1 μL of Cy3-labelled cpBr1 added. The third set of four lanes had 2 μL of Cy3-labelled cpBr1 added. The final three lanes are controls: Cy3-cpBr1 complexed with its complement Br1, Cy3-cpBr1, and Br1 alone. The gel was post-stained with Sybr Green I and imaged under two conditions: (1) excitation with a 488 nm laser with emission recorded by a 530 nm bandpass filter resulting in the purple lanes—this captures the Sybr Green I emission and (2) excitation with a 532 nm laser with emission recorded by a 555 nm longpass filter resulting in the blue bands—this captures the Cy3 emission. Cyan false-color indicates fluorescence of Cy3-cpBr1. Purple false-color indicates fluorescence of Sybr Green I stain, which preferentially stains double-stranded material. For example, Br1 has the same mobility as Cy3-cpBr1, but stains only faintly.

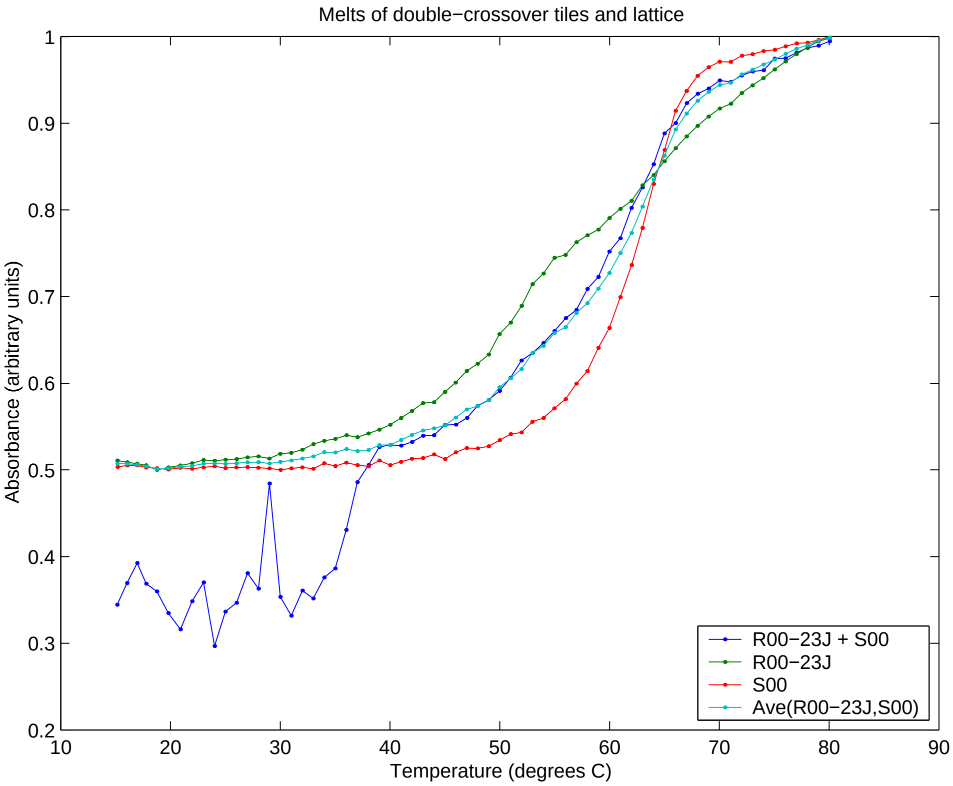

Figure S 12: Melts of R-00-23J and S-00 and their mixture. Tile R-00-23J has the same core as R-00, but replaces the correspondingly-numbered strands by `R00-2J (70-mer, 664820 /M/cm) CATTCTGGACGCCACGGTCAAGTTTTCTTGACCGTTTAAGATAGCACCTCGACTCATTTGCCTGCGGTAG`, and `R00-3J (70-mer, 681480 /M/cm) CAGTAGCCTGCTATCGGTTGTGTTTTCACAACCGTTCTTATGGCGTGGCAAATGAGTCGAGGACGGATCG`. Absorbance values were normalized to the maximum and minimum of the single-tile curves. The average of the R-00-23J curve and the S-00 curve is drawn in cyan; above 40°C it agrees with the melting curve of the R-00-23J + S-00 mixture, indicating that the melting temperature of this crystal is below 40°C at 0.2 μM.

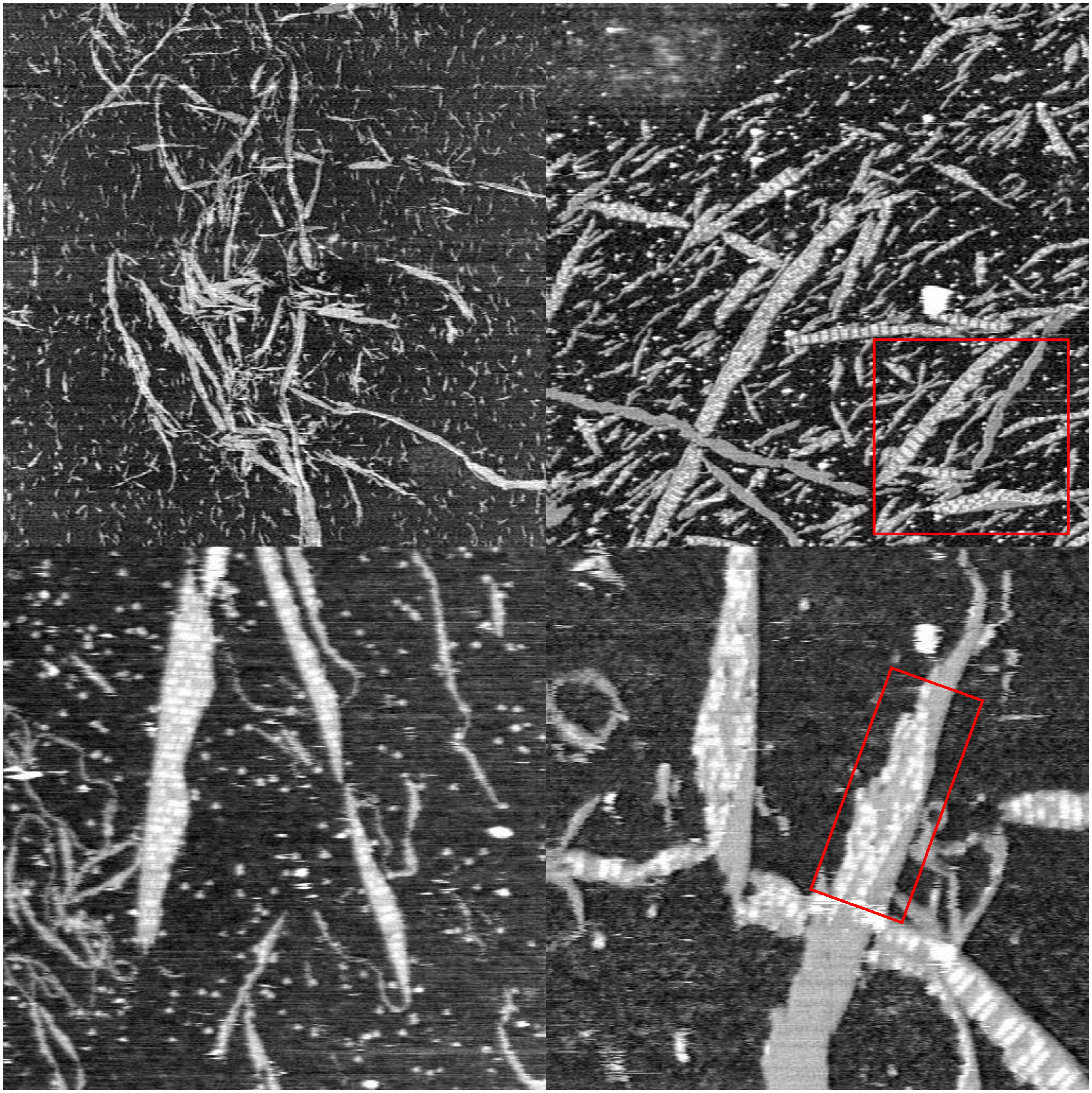

Figure S13: AFM images showing the context and distribution of DAE-E crystals. Upper left: 5.0 μm scan showing many long, thin crystals. Upper right: 2.3 μm scan showing the region surrounding Figure 5A (red box). Lower left: 830 nm scan showing faceting of templated crystals. Note the thin tails extending from several of the crystals. These may be regions of the nucleating strand / input tile complexes that have not yet grown as part of the crystals, or they may be regions of the nucleating strand that remain double-stranded after the asymmetric PCR step of the assembly PCR protocol. Lower right: 650 nm scan showing the region surrounding Figure 5C (red box).

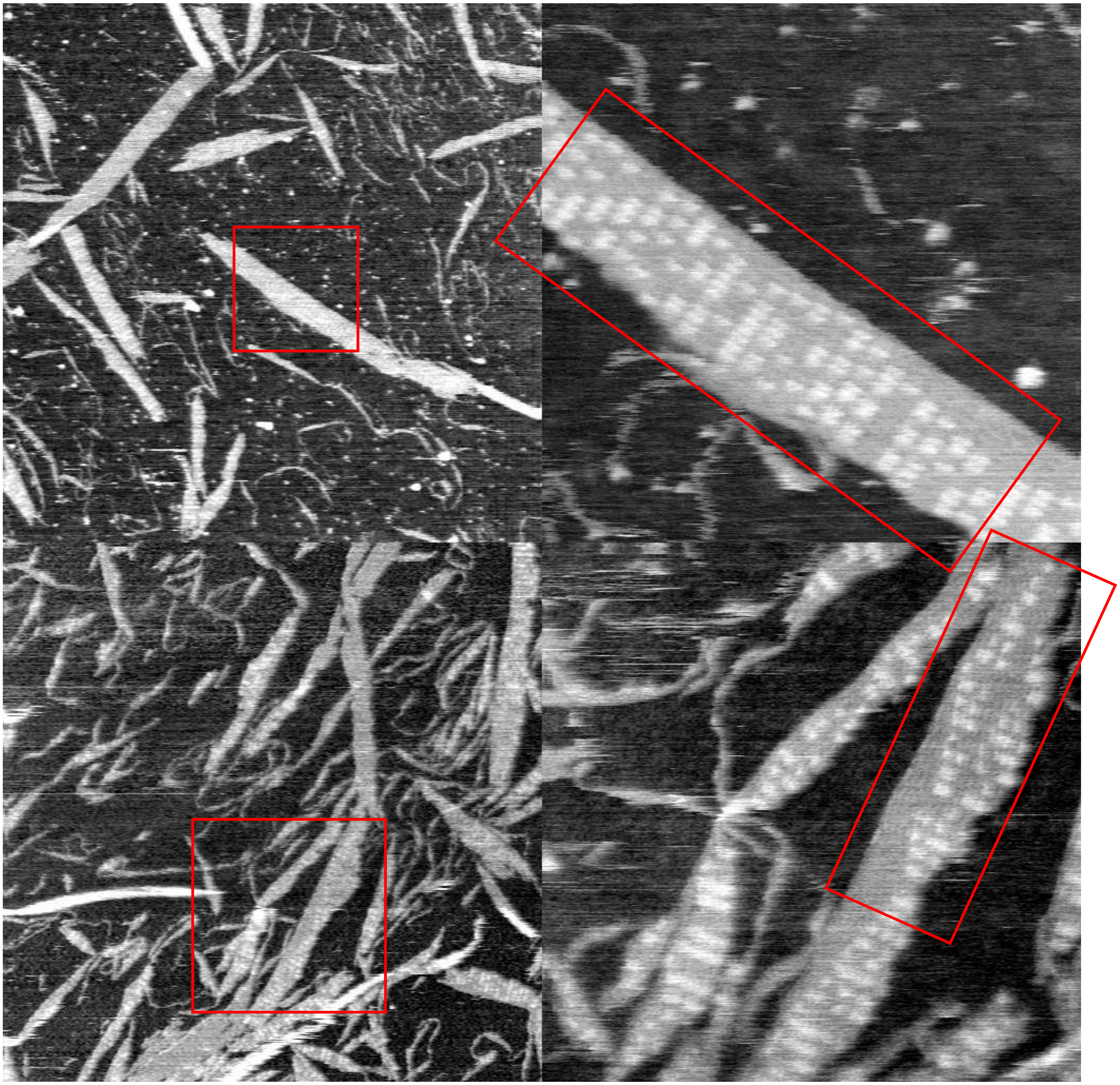

Figure S14: AFM images showing the context and distribution of DAE-E crystals. Upper left: 1.5 μm scan showing the region surrounding Figure 5B. (Red box shows area of upper right scan.) Upper right: 320 nm scan showing the region surrounding Figure 5B (red box). Lower left: 1.3 μm scan showing the region surrounding Figure 5D. (Red box shows area of lower right scan.) Lower right: 430 nm scan showing the region surrounding Figure 5D (red box).

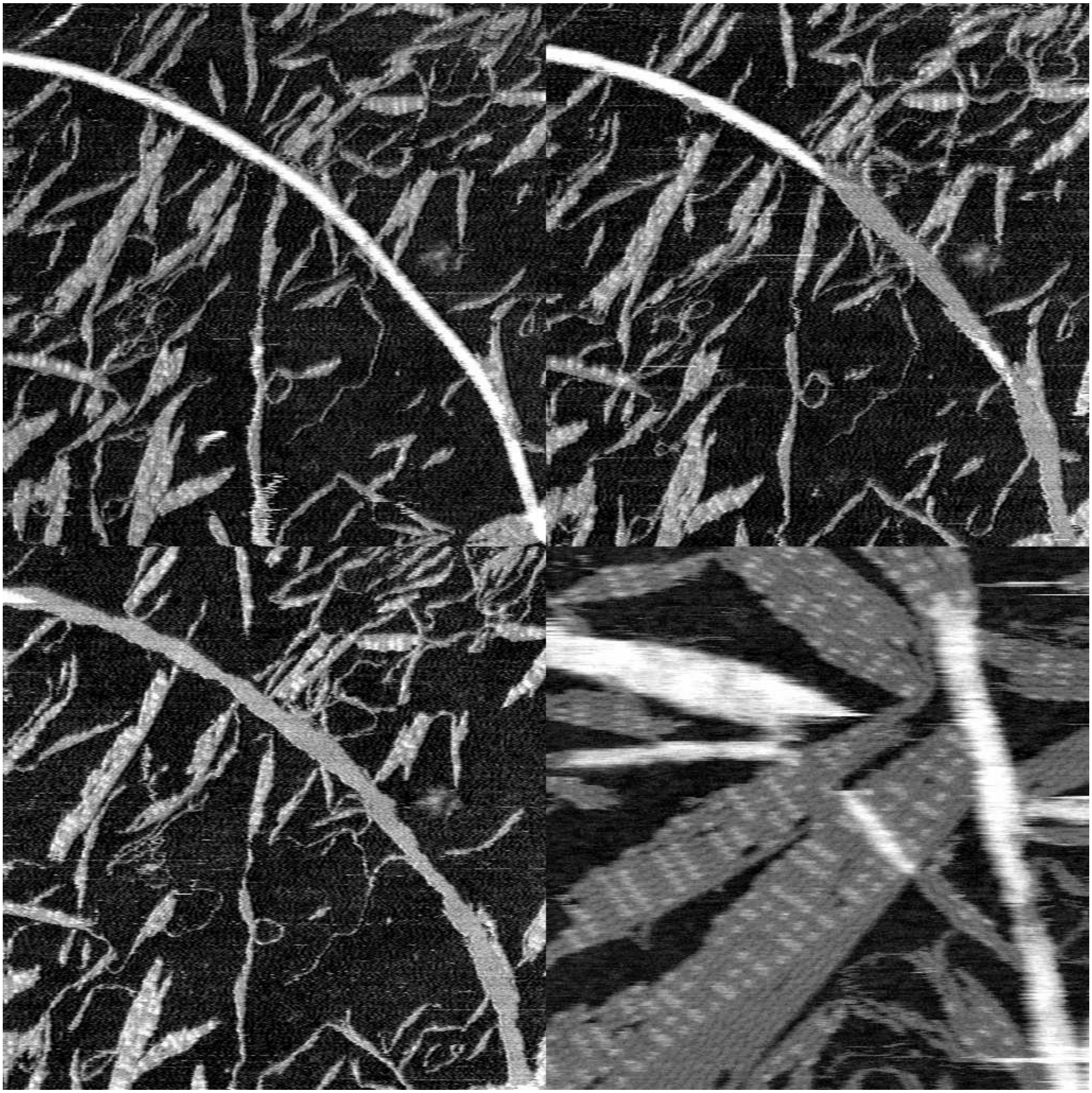

Figure S15: AFM images of DAE-E crystals and tubes. Upper left: 1.0 μm scan showing an unopened tube. The tube is roughly twice the height of other crystals. Upper right: Subsequent scan shows the tube partially opened. Opened domains are the same height as other crystals; closer examination reveals tiles whose long axis parallels the tube axis. Lower left: An even later scan of the same region reveals the tube completely opened. Lower right: 390 nm scan showing the region surrounding Figure 5E. Three unopened tubes (with circumferences of roughly 4, 8, and 17 tiles) can also be seen.

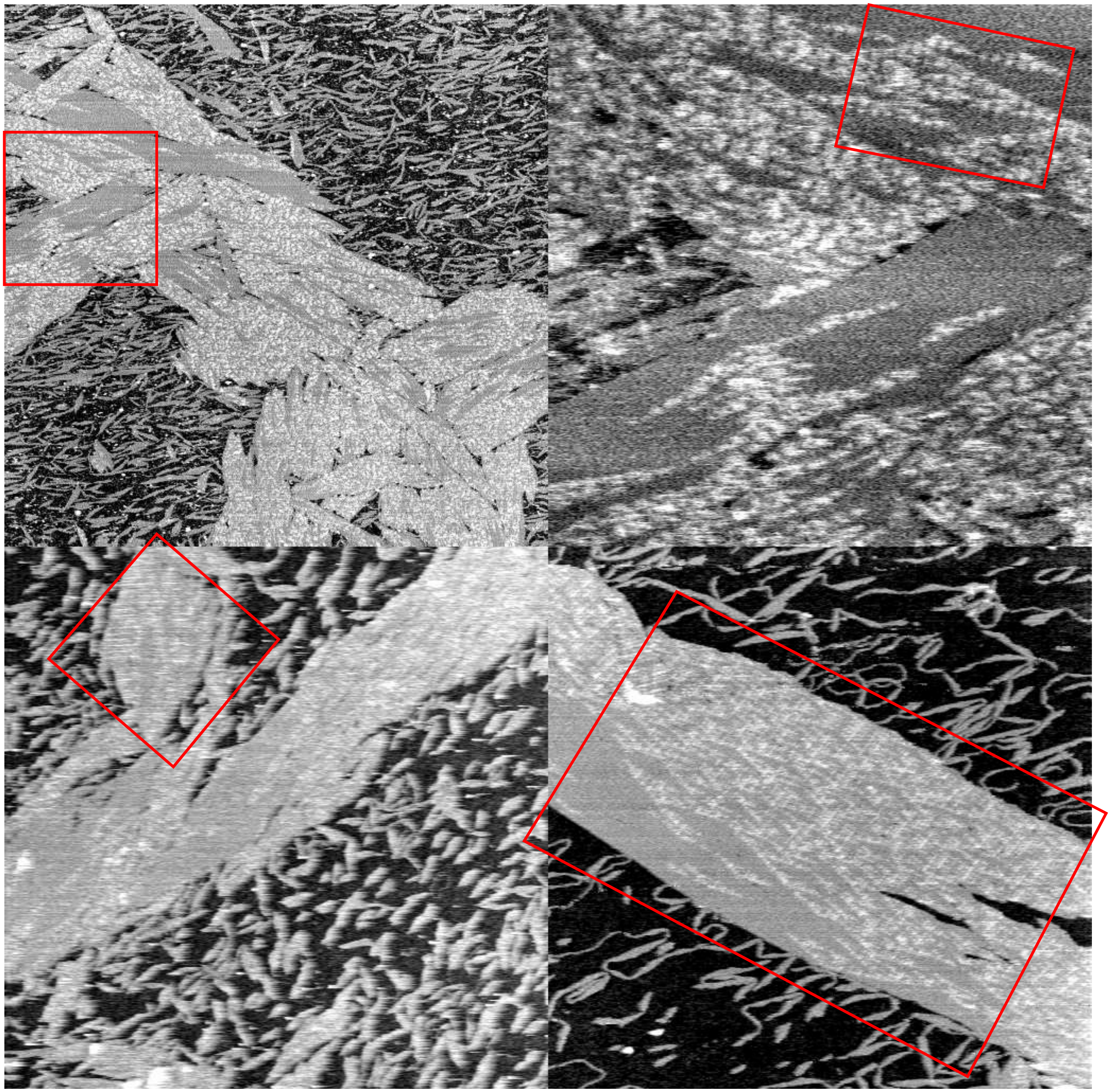

Figure S16: AFM images showing the context and distribution of DAO-E crystals. Upper left: 4.0 μm scan showing region surrounding Figure 6C. (Red box shows area of upper right scan.) Upper right: 500 nm scan showing region surrounding Figure 6D (red box). Lower left: 2.3 μm scan showing region surrounding Figure 6B (red box). Lower right: 1.8 μm scan showing the region surrounding Figure 6A (red box).

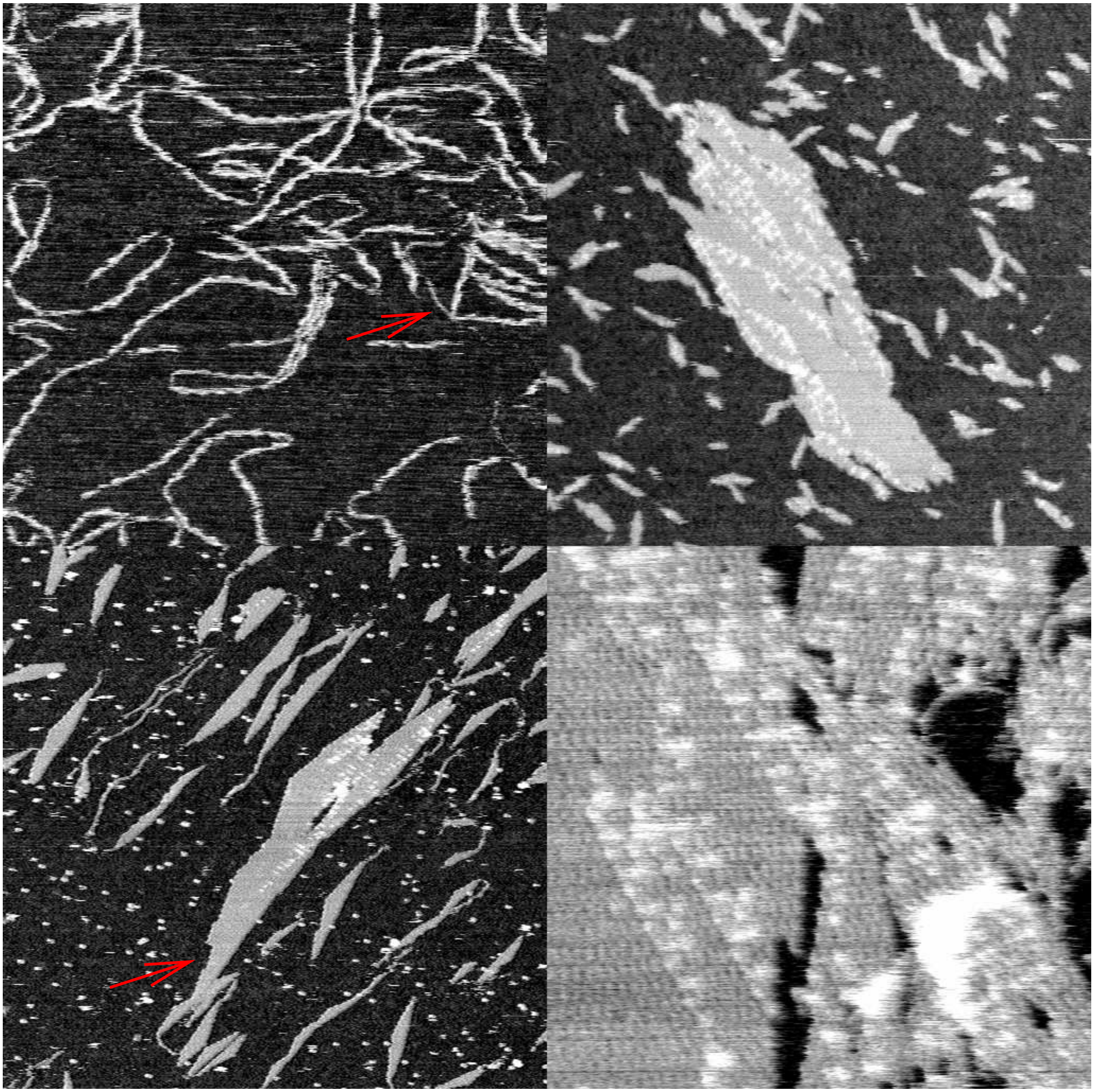

Figure S 17: AFM images of boundary assemblies and untemplated DAO-E crystals. Upper left: 750 nm scan showing nucleating strand + input tiles + S-00. Bumpy domains indicate the presence of input tiles and one layer of S-00. Thinner smooth domains (arrow) are assumed to be double-stranded, and hence without tiles. Upper right: 1.1 μm scan of a sample prepared with just five tiles (no S-11) and no nucleating structures. Therefore, this must be an untemplated crystal. (It could not be a ripped fragment of a templated crystal.) Lower left: 1.2 μm scan of a sample prepared with all six tiles. Nucleating structure tails can be seen. Crystals are particularly well faceted. Facet roughening can be observed (arrow). Lower right: 320 nm scan of a sample prepared with just five tiles (no S-11).

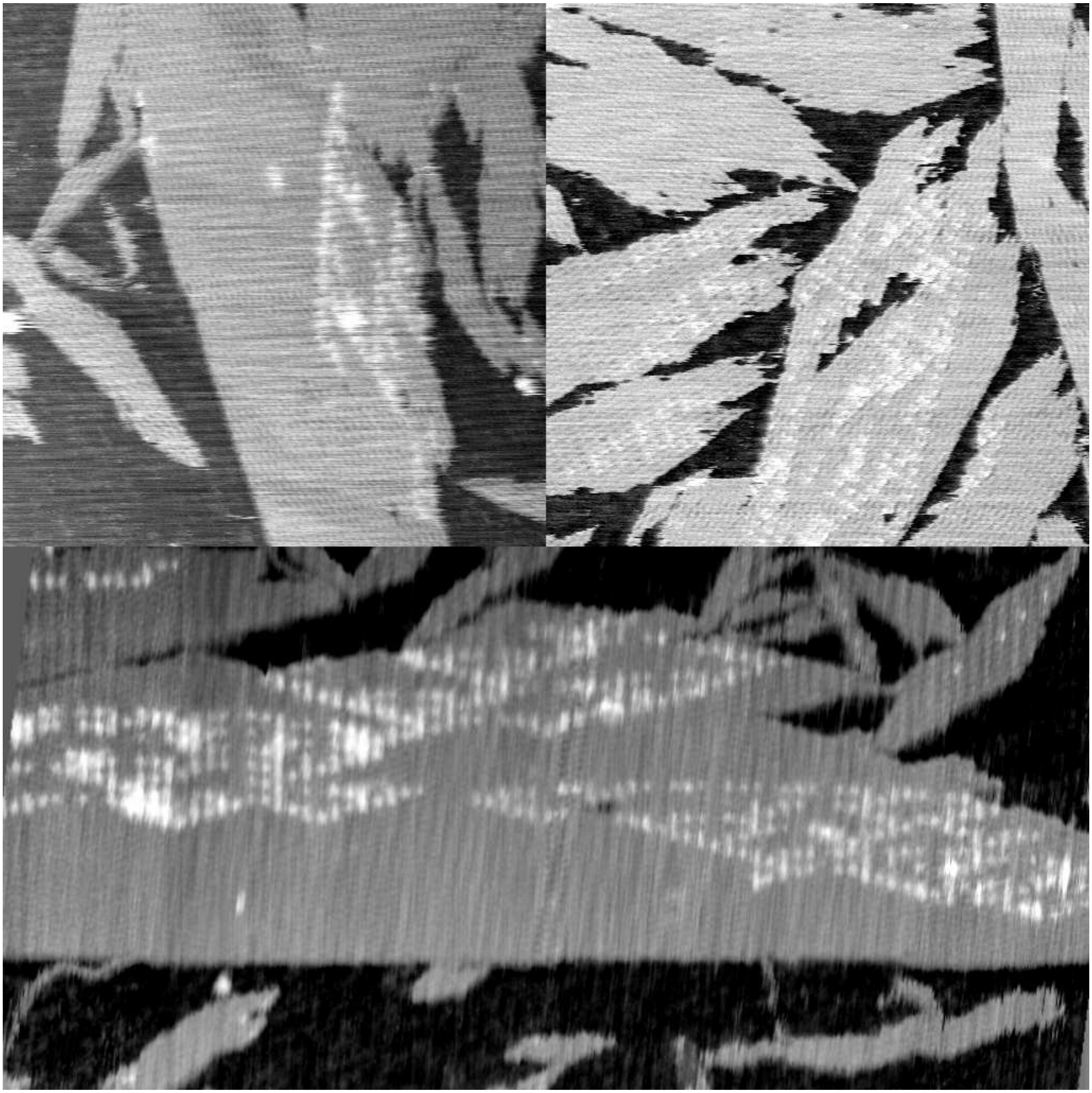

Figure S18: AFM images of DAO-E crystals grown under constant-temperature, near-constant concentration conditions. To construct thick rigid strips of ‘0’ tiles as initial templates for growth, all-‘0’ nucleating structures were bulk annealed with R-00 and S-00 tiles. These strips had variable width and often were faceted. Once room temperature had been reached, at roughly hourly intervals a mix of five pre-formed rule tiles were added to boost tile concentrations by 4 to 10 nM. Presumably, during the interval between additions, tiles incorporate into crystals and therefore their concentrations decrease to the critical concentration, which we estimate to be between 4 to 10 nM. Despite our hopes, this procedure did not lead to measurably lower error rates, perhaps due to “sideways” growth on facets. Upper left: 510 nm scan. Upper right: 550 nm scan. Lower: 980 nm image composite from three scans. Experiments performed by Jason Rolfe.
